# A Review of the Ethnopharmacology, Phytochemistry, Pharmacology, Application, Quality Control, Processing, Toxicology, and Pharmacokinetics of the Dried Rhizome of *Atractylodes macrocephala*

**DOI:** 10.3389/fphar.2021.727154

**Published:** 2021-11-03

**Authors:** Liu Yang, Huan Yu, Ajiao Hou, Wenjing Man, Song Wang, Jiaxu Zhang, Xuejiao Wang, Senwang Zheng, Hai Jiang, Haixue Kuang

**Affiliations:** Key Laboratory of Chinese Materia Medica, Heilongjiang University of Chinese Medicine, Ministry of Education, Harbin, China

**Keywords:** Baizhu, pharmacological effects, chemical composition, ethnopharmacology, processing, toxicity and pharmacokinetics

## Abstract

The product investigated herein is the dried rhizome of *Atractylodes macrocephala* Koidz. [Asteraceae] (Baizhu), which is also known as Dongbaizhu, Wuzhu, Yuzhu, Zhezhu, and Zhongzhu, among others. It invigorates the *spleen*, replenishes *qi*, and removes *dampness*, diuresis, and hidroschesis, and impacts fetal safety. It is often used for the treatment of diseases such as *spleen* function deficiency, abdominal distension, diarrhea, sputum, vertigo, edema, fever, and sweating and also aids cessation of minimal vaginal bleeding during pregnancy. In this study, research pertaining to the ethnopharmacology, application, phytochemistry, analytical methods, quality control, processing, pharmacology, toxicology, and pharmacokinetics of Baizhu has been reviewed. Relevant information and data reported for Baizhu were collected from CNKI, VIP, PubMed, Web of Science, scientific databases, Chinese Medicinal Material Encyclopedia, Chinese herbal medicine classics, Chinese medicine dictionary, doctoral and master’s theses, and so on. Baizhu demonstrates diuretic, antidiuretic, anti-inflammatory effects and antitumor function and aids regulation of gastrointestinal function, hypoglycemic effect, analgesic action, protection on the liver ischemia-reperfusion injury (IRI) in rats, inhibition of aromatase, treatment of bone disease, strengthening myocardial contraction ability, detoxification and cholagogic effect, fall hematic fat action, such as the treatment of acute renal injury, and so on. It also can be an anticoagulant, improve the nervous system disease, affect the immune system, and regulate uterine smooth muscle, antioxidation, antiaging, and antibacterial effect. Sesquiterpenoids, triterpenoids, polyacetylenes, phenylpropanoids, coumarins, flavonoids, flavonoid glycosides, steroids, benzoquinones, polysaccharides, and other compounds were isolated from Baizhu. Among them, sesquiterpenoids, polysaccharides, and polyacetylenes are the main components of Baizhu. Baizhu exhibits a wide range of pharmacological effects and constitutes a considerable proportion of the composition of many proprietary crude drugs. It mainly affects the endocrine, nervous, and urinary systems. The presented information suggests that we should focus on the development of new drugs related to Baizhu, including specific components, to achieve a greater therapeutic potential that can be considered to further explore the information related to Baizhu.

## Introduction

Baizhu is one of the most commonly used traditional Chinese medicines (TCMs) in China, reputed for application in TCM clinical practice. It is listed as the top-quality agent in Sheng Nong’s Herbal Classic (神农本草经) (Dong Han Dynasty, A.D. 25–220). Baizhu possesses *pungency*, *bitterness*, and *sweetness* in *flavor* and *warmness* in *nature*. It exhibits actions on the *spleen meridian* and *stomach meridian*. It is distributed in Jiangsu, Zhejiang, Fujian, Jiangxi, Anhui, Sichuan, Hubei, and Hunan provinces in China. Baizhu is cultivated or wild in these regions and mainly grows in mountainous and hilly areas. The rhizome is used as medicine, which is soaked in water or rice swill until soft and then cut and used in raw, stir-fry with bran, or stir-fry with soil. Baizhu is often confused with Cangzhu (*Atractylodes lancea* (Thunberg) Candolle or *A. chinensis* (Candolle) Koidzumi dry rhizomes.), and a gene identification technique is necessary to clarify the quality of the same genus plants in pharmacological evaluation (Shimato et al., 2018). We have added information on the differences between the original plant species of Baizhu and Cangzhu (Supplementary Table S1). Studies have shown that Baizhu is used for ameliorating deficiency syndrome of *spleen* weakness, and Cangzhu is used for the exuberance of interior *dampness*. Baizhu can reduce sweating and prevent miscarriage. Cangzhu can promote sweating for dispelling *wind pathogens* (Zhuang, 2018). Therefore, when using the two, we should distinguish between them to avoid confusion (Cangzhu: the taste is *pungency* and *bitterness*. It can help dispel *wind pathogens* and can help remove *dampness*. It is also extremely effective for treating symptoms such as inappetence. If the patient presents with night blindness and experiences regular pain in the limbs, the consumption of Cangzhu can help relieve the pain. Baizhu has a *sweetness* in *flavor*. It has the function of miscarriage prevention. In case of diarrhea and vomiting and other symptoms, Baizhu exhibits a beneficial effect).

Thus far, the clinical application of Baizhu is reportedly extensive, and more studies have been conducted at home and abroad. In summary, the pharmacological effects of Baizhu mainly include protective effects in liver ischemia-reperfusion injury (IRI) in rats, effects on gastrointestinal function, aromatase activity inhibition, inhibition of fat formation, anti-inflammatory effects, and antitumor effects (Chen, 2012). Concurrently, Baizhu also demonstrates a variety of pharmacological effects, such as analgesic effects, and exerts influence on the immune system, thus presenting with a wide range of clinical applications.

In this study, the ethnopharmacology, phytochemistry, pharmacological activity, quality control, application, toxicology, and pharmacokinetics of Baizhu have been discussed comprehensively. Additionally, comprehensive understanding of Baizhu was achieved which will help provide relevant information and lay the basis for further research and development of Baizhu-associated treatment strategies.

## Ethnopharmacology

### Basic Introduction

The use of Baizhu has been recorded in many ancient books and has been briefly introduced. In “Sheng Nong’s Herbal Classic,” there is no distinction between Baizhu and Cangzhu; the definitions of Baizhu may differ from that described in the present Chinese Pharmacopoeia (Chinese Pharmacopoeia Commission, 2020). In the Northern and Southern Dynasties, Tao Hongjing reported a preliminary distinction between the morphology and characteristics of the two herbs in Ben Cao Jing Ji Zhu (本草经集注) (Southern and Northern Dynasties, A.D. 480–498). No clear distinction existed in terms of properties, flavors, efficacy, and application, until explicit definition was provided in the Song Dynasty’s Ben Cao Yan Yi (本草衍义) (Northern Song Dynasty, A.D. 1116).

“Sheng Nong’s Herbal Classic” has reported that it has *bitterness* and *sweetness* in *flavor* and *warmness* in *nature*. It aids restoration of functions across the *spleen* and *stomach meridians*.

“Ming Yi Bie Lu” (名医别录) (A.D. 220–450) describes that it has *sweetness* in *flavor* and is nontoxic.

“Classified Materia Medica” (证类本草) (A.D. 960–1279) states that it possesses *bitterness* and *sweetness* in *flavor* and *warmness* in *nature* and is nontoxic. It is used to treat symptoms such as acidity, pain, numbness, and poor flexion and extension of joints and limbs. It can help remove excess heat, sweat, and aids digestion.

“Ben Cao Yan Yi” records that Baizhu is stout in appearance, is slightly brown in color, and has slight *bitterness*.

“Tang Ye Ben Cao” (汤液本草) (A.D. 1298) states that “Baizhu can enter the *Taiyang meridian of hand*, *Shaoyin meridian*, *foot Yangming meridian*, *Taiyin meridian*, *Queyin meridian.*”

“Ben Cao Meng Fu” (本草蒙筌) (A.D. 1368–1644) states that “Baizhu can enter the *heart*, *spleen*, *stomach meridians* and *Sanjiao meridian*.”

“Compendium of Materia Medica” (本草纲目) (A.D. 1552–1578) mentions that it is used in the treatment of chest and epigastric distension, suffocation, and discomfort. It is also effective against swelling of limbs, rashes, night sweats, postpartum vomiting, and so on.

“Ben Cao Bei Yao” (本草备要) (A.D. 1694) describes that it has *sweetness* and *bitterness* in *flavor* and *warmness* in *nature*. The *sweetness* and *warmness* can help strengthen the *warm innards*, while the *bitterness flavor* helps remove *dampness*.

The Chinese Pharmacopoeia states that it has *bitterness* and *sweetness in flavor* and *warmness* in *nature*. It can enter *spleen* and *stomach meridians*, invigorate the *spleen* and supplement *qi*, remove detrimental *dampness*, diuresis, and hidroschesis, and may be used as an antiabortion agent for successful gestation. Usage may be prescribed for treating *spleen* deficiency and eating disorders, abdominal distension and diarrhea, edema, spontaneous perspiration, acid reflux-associated abdominal pain, threatened abortion during the gestation period, or a minimal extent of vaginal bleeding.

### Traditional Applications

“Sheng Nong’s Herbal Classic” recorded that “Mainly for the treatment of *wind*-*cold*-*dampness* arthralgia, muscle necrosis, spasm, deep-rooted ulcer, excessive internal heat, and indigestion. Frying it into cakes and taking it for a long time can prolong life and satisfy hunger.”

“Ming Yi Bie Lu” states that it can be used to mainly treat vertigo, headache, eyes with excessive tears, excessive phlegm accumulation, and edema.

“Ben Cao Jing Ji Zhu” states that it can be used with Saposhnikoviae radix (the dried root of *Saposhnikovia divaricata* (Apiaceae)) and Sanguisorbae radix (the dried root of *Sanguisorba officinalis* (Rosacea)).

“Yao Xing Lun” (药性论) (A.D. 618–907) states that it is the main treatment approach for numbness, dysentery with bowel pain, and abdominal pain. It can stimulate appetite, eliminate phlegm, dispel cold and fever, cease diarrhea and antiaging, and eliminate wrinkle, treat edema, be used as antiemetic, and relieve abdominal pain.

“Xin Xiu Ben Cao” (新修本草) (A.D. 659) states that “Soaking the Baizhu in bitter wine, which can improve the curative effect of dry faces and dark skin.”

“Ri Hua Zi Ben Cao” (日华子本草) (A.D. 968–975) states that it can be used to cure *wind* disease, physical weakness, and abdominal distension and to treat soreness and weakness of waist and knees. It can also help resolve phlegm and diuresis, control nausea and vomiting, support weak muscles and bones, reduce intraperitoneal lumps with swelling and pain, cure fever, and help women with abdominal pain.

“Treatise on Febrile Diseases” (伤寒论) (A.D. 1065) (Song Dynasty edition) states that Zhang Zhongjing prepared Wuling San (Supplementary Table S2) for the treatment of dysuria, fear of cold, and wasting-thirst. Using Baizhu decoction (Supplementary Table S2) to cure constipation and sychnuria. Zhizhu decoction (Supplementary Table S2) is used to treat the symptom of “People with the disorder of viscera function and metabolism, have lumps in their *stomach*, such as disk-like lumps.”

“Yi Xue Qi Yuan” (医学启源) (A.D. 1127–1279) states that Baizhu contributes to the *dehumidification* and elimination of heat, strengthens *warm innards*, and augments vital energy. It also removes *dampness* in the *spleen* and *stomach*, removes *stomach* heat, strengthens the *spleen* and *stomach*, stimulates the appetite, neutralizes the toxicity in the *spleen* and *stomach*, aids production of body fluid, treats muscle heat and weakness of limbs, treats idleness and thirsty, and prevents miscarriage.

“Ben Cao Yan Yi Bu Yi” (本草衍义补遗) (A.D. 1347) states that it can reduce perspiration and resolve sputum.

“Compendium of Materia Medica” states that it is used to treat *spleen* and *stomach qi* deficiency, diarrhea, and so on.

“Synopsis of Golden Chamber the Pulse Syndrome of Pregnancy and Treatment” (金匮要略•妇人妊娠病脉证并治) (A.D. 1065) (Song Dynasty edition) states that it can be used in the form of Danggui powder (Supplementary Table S2) to treat blood deficiency, *dampness-*heat, and threatened abortion.

“Ben Cao Qiu Zhen” (本草求真) (A.D. 1644–1911) reports that it is known as “the best medicine for invigorating the *spleen* and for supplementing *qi*.”

“Yao Pin Hua Yi” (药品化义) (A.D. 1644) states that it should be avoided in case of *liver* depression and *qi* stagnation, choking sensation in chest and abdominal distension, asthma, hyperactive *stomach*, swelling, and fester.

“Ben Cao Cong Xin” (本草从新) (A.D. 1757) states that it helps dispel fatigue, treats muscle surface fever, and treats cellular masses that accumulate in the abdomen.

“Yi Xue Zhong Zhong Can Xi Lu” (医学衷中参西录) (A.D. 1909) states that Baizhu can be used to exert pulmonary effects, to regulate *liver* functions, to nourish the *heart*, and to restore functionality in the *kidneys* when used with other crude drugs.

“Ben Cao Zheng Yi” (本草正义) (1920) states that it can help improve disharmony of functions between the *spleen* and *stomach* and dredge of the *meridian*. Baizhu is rich in grease, and although it has *bitterness* in *flavor* and *warmness* in *nature*, it can also moisten the intestines and relieve constipation.

### Compound Medicine

As Baizhu demonstrates a variety of pharmacological effects, it can be suggested that Baizhu can be used as an important constituent of a variety of compound drugs in several ways. The main compounds are listed in Supplementary Table S2. We have presented a unified description of the names of TCMs enlisted in their Latin names to facilitate reading and to provide a reference for international readers (Rivera et al., 2014).

Baizhu has been used for nearly 2000 years in Chinese history, and the annual consumption of Baizhu in medical treatment increases exponentially. Through the types of compound drugs, it can be inferred that Baizhu is a common Chinese herbal component used in patent Chinese medicine and clinical compound medicine. It has gained prominence as one of the most indispensable ingredients used in health care products and the treatment of diseases.

## Phytochemistry

*A. macrocephala* and *A. lancea* are perennial herbaceous plants of Atractylodes in Compositae; hence, many chemical constituents have not only been isolated from Baizhu but a few have been derived from *A. lancea*. The isolated chemical constituents are listed in Supplementary Tables S3–S10.

### Terpenoids and Their Glycosides

Terpenoids, the main components of Baizhu volatile oils, demonstrate strong pharmacological activities. There are many types of sesquiterpenoids. The chief types are guaiane sesquiterpenes, cineole sesquiterpenes, spirosane sesquiterpenes, irimophenone, sesquiterpenes of vetiver, triterpenoids, steroids, and their glycosides. The main components and structures are listed in Supplementary Table S3.

### Alkynes and Their Glycosides

Atractylodin is the most frequently reported component in this category, and it is present in *A. chinensis* and *A. japonica*.

### Polyacetylenes of Diene-Diyne Types and Their Glycosides

Polyacetylene comprises conjugated structures with alternating single and double bonds. Owing to the nontorsional nature of the double bond, each unit of polyacetylene presents with both cis- and trans-structures. The main components and structures are listed in Supplementary Table S4.

### Triene-Diyne Types of Atractylodes Polyacetylenes and Their Glycosides

Molecules containing carbon-carbon double bonds and carbon-carbon triple bonds exhibit the same chemical properties as alkenes and alkynes and can undergo addition reactions and polycondensation reactions. The main components and structures are listed in Supplementary Table S5.

### Monoene-Diyne Types of Atractylodes Polyacetylenes and Their Glycosides

Most components are easily deactivated, and cyclized products are generated. They are considered as both biologically active sites and sources of their instability. The main components and structures are listed in Supplementary Table S6.

### Other Alkynes and Their Glycosides

There are few studies available on this topic in the literature, and only a few compounds have been identified, which can be enhanced and developed in the future. The main components and structures are listed in Supplementary Table S7.

### The Aromatic Glycosides and Acyl Sugar Compounds

Aromatic glycosides are also the important chemical components of Baizhu, and their main constituents are listed in Supplementary Table S8. However, acyl sugar compounds have been rarely mentioned, but a few have been isolated. The main components and structures are listed in Supplementary Table S9.

### The Other Compounds

In addition to the above-mentioned ingredients, Baizhu contains a few other ingredients. Other components of Baizhu warrant further study and classification. The main components and structures are listed in Supplementary Table S10.

## Pharmacological Effects

### Influence on Gastrointestinal Function

#### Promotion of Gastrointestinal Movement

Zhu et al. conducted a marker experiment and found that the aqueous decoction of Baizhu could promote the pigmentation of the Blue Dextran-2000 marker in stomach emptying and intestinal propulsion studies conducted in normal rats (Zhu et al., 2000). We further studied the mechanism by which Baizhu promoted intestinal peristalsis. Acetylcholine (Ach) and substance P (SP) are considered the most important excitatory neurotransmitters in the gastrointestinal tract. The effects of Baizhu on the distribution of Ach E- and SP-positive nerves in the gastrointestinal tract of rats were observed. The results showed that Baizhu could significantly increase the content of Ach E-positive nerve in the gastric antrum and intermuscular plexus of jejunum and SP-positive nerve in the intermuscular plexus of gastric antrum, submucosa of jejunum, and intermuscular plexus. There were significant differences. These results suggested that the increased distribution of Ach E- and SP-positive nerves in the gastrointestinal tract might play a role in the promoting effect of Baizhu (Zhu et al., 2001). This study provides a basis for studying the mechanism of intestinal peristalsis.

The study also explored the mechanism by which Baizhu activated functions of the stomach, invigorated the spleen, and regulated *qi* through intestinal peristalsis, to observe the effect of Baizhu on the contractile function of the digestive tract smooth muscle. It was found that adding a certain amount (0.05, 0.1, 0.2, and 0.5 g) of Baizhu water decoction (1 g/ml) to the equilibrium salt solution of Tyrode’s solution could significantly improve the contractile amplitude, contractile frequency, and antihypoxia ability of the jejunum of mice. Feeding a certain dose of Baizhu water decoction (Baizhu concentrations were 0.001 g/ml, 0.01 g/ml, and 0.1 g/ml) could significantly accelerate the movement of gastrointestinal contents in mice. These results indicate that Baizhu can enhance the contraction, amplitude, and frequency of small intestinal smooth muscle, improve its antihypoxia ability, accelerate the movement of gastrointestinal contents, and thus aid the function of invigorating the stomach and the spleen and may help regulate *qi* (Wu et al., 2005). This study shows that Baizhu can exert other related effects by promoting intestinal peristalsis, which broadens the scope of the research on Baizhu and motivates in-depth research on its unexplored effects.

#### Inhibition of Gastrointestinal Movement

Baizhu contains volatile oil and Atractylodes lactones. The study reported that Atractylodes lactones inhibited gastrointestinal peristalsis (Sun, 1999; Feng, 2001). Yu et al. found that it did not exert any effect on the intestinal muscle tension when the concentration of alcohol extract of bran-fried Baizhu was lower than 1.0 mg/ml. With an increase in the concentration, the inhibitory effect on the contractile force of the intestinal muscle was strengthened, and it demonstrated a complete inhibition effect (Yu et al., 2017). By conducting an experiment using Baizhu, it was found that the concentrations of 28 mmol/L and 56 mmol/L of Atractylodes lactone I, 4,15-epoxy hydroxy Atractylodes lactone, and Atractylodes lactone Ⅲ could help reduce the *in vitro* ileum contraction force in normal rats and could inhibit gastrointestinal movement. They are the active ingredients of Baizhu that inhibit gastrointestinal function (Zhang et al., 1999). The specific components of lactone with inhibitory effects on the gastrointestinal tract were described, and the dose was also tested. The experiment revealed that different methods of administration and dosages of Baizhu could also exert an inhibitory effect on the gastrointestinal tract.

#### Bidirectional Regulation

Jin et al. believed that Baizhu exerted a biaxially regulated effect on the gastrointestinal tract; one mechanism was that Baizhu could inhibit the function of the intestinal tract stimulated by acetylcholine, and the other mechanism was that Baizhu could stimulate the function of the intestinal tract stimulated by adrenaline (Feng, 2001). It was found that usage of the Buzhong Yiqi decoction (Supplementary Table S2) was effective for certain diseases exhibiting completely opposite major symptoms; accordingly, it could be speculated that it might exert a “bidirectional regulation” effect (Mao, 1994). All experimental findings showed that Baizhu demonstrated a certain bidirectional regulation effect, which also expanded the scope of the clinical application of Baizhu.

#### Repair of Gastrointestinal Mucosal Injury

Zhang et al. found that the 250 mg/L and 500 mg/L Baizhu effective groups (administered using the suspension prepared from the powdered form of the agent) could experience a significant reduction in the gastric mucosal injury caused by indomethacin exposure in mice and showed a significant difference compared with the blank group (Zhang and Chen, 2006). It was found that the sugar complex in Baizhu could upregulate the expression and distribution of villus proteins in IEC-6 cells. In turn, it could promote the differentiation of cells and repair of the gastrointestinal mucosa (Wang Z. et al, 2010). This study further explored the mechanism of gastric mucosal repair, which provided information for future research. The treatment with the methanol extract of Baizhu significantly increased the polyamine content of IEC-6 in intestinal epithelial cells. The cell membrane was hyperpolarized, the concentration of free calcium ions [Ca^2+^] _Cyt_ in the cytoplasm was increased, and the expression of the Kv1.1 channel gene was increased, which could collectively stimulate cell migration (Song et al., 2014). The emergence of these phenomena indicates the potential applicability of Baizhu in the treatment of intestinal mucosal injury and the difficulty in treating intestinal diseases. Further studies should be conducted in the future.

The results of the *in vivo* experiment showed that Atractylodes lactone Ⅰ could improve chronic atrophic gastritis (CAG) in model rats with the expression of heat shock protein 70 (HSP70), could reduce the content of interleukin-8 (IL-8), and could reduce the protein expression of nuclear transcription factor-κB (NF-*κ*B) and cyclooxygenase-2 (COX-2), thus reducing inflammation factors in gastric mucosal injury (Li et al., 2016b). The experimental findings elucidated the mechanism of repair of gastric mucosal injury from the perspective of exertion of effects on inflammatory factors, which further enriched the network of mechanisms responsible for the exhibition of this effect. *Atractylodes macrocephalae* polysaccharides (AMP) derived from Baizhu also increased the calcium ion level of IEC-6 in DFMO intestinal epithelial cells and promoted cell migration and E-cadherin expression (Wu et al., 2017). Atractylodes lactone Ⅲ also was found to boost the repair of gastric mucosal injury; the mechanism of action was dependent on the activation of tissue inhibitor of matrix metalloproteinases in gastric tissue, and the expression of matrix metalloproteinase-2 (MMP-2) and MMP-9 was inhibited to protect the gastric mucosa (Wang KT. et al, 2010). The experiments showed that Baizhu comprised many components that could aid the repair of the gastrointestinal mucosa, which proved the appreciable extent of repair for the gastrointestinal mucosa, and it was necessary to study its mechanism comprehensively to provide insights for future clinical trials. However, there are a few studies available on the dosage, and this paucity of information increases the difficulty of further extending the repair limit of the gastrointestinal mucous membrane, thus warranting studies for future research directions.

#### Modulation of Intestinal Microecology

It was found that Baizhu could promote the proliferation of beneficial bacteria such as *Lactobacillus* and *Bifidobacterium* in the intestinal flora (Yan W. L. et al, 2011) and could improve the balance of intestinal flora composition. During the investigation of the effects of AMP, it was discovered that AMP could affect the diversity of intestinal flora in anaerobic culture conditions, promote digestion by reducing sugars *via* the activity of intestinal bacteria, improve the symptoms of water-like stools caused by diarrhea, improve the similarity coefficient of the ERIC-PCR fingerprint of intestinal flora DNA, significantly regulate the structure of intestinal flora, and restore homeostasis. These results suggest that Baizhu can be used as an adjuvant to regulate intestinal flora composition and activity (Wang et al., 2014). The growth-promoting effects of AMP on *Bifidobacterium* and *Lactobacillus* were investigated. The results showed that AMP (2%) exerted an evident growth-promoting effect on *Bifidobacterium adolescentis*, *Bifidobacterium infantis*, *Bifidobacterium animalis*, and *Lactobacillus plantarum*. AMP (1%) presented with a good growth-promoting effect on *Lactobacillus acidophilus* (Liu et al., 2010). The experiment revealed that the influence on intestinal flora was also significantly related to the amount of polysaccharide added to Baizhu. Only the effects of AMP on the regulation of intestinal flora were described in the study, and there was a lack of information on other components, which also led to the lack of evidence for the clinical application of Baizhu. In future, we will study whether other components of Baizhu regulate intestinal flora composition and activity.

### Invigoration of the Spleen

Using enzymatic methods, the study found that Atractylodes lactone Ⅰ could enhance the role of saliva amylase activity in concentration of 0.8 mg/ml and that it might be considered as one of the effective components of Baizhu for invigorating and activating the spleen (Hao et al., 2006). The activity of salivary amylase, the absorption of nutrients in the small intestine of rabbits, and the function of the isolated intestine of rabbits were considered as indicators. Atractylodes lactone I strongly enhanced the absorption of salivary amylase activity, promoted bowel function, and amended intestinal function, confirming that Atractylodes lactone I was the active component of Baizhu responsible for tonifying the spleen (Li et al., 2006a). This validates the study conducted for the active parts and components of Baizhu that can be considered for spleen strengthening and provides a basis for the conduction of future research.

### Diuretic Effect

The Chinese Pharmacopoeia (the 2020 edition) states that Baizhu can eliminate *dampness* and diuresis. Chen et al. administered water decoction of Baizhu intravenously or *via* gavage to a variety of animals (equivalent to the administration of the crude drug at an intravenous dosage of 1.0 g/kg in rats, at an intragastric administration of 1.0 g/kg in rabbits, at an intravenous dosage of 0.05–0.25 g/kg in dogs, and at an intragastric administration of 1.0–3.0 g/kg) and reported that this can produce a significant and lasting diuretic effect. This illustrates the diuretic effect of Baizhu. Research also found that different doses and methods exerted different diuretic effects. After intravenous administration, Baizhu could increase urine volume significantly. Additionally, the effect of intragastric administration is relatively mild, but it is still more than that of the blank group. (Chen and Zhang, 1961). The mechanism underlying the diuretic effect was further studied.

Zheng observed and recorded the urine volume and other indices of patients with hepatic ascites after subjection to different doses of Baizhu and found that the use of a concentration of more than 60 g of Baizhu exerted evident diuretic effects, and it was positively correlated with the increase in dose (Zheng, 2002). The effective dose of Baizhu was explained in the experiment, and this provided the basis for reasonable and effective treatment. Li et al. studied the pathophysiology of ascites and found that the decoction of Baizhu could be used to control the peritoneal foramen *via* opening of the larger peritoneal foramen, thereby increasing the number of openings of the peritoneal foramen and increasing the average distribution density of peritoneal foramen to realize the effect of elimination of ascites and to indirectly produce a diuretic effect (Li et al., 1996b). These studies identified one of the reasons for the diuretic effect of Baizhu and provided a brief description of the dose, expanding the scope of its diuretic effect.

### Antidiuretic Effect

Baizhu exerts a diuretic effect; however, a few studies have shown that it also exerts an antidiuretic effect. While the water decoction of Baizhu was proven to exert a diuretic effect, it was also found that water decoction of Baizhu at medium and high doses (equivalent to 70 kg adult dose, 30 g/d at medium dose, and 60 g/d at a high dose of the crude drug) showed a certain antidiuretic effect in normal mice (Shi et al., 2007). Chen et al. studied the diuretic effect of Baizhu and its components in rats. It was also found that the high-dose water decoction of Baizhu (equivalent to crude drug 7.2 g/kg) and the high-dose volatile oil components of Baizhu (6.22 × 10^–2^ g/kg) demonstrated a certain antidiuretic effect in normal rats, as evidenced through the multimode combination method, and this was the first study to explore applications of the volatile oil of Baizhu in the generation of an antidiuretic effect (Chen et al., 2016). However, the specific mechanism of the antidiuretic effect has not been elucidated and merits further investigation. There are few reports available on the antidiuretic effect of Baizhu, and the specific chemical components in Baizhu that generate this effect have not been explained, thus warranting further research and discussion.

### Antitumor Effect

#### Inhibitory Effect on Solid Tumors

The antitumor effects of Baizhu volatile oil were studied. Baizhu volatile oil (at a concentration of 250 mg/kg) exerted a significant inhibitory effect on liver cancer H22 and sarcoma S180 cells in mice. However, the use of 125 mg/kg Baizhu volatile oil revealed a weak inhibitory effect on H22 and S180 of transplanted tumors in mice, indicating that Baizhu volatile oil demonstrated a significant inhibitory effect on H22 and S180 cells of transplanted tumors in mice; this effect was dose-dependent (Wang X. et al, 2002). The dosage used was stated to clarify the results further. The Baizhu volatile oil was separated into a large polarity portion (zbo-2) and a small polarity portion (zbo-1), and Atractylodes lactone Ⅰ and Atractylodes lactone Ⅲ were considered to compare the strength of the tumor suppression effect. The results showed that Atractylodes lactone Ⅰ (60 mg/kg), Atractylodes lactone Ⅲ (60 mg/kg), zbo-2 (200 mg/kg), and zbo-1 (small dose: 100 mg/kg and large dose: 200 mg/kg) in S180 mice could generate an antitumor effect. Zbo-1 showed the strongest antitumor effect at high doses, and the tumor inhibition rate reached a value of 24.74% (Shen et al., 2009). Although the experiment did not provide information on the specific components in the volatile oil responsible for the tumor inhibition effect, the volatile oil could be divided into two parts. Additionally, volatile oil with lesser polarity exerted a strong antitumor effect in large doses, a finding which would lay the foundation for the separation and identification of specific components in the future.

The inhibitory effect of different concentrations of Baizhu water decoction on the tumor mass of S180 sarcoma-bearing mice was observed. The results showed that each dose group of Baizhu could inhibit tumor growth; the medium dose group (crude drug, 0.5 g/ml) presented with the best effect (Zhu et al., 2006). In this study, the antitumor components of Baizhu were described, and the main antitumor mechanisms were explored, but the effects of specific targets on tumors could not be elucidated. The volatile oil of Baizhu presents with a wide range of antitumor effects, especially in hepatocellular carcinoma cells, as evidenced by findings of the activity test of five tumor cell lines, including SMMC7721, HepG2, A549, MCF-7, and HT29 (Lu, 2016). The results showed that the volatile oil of Baizhu exerted a good antitumor effect and demonstrated an inhibitory effect on many types of tumors. All experiments showed that Baizhu exerted a dose-related inhibitory effect on solid tumors. The relevant mechanism was also discussed, which provided information and laid the foundation for future research.

#### Indirect Effects on Tumor Cells

Upon activation *via* exposure to the volatile oil of Baizhu, the number and activity of phagocytic macrophages are markedly increased and their function is significantly enhanced. This contributes to the production of antibody-dependent cytotoxic effects mediated by macrophages and aid in the elicitation of responses against tumor cells (Ninomiya et al., 1991; Nemere, 1995; Guan et al., 2001). Studies have shown that Baizhu can help produce cytotoxic effects and can indirectly induce tumor inhibition. It was found that Baizhu volatile oil groups (volatile oil emulsion 0.025 ml/kg, volatile oil emulsion 0.05 ml/kg, and volatile oil emulsion 0.10 ml/kg) could significantly inhibit the lung metastasis rate of H22 liver cancer cells in a dose-dependent manner. It was also found that Baizhu volatile oil could significantly reduce the serum MMP-9 content in H22 liver cancer metastatic model mice, suggesting that Baizhu volatile oil might play an antitumor invasion and metastatic role by inhibiting the degradation of the extracellular matrix (Wang and Su, 2009). Other studies suggest that Baizhu may induce the apoptosis of HL-60 by increasing the content of reactive oxygen species (ROS), thus resulting in an antitumor effect (Huang HL. et al, 2005).

#### Inhibition of Cell Proliferation

Zhao et al. found that PG cells subjected to treatment with the volatile oil of Baizhu presented with a reduced ability of single-cell proliferation, and the adhesion and invasion abilities of PG cells subjected to treatment with volatile oil and AMP also decreased significantly (Zhao et al., 2005). Volatile oil and AMP inhibit the proliferation of tumor cells. The study found that using a concentration of 30 μg/ml of Atractylodes lactone I 12 h after treatment significantly induced apoptosis in the cell lines HL-60 and P-338. Atractylodes lactone I demonstrates antitumor effects; in contrast, Atractylodes lactone II and Atractylodes lactone III tumor suppression effect is relatively weak (Wang CC. et al, 2002). Thus, it can be speculated that Atractylodes lactone I is the main antitumor component of Baizhu. The antitumor effects of AMP were also studied. AMP has been found to inhibit the proliferation of tumor cells and induces their apoptosis, which leads to the arrest of tumor cell growth (Zhang et al., 2000; Cao et al., 2009). Ye et al. found that all eight sesquiterpenes isolated from Baizhu could inhibit the growth of B16 cancer cells (Yan Y. et al, 2011). Studies also suggest that the Atractylodes lactones Ⅰ, Ⅱ, and Ⅲ at doses of 200, 100, 50 μg/ml result in remarkable mice colon cancer cell proliferation inhibition effects. Among them, the effect of Atractylodes lactone II is the most ideal (Gao et al., 2013). It was also found that Atractylodes lactones I, II, and III at doses of 200, 100, and 50 μg/ml could inhibit ECA9706 esophageal carcinoma cell proliferation, and the effect of Atractylodes lactone II was remarkable (Gao et al., 2015). The two experiments show that Atractylodes lactone II exerts a stronger inhibitory effect on the proliferation of tumor cells.

The mechanism by which Baizhu inhibits tumor growth has been discussed. It was found that Atractylodes lactone I could downregulate the expression of Cyclin-Dependent Kinases 1 (CDK1) in ovarian cancer OVCAR cells SK-OV-3-3 through the Phosphatidylinositol-3-Kinase/Protein kinase B (PI3K/AKT) pathway and could trigger cell cycle arrest in the G2/M phase, thus inhibiting tumor cell proliferation (Long et al., 2017). This study indicated the mechanism by which Baizhu inhibited tumor growth and provided a foundation for future research. The proliferation of colorectal cancer Lovo cells was significantly inhibited when the concentration of Atractylodes lactone II reached a value of 150 mg/L. As the concentration increased, the cell survival rate decreased, indicating that Atractylodes lactone II could inhibit the growth and reproduction of colorectal cancer Lovo cells (Zhang et al., 2017). The inhibitory effects were dose-dependent. Zhu et al. found that Baizhu inhibited the tumor growth mechanism and demonstrated a certain inhibitory effect on the proliferation ability of gastric cancer SGC-7901SP cells *in vitro*, which was achieved by changing the cell cycle dynamics (Zhu et al., 2019). The study could not determine the specific ingredient in Baizhu that was responsible for this effect, and further research might be warranted. Many experiments have shown that the lactone class ingredients inhibit tumor proliferation; however, there are relatively few studies on the mechanism of tumor inhibition. The experimental design mainly included *in vivo* and *in vitro* experiments, with a lack of support based on clinical trial data. The mechanism of action can be further studied for clarification, and the reason for this action can be better considered in future clinical trials.

#### Promotion of Cell Apoptosis

Huang et al. showed that the methanol extract of Baizhu could induce apoptosis in tumor cells (Huang HL. et al, 2005). The results did not indicate the antitumor effect of a specific component present in the methanol extract of Baizhu. Atractylodes lactone II at a dose of 100 mg/L can exert a shear effect on the activities of Poly (ADP-ribose) polymerase 1 (PARP1) and caspase 3, and with an increase in drug concentration, the shear effect is enhanced, Atractylodes lactone Ⅱ can modulate the activity of PARP1, and caspase 3 protein expression promotes colorectal cancer Lovo cell apoptosis (Zhang et al., 2017). It was also found that its action was dose-dependent, and its mechanism was briefly explained. AMP significantly inhibited the proliferation of C6 cells in gliomas *via* fragmentation and apoptosis. It also induced apoptosis by triggering mitochondria-dependent pathways by destroying mitochondrial membrane potential (MMP) and by promoting the release of cytochrome C (Li et al., 2014). This suggests that polysaccharides from Baizhu also promote tumor cell apoptosis, but the lack of dosage description has hindered the application of this ingredient.

#### Other Antitumor Effects

Li et al. studied the anticarcinogenic activity of lactones in Baizhu. When lactones were applied to human cancer cell lines (MCF7, HepG2, Du145, Colon205, A549, and HL-60), the growth of cells in all cell lines was inhibited and the agents demonstrated good anticarcinogenic activity (Li et al., 2008). Many studies have also found that AMP exerts a strong antitumor effect, and AMP exhibits an inhibitory effect on colon cancer cells (Feng et al., 2019). It can enhance the immune function of rats in a lung cancer model, inhibit the proliferation of cancer cells, and induce apoptosis (Yin and Wang, 2019). The volatile oil of Baizhu was administered intraperitoneally at doses of 100 mg/kg and 50 mg/kg. It showed a strong inhibitory effect on the transplanted animal tumor Ehrlich’s ascites carcinoma (ECA). Simultaneously, it was found that a high dose (150 mg/kg) could prolong the lifespan of tumor-bearing mice by 197% (Zhang Z. et al, 2006). *In vitro* experiments showed that the volatile oil of Baizhu exerted a direct killing effect on ECA cells, and it also showed an inhibitory effect on ECA109 esophageal cancer cells (Wang and Wang, 2004). The reason for this effect was analyzed. All such studies indicate that Baizhu is a tumor-suppressive medicinal material, which proves its remarkable application potential. Moreover, a few studies have found that several specific chemical components of Baizhu exert good tumor suppressant effects, and this provides the basis and research directions for future research.

#### Antitumor Effect of Prescription

Shenling Baizhu powder (Supplementary Table S2) increased the expression levels of IL-2, Interferon-*γ* (IFN-*γ*), and tumor necrosis factor-*α* (TNF-*α*) cytokines in the peripheral blood of tumor-bearing mice. The curative effect of Shenling Baizhu powder combined with 5-FU (fluorouracil) was also studied. 5-FU alone can only promote the expression of IFN-*γ* and TNF-*α* in tumor-bearing mice but demonstrated no evident effect on IL-2. After subjection to combined treatment with 5-FU and Shenling Baizhu powder, it was observed that IL-2 content increased significantly, indicating that Shenling Baizhu powder might be used to directly stimulate Th1 cells to increase IL-2 secretion and might induce killer cells to secrete IFN-*γ* and TNF-*α* to increase immunity, thereby playing an antitumor role. These results indicate that the antitumor effect of Shenling Baizhu powder is related to the regulation of the immune function of the body, and the combined treatment with chemotherapy drugs may exert a synergistic antitumor effect by increasing the immune function of the body through high levels of IL-2, IFN-*γ*, and TNF-*α* cytokines (Huang et al., 2010). Qingshu Yiqi decoction (Supplementary Table S2) was used to treat mice with Lewis lung cancer. It was found that Qingshu Yiqi decoction could prevent weight loss in mice without increasing food intake. Additionally, it could reduce the secretion of IL-1*β*, IL-6, and TNF-*α* by mouse macrophages, thus producing an antitumor effect (Chou et al., 2012). This study illustrates the antitumor effect of several compounds of Baizhu and provides a comprehensive basis for the antitumor effect of Baizhu.

#### Anti-Inflammatory Effects

Baizhu also demonstrates anti-inflammatory effects. Huang et al. investigated the anti-inflammatory effect of external application of Baizhu by estimating the serum TNF-*α* content of inflammatory mice after subjection to a water decoction of Baizhu *via* an external application. They found that external application of water decoction of Baizhu reduced the serum TNF-*α* content in inflammation-presenting mice (Huang Y. Y. et al, 2005). The positive control group was used in the experiment, which could exhibit the anti-inflammatory effect of the external application of Baizhu and could help expand its application range. Li et al. experimentally explored the anti-inflammatory effect of the main active ingredient in Baizhu (Atractylodes lactone I). The results showed that the high-dose group of Atractylodes lactone Ⅰ (300 mg/kg) presented with a significant reduction in inflammatory swelling of the auricle induced by xylene exposure and with an increase in capillary permeability induced by acetic acid exposure in the abdominal cavity of mice. The mechanism of anti-inflammatory production was also investigated, and the results showed that Atractylodes lactone I presented with the same site of action as paclitaxel (model molecule) in the white cell membrane chromatogram model. Moreover, it could be antagonized *via* exposure to lipopolysaccharides (LPS), and its target might be the Toll-like receptor 4 (TLR4) receptor. Therefore, it is speculated that it may produce anti-inflammatory effects by antagonizing the activity of TLR4 (Li and He, 2006). This experimental finding suggested the mechanism of the anti-inflammatory effect, which would provide information for future research. The experiment also explained the specific components of Baizhu, which could exert anti-inflammatory effects.

The compositions with anti-inflammatory activity in Baizhu were studied. The results showed that the five chemical components isolated from Baizhu could significantly inhibit mouse ear swelling caused by dimethylbenzene exposure, indicating that these components could exert certain anti-inflammatory effects on acute inflammation in mice. Among them, 12-senecioyl-14-acetyl-2E, 8E, 10E-triene-4, and 6-diyne-1-alcohol were the first compounds reported to demonstrate anti-inflammatory effects (Dong et al., 2008). The discovery of these components provides information for studying the pharmacological action of specific components in Baizhu. To explore the anti-inflammatory mechanism of Baizhu, the effects of Atractylodes lactone I and Atractylodes lactone Ⅲ in terms of LPS-induced nitric oxide (NO) generation and TNF-*α* expression were explored. The results showed that Atractylodes lactone Ⅰ and Atractylodes lactone Ⅲ significantly reduced the production of TNF-*α* and NO and inhibited the activity of TNF-*α* mRNA and inducible nitric oxide synthase, and it was found that the Atractylodes lactone I-mediated limit of inhibition was stronger than the Atractylodes lactone Ⅲ-mediated limit (Li et al., 2007a). This provides insights into a new approach for the treatment of inflammation, and new drugs for the treatment of inflammation can be developed based on this finding in the future; additionally, the anti-inflammatory effects of several components in Baizhu have been compared. Through white cell membrane chromatography (WBCM-C) screening of anti-inflammatory ingredients, the results indicated that WBCM-C anti-inflammatory activity of Atractylodes lactone I was similar to that of dexamethasone and demonstrated marked effects in an acute and chronic inflammation model in rats. Oral Atractylodes lactone I also exerts anti-inflammatory effects (Li et al., 2007b). Studies also suggest that Atractylodes lactone I can limit the levels of inflammatory cytokines (TNF-*α*, IL-6, and IL-1*β*), alanine transaminase (ALT), aspartate transaminase (AST), creatinine (CRE), and blood urea nitrogen (BUN), can improve sepsis syndrome, and can improve kidney function (Wang A. et al, 2016). Atractylodes lactone I inhibited the proliferation of vascular smooth muscle cells (VSMCs) induced by oxidized modified low-density lipoprotein (OXLDL). Migration contributes to antiatherosclerosis by responding to the expression of monocyte chemoattractant protein-1 (MCP-1) and by downregulating the expression of effective inflammatory mediators of the vascular inflammatory response (Li et al., 2017). This illustrates that Atractylodes lactone Ⅰ possesses significant anti-inflammatory activity, and the mechanism of its anti-inflammatory action has been briefly described.

### Improvement in Nervous System Diseases

#### Improvement in Cerebral Ischemia Injury

In clinical treatment, AMP was found to alleviate brain edema after focal cerebral ischemia and reperfusion and could reduce nerve cell damage, could improve neurological function deficits, and could confer protection to nerves. It was also found that it could reduce the activity of inducible nitric oxide synthase, could improve the activity of superoxide dismutase, could reduce the content of malondialdehyde, and significantly could improve the reperfusion injury caused by inflammation (Dong, 2015). Studies have confirmed the relevant effects of Baizhu and have demonstrated that Baizhu can improve nervous system disease. A rat model of focal cerebral ischemia/reperfusion was established. The recovery of neurological function was evaluated based on the changes in muscle strength and Longa score. The activities of superoxide dismutase (SOD), glutathione (GSH), and catalase (CAT), and the content of malondialdehyde (MDA) in the rat brain were determined. The results showed that Baizhu could significantly improve the neurological behavior of rats after cerebral ischemia/reperfusion, could increase the activities of SOD, GSH, and CAT in brain tissue, and could reduce the content of MDA (Gao, 2017). These results indicated that Baizhu could reduce cerebral ischemia/reperfusion in rats, and the changes in the detection indices could be attributed to the antioxidant mechanism.

#### Improvement in Senile Dementia

To observe the effect of biatractylenolide on learning and memory, the change in cholinesterase content in brain tissue of rats with Alzheimer’s disease induced by β-amyloid was analyzed. The results showed that biatractylenolide could effectively improve the learning and memory ability of rats with dementia, and to a certain extent, it could alleviate the mental impairment in rats with dementia (Feng et al., 2009). In this study, the effects of Baizhu on the relief of dementia symptoms were explained. A positive control group was used in the experiment, and thus the results were more reliable; results showed that the administration of a high dose (1.0 mg/kg/d) of biatractylenolide was comparatively effective. Authors also used water mazes to confirm learning and training results, and the acetylcholinesterase content in brain tissue homogenate was measured by colorimetry to observe the effect of biatractylenolide on the model mice with memory impairment. The results showed that biatractylenolide could effectively reduce the activity of cholinesterase in the brain and could improve the memory ability of mice with dementia induced by aluminum trichloride exposure (Liu and Liao, 2006). The results showed that Baizhu exerted a therapeutic effect in dementia models caused by various factors.

#### Improvement in Memory

Baizhu has also been shown to improve memory. A brain aging mouse model was established by continuous subcutaneous injection of d-galactose. The effects of memory ability and gray type I synaptic structural parameters in the CA3 region of the brain in aging mice were evaluated. The results indicated that the escape latency of the low-, medium-, and high-dose groups of Baizhu was significantly shorter than that of the brain aging group. Compared with the control group, the synaptic interface curvature and postsynaptic density thickness of the gray type I synaptic structure in the hippocampal CA3 region of mice in the brain aging group were significantly decreased, and the synaptic gap width was significantly increased. However, the synaptic interface curvature, synaptic gap width, and postsynaptic dense thickness of mice in the Baizhu group were similar to those in the control group (Gao and Yu, 2016a). The results showed that Baizhu could improve gray type I synaptic structure in the hippocampal CA3 region and the learning and memory ability of mice induced by brain aging.

The passive avoidance test was also used to explore the learning and memory abilities of the mice. The expression levels of synaptophysin (SYN), protein kinase C (PKC), and cyclic adenosine monophosphate (CREB) reaction element-binding proteins in the hippocampus were detected. The results showed that Baizhu significantly improved the learning and memory ability of the mice with brain aging, and the mechanism of Baizhu might be related to the upregulation of SYN, PKC, and CREB expression and the influence of synaptic plasticity (Gao and Yu, 2016b).

#### Antidepressant Effect

Experimental studies have also shown that Baizhu also exerts antidepressant effects. Liu et al. found that Atractylodes lactone III inhibited glutamate-induced neuronal apoptosis in a concentration-dependent manner (Liu et al., 2014). Atractylodes lactone I usage reduced IL-1*β* production by inhibiting Nucleotide-binding oligomerization domain, leucine-rich repeat, and pyrin domain-containing 3 (NLRP3) inflammasome activation and exerted an antidepressant effect in a mouse model of chronic unpredictable mild stress- (CUMS-) induced depression (Gao et al., 2018). These results suggest that Baizhu may exert antidepressant effects.

#### Improvement in Other Aspects

Studies have found that Baizhu plays the role of dual-directional regulation of the autonomic nervous system. By introducing adjustments in the autonomic nervous system, Baizhu can be used to treat related diseases similar to digestive tract dysfunction in patients with spleen deficiency and can help achieve the purpose of invigorating the spleen (Du and Nie, 2004). Therefore, it can be inferred that the improvement of nervous system-related diseases by Baizhu is one of the reasons for its application in invigorating the spleen. Experimental *in vitro* cultivation of nerve cells showed that the AMP level shown by the control in the range of 0.025 g/L-0.05 g/L could significantly improve nerve cell growth inhibition caused by hypoxia and could significantly improve early apoptosis of nerve cells, suggesting that AMP could reduce the expression of apoptosis genes and could increase the levels of antiapoptotic proteins, along with suppression of the effect of nerve cell apoptosis (Hu et al., 2014). AMP may also help reduce the degree of secondary brain edema after traumatic brain injury by downregulating the expression of nitric oxide synthase in the wound area (Wang G. W. et al, 2009; Shi et al., 2014), thus leading to an improvement in neurological diseases. Hu et al. also found that AMP (polysaccharide mass fraction, 95%) at a range of 0.025–0.1 g/L could inhibit the hypoxic apoptosis of nerve cells in the cerebral cortex of SD fetal rats (Hu et al., 2014), indicating that Baizhu could also improve neurological diseases by affecting brain cell apoptosis.

### Effects on the Immune System

Sun et al. found that AMP could stimulate the production of specific IgG antibodies and nonspecific cross antibodies in mice and speculated that AMP might be a pan-specific broad-spectrum immunomodulator (Sun et al., 2008); however, the study lacked an explanation on dosage use. Studies further confirmed that exposure to polysaccharide antigens could stimulate the production of specific IgG antibodies in the body and could also stimulate the production of nonspecific IgG antibodies, i.e., cross antibodies, to a certain extent, but did not stimulate the production of pathological antibodies (Sun et al., 2011). Moreover, it was suggested that the higher the degree of purification of AMP, the stronger the promotion effect on the proliferation of splenic lymphocytes in mice. The protein in crude AMP demonstrated no effect on the proliferation of splenic lymphocytes in mice (Guo et al., 2012). The degree of purification of polysaccharides has been illustrated in this study, which provides a basis for future research.

Recent studies have shown that polysaccharides from Baizhu can not only significantly and positively promote the number and function of white blood cells in immunosuppressed mice but also significantly improve the function of lymphocytes in normal mice (Xiang et al., 2020). However, the specific mechanism of Baizhu’s influence on the immune system remains to be further explored and studied, and further experiments are warranted to optimize the dosage. More *in vivo* and *in vitro* experimental data are needed to support the clinical application of Baizhu.

### Maintenance of Uterine Smooth Muscle Functions

The Chinese Pharmacopoeia (2020 edition) states that Baizhu exerts an impact on fetal safety; hence, many researchers have conducted several experiments to confirm this effect. Baizhu was found to trigger excitation in uterine smooth muscle cells in late pregnancy and could excite the potassium channel current (BKCA) of uterine smooth muscle cells subjected to treatment with IL-6, which helped maintain the membrane potential and resting state of uterine smooth muscle during pregnancy to prevent premature birth (Zhang et al., 2009). This provides a basis for the development of TCM for the treatment of premature birth. It was found that doses of 4 g/L and 8 g/L Baizhu inhibited normal uterine smooth muscle contractile activity; however, the doses did not exert a dose-dependent effect (Zhang et al., 2008). It was found that the antiabortion effect of Baizhu was associated with the regulation of uterine smooth muscle, a finding which contributed significantly to the development of new drugs.

### Antioxidant and Antiaging Effects

AMP has been found to exert antiaging effects. AMP can significantly increase the activities of SOD and GSH-Px, reduce the content of MDA, and reduce DNA damage in nerve cells of senile rats, implying that AMP demonstrates certain antiaging effects (Ma et al., 2006). A dose of 0.2 g/ml of bran-fried Baizhu, soil-fried Baizhu, raw Baizhu, and vinegar-containing Baizhu decoction (0.2 ml/20 g) was used for treating subacute aging in a mouse model established by exposure to by d-galactose lavage for 6 weeks, and the vinegar water system of Baizhu decoction could help reduce the serum MDA levels in aging mice, liver tissue lactoferrin (Lf) levels, serum SOD levels, and CAT activity. It is suggested that Baizhu and its processed products (except vinegar-prepared products) exert a certain antiaging effect (Song and Gu, 2007). It could be speculated that the processing did not affect the activity of AMP, but the antiaging effect continued to be observed. We found that, after the injection of AMP, the activities of total antioxidant capacity (T-AOC) and SOD in the serum and brain tissues of mice were enhanced, as evidenced using the classic animal model, and the effects could improve the antioxidant capacity of the body. A possible mechanism is that AMP can reduce the concentration of MAO in brain tissues, reduce the generation and accumulation of lipofuscin (Lipo), and thus lead to the achievement of antioxidant and antiaging effects (Shi et al., 2014). The mechanism of antioxidation was studied, and it was found that the antioxidation effect was related to the antiaging effect, which provided a basis for clinical application in the future.

### Hypoglycemic Effect

An alloxan diabetic rat model was established, and the results showed that AMP-B could increase the thymus weight of alloxan diabetic rats and pancreas weight index, could inhibit diabetic rat pancreas atrophy, and could explain that Baizhu sugar compounds might decrease alloxan effects on islet β-cell damage or might improve impaired β-cell function (Shan and Tian, 2003). The mechanism of the hypoglycemic effect was explained in the experiment, but only the alloxan diabetes model was studied. Studies on other models were lacking. Experimental studies have revealed that AMP can effectively reduce fasting blood glucose and plasma insulin levels in type 2 diabetic mice and can help improve glucose tolerance in diabetic mice (Li Y. et al, 2015). It is believed that the possible mechanism is the achievement of a state of hypoglycemia by increasing insulin sensitivity, which also provides a basis for the application of Baizhu in clinical practice. Atractylodes lactone Ⅰ and Atractylodes lactone Ⅱ influence glucose uptake in skeletal muscle C_2_C_12_ cells in mice, and it has been found that both significantly increase glucosetransporters-4 (GLUT-4) protein levels and promote GLUT4 translocation to the plasma membrane. Further studies have shown that it improves TNF-*α*-induced insulin resistance in C_2_C_12_ skeletal muscle cells and exerts a hypoglycemic effect (Chao et al., 2016). These studies have explained the mechanism of the hypoglycemic effect, but more mechanisms should be studied. These data provide a certain pharmacological basis for the development and utilization of Baizhu in hypoglycemic aspects and may help expand the research scope of new drugs for hypoglycemia, with provision of a relevant basis for clinical application.

### Analgesic Effect

Baizhu has been found to exert analgesic effects. A few studies have investigated the analgesic effect of the ethanol extract of Baizhu, in which high and medium doses of ethanol extract of Baizhu can significantly increase the hot plate pain threshold of mice and can help reduce the number of writhing reactions caused *via* intraperitoneal injection of acetic acid; however, no effect is observed in the low-dose group. The inhibition rate of licking foot in mice after subjection to different doses of Baizhu was less than 50%, indicating that Baizhu alcohol extract was not a central analgesic (Xu et al., 2014). These experiments explained the reason for its occurrence. There are few studies available on the analgesic effects of Baizhu; hence, the main analgesic mechanism of Baizhu has not been explained and should be further explored.

### Other Effects

Baizhu demonstrates other roles, such as antibacterial effect (Yang et al., 2002). A study indicated a protective effect of Baizhu in hepatic IRI in rats (Peng, 2009; Jin et al., 2011). Another indicated aromatase inhibition (Jiang et al., 2011) and enhancement of the contractility of the myocardium (Pu et al., 2000; Ma and Mei, 2007). Findings of a study indicated that Baizhu exerted a liver protection effect (Kiso et al., 1983; Hwang et al., 1996). It can also reduce hematic fat content (Peng et al., 2011). It improved acute kidney injury (Li Q. C. et al, 2015) and led to the inhibition of adipogenesis (Kim et al., 2011). Baizhu also demonstrated a hypotensive effect (Li and Feng, 1997) and was found to relieve certain aspects of bone disease (Li et al., 2012). The main pharmacological effects of Baizhu are presented in Supplementary Table S11.

## Application

The Baizhu water decoction (based on the use of 60 g Baizhu) exerts a marked effect on the treatment of colonic slow transit constipation (STC), with moistening of the intestines, and has shown remarkable effects in ameliorating the deficiency of *qi* and *yin* (Ding et al., 2005). The dosage of Baizhu should be administered according to the degree of *spleen* deficiency and constipation. Generally, the dosage is 15–30 g, while 30–60 g can be used for moderate treatment (Wang and Fan, 2005). Additionally, the effect of a large dose of Baizhu (35–60 g) was gentlest and the purgative effect was more evident (Liu, 2009). STC is functional constipation clinically characterized by reduced colonic transport movement and prolonged colonic transit time. It is a common type of chronic constipation. The following are the mechanisms of Baizhu involved in the treatment of STC: 1) promotion of gastrointestinal myotonia, 2) establishment of a balance between the number and distribution of Cajal interstitial cells (ICC), 3) regulation of the enteric nervous system (ENS), and 4) maintenance of intestinal flora composition (Zhao et al., 2017b). Liu et al. used different doses of Baizhu to treat postoperative constipation, and the results showed that large doses of Baizhu presented with significant cathartic effect, gentlest efficacy, and high safety (Tang, 2007). Baizhu Shaoyao powder (Supplementary Table S2) containing Baizhu as the dominant drug can be used for the treatment of painful diarrhea, intestinal inflammation (Xu et al., 2018), and irritable bowel syndrome (IBS), a condition which is dominated by diarrhea (Zheng et al., 2015). Xiangsha Liujunzi decoction (Supplementary Table S2), in which Baizhu is the dominant drug, demonstrates a certain effect of invigorating the *spleen* and exerts a certain effect on functional dyspepsia, especially postprandial discomfort syndrome (Wang F.-Y. et al, 2016). Baizhu Shanzha decoction (Baizhu) and Crataegi Fructus (the dry mature fruit of *Crataegus pinnatifida* Bunge (Rosaceae)) are used to treat ulcerative functional dyspepsia (Wang, 2007). Baizhu Shaoyao powder was added to treat ulcerative colitis (Li et al., 2009). Qiwei Baizhu powder (Supplementary Table S2) was resistant to the elimination of pathogenic factors. Baizhu powder can be used to treat diarrhea in children (Zhang and Zhang, 2007). Shenling Baizhu powder is used to treat anorexia, peptic ulcers, gastrointestinal dysfunction, chronic enteritis, and simple dyspepsia after the surgical operation of malignant tumors of the digestive system (Hao and Hao, 2004; Ge and Wu, 2006; Xu, 2006; Sun, 2008; Peng, 2009).

Banxia Baizhu Tianma decoction (Supplementary Table S2), with Baizhu as the dominant drug, is widely used in the treatment of vertigo caused by vertebrobasilar artery insufficiency, with definite curative effects (Guo et al., 2017). It also exerts certain effects on hypertension (Tan et al., 2018) and hyperlipidemia (Cai et al., 2018). Animal experiments have confirmed that the Danggui Baizhu decoction (Supplementary Table S2) can reduce blood lipids and prevent obesity (Zhao et al., 2018). The Banxia Baizhu Tianma decoction can be used to treat vertigo, Meniere’s disease, headache, cerebral infarction, concussion, transient ischemic attack, and essential hypertension (Liu and Zhong, 2006; Liu et al., 2007; Li, 2009; Ning and Zhong, 2009; Zhang and Hao, 2009; Yan W. L. et al, 2011; Li et al., 2020).

Eighty-eight hypertension patients were randomly divided into two groups, namely, control group patients with subjection to oral captopril and observation group patients subjected to the use of Banxia Baizhu Tianma decoction. After the conduction of tests, researchers found that, in the observation group, plasma angiotensin Ⅱ, renin, and aldosterone contents were lower than those of the control group and that the antihypertensive effect was better than that of the control group (Miao et al., 2017).

Yupingfeng powder (Supplementary Table S2), with Baizhu as its main drug, can be used to treat diseases related to the immune system, such as cold (Du et al., 2015), chronic bronchitis, allergic rhinitis, and asthma (Nikles et al., 2017), by regulating the inflammatory response and phagocytosis of phagocytes (Du et al., 2014). The investigation found that Shenling Baizhu powder combined with antibiotics could be considered to treat acute otitis media and reported that such a combined treatment approach was more effective than that based on the use of antibiotics alone (Son et al., 2017).

Qushi Huayu decoction with AMP as the main active ingredient can significantly reduce the liver triglyceride content and exerts a good therapeutic effect on nonalcoholic fatty liver disease (Meng et al., 2016). Studies conducted on Linggui Zhugan decoction (Supplementary Table S2) showed that the prescriptions could significantly improve liver fat content, and Baizhu played a role in invigorating the *spleen* and dryness of the *spleen* (Liu et al., 2013a). In the Yinchen Zhufu decoction (Supplementary Table S2), Baizhu is an important crude drug that exerts a good protective effect against alpha-naphthylisothiocyanate-induced acute cholestatic liver injury in mice (Wang GF. et al, 2020). The traditional Korean “*liver clearing*” formula, Chunggan extract (CGX), is a modification of traditional herbal medicine that has been used for treating patients presenting with various liver disorders since 2001 (Kim et al., 2014). It consists of 13 Chinese herbal medicines (Supplementary Table S2) such as Baizhu and is used for the treatment of chronic toxic hepatitis, liver fibrosis, cirrhosis, and alcoholic liver disease (Kim et al., 2013). Treatment of cirrhotic ascites with Qiwei Baizhu powder (Li, 2005) and Shenling Baizhu powder can also help treat cirrhosis (Wang, 2008).

Baizhu powder is used to treat diabetes mellitus (Zhang J. D. et al, 2006). Shenling Baizhu powder is used to treat edema, obesity, chronic eczema in children, uremia combined with malnutrition, and middle-and late-period ankylosing spondylitis (Dai and Wang, 2007; Lu, 2006; Wang, 2009; Yu and Qiu, 2007; Zuo et al., 2007). The treatment of 60 female patients with chronic pelvic inflammation and blood stasis syndrome by using Lichong decoction (Supplementary Table S2) as the main drug of Baizhu combined with moxibustion was remarkably effective (Chen et al., 2015). A total of 106 patients with insomnia were randomly divided into the following two groups: the observation group was subjected to treatment with Buzhong Yiqi decoction, and the control group was provided with oryzanol. The results showed that the improvement of insomnia in the observation group was better than that in the control group (Wang, 2015).

Seventy-two patients with cerebral vascular dementia and stasis were randomly divided into a control group and an observation group. Both groups received conventional treatment. The control group was additionally subjected to treatment with nimodipine, and the observation group was additionally subjected to treatment with Banxia Baizhu Tianma decoction combined with moxibustion. The results showed that cognitive function improved more in the observation group (Hao, 2017). The main applications of Baizhu are presented in Supplementary Table S12.

With such a long history of usage and varied applications, Baizhu and its formulas demonstrate a good curative effect on diseases. TCMs, whether provided as a single herb or as a prescription with multiple herbs, emphasize that it plays multiple roles in the treatment of diseases. It is provided as a multicomponent adjustment to multiple targets *via* multiple pathways with the purpose of curing diseases (Heinrich et al., 2020).

## Quality Control

The Chinese Pharmacopoeia (the 2020 edition) recommends the use of thin-layer chromatography (TLC) for the qualitative identification of Baizhu. There is no provision for quantitative identification of Baizhu; hence, few researchers use different methods to determine the content of different chemical components in Baizhu. By performing reversed-phase high-performance liquid chromatography (RP- HPLC) and HPLC analysis of Atractylodes lactones Ⅰ and Ⅲ, 25 different Baizhu were measured for both. The sample recovery rate was markedly high, and this method was simple, sensitive, and reliable and could be used as a quantitative method for quality control of Baizhu (Li et al., 2001). AMP (Pingjiang, Zhejiang, and Huaihua) was extracted *via* reflux boiling with water and reflux ethanol. The contents of water-soluble and reductive sugars were determined by spectrophotometry at a 493.6 nm wavelength. The average recovery was 102.5%, and the RSD was 2%. This method is simple, rapid, and accurate and can be used for the identification and quality control of Baizhu derived from different producing areas (Qiu et al., 2005). Li also used RP- HPLC to determine the content of Atractylodes lactone II, and reported good extraction efficiency, and further showed that this method could be used for quality control of the Baizhu quantitative method (Li et al., 2005). Presently, the content determination of Baizhu is mainly conducted *via* HPLC. Methanol-based ultrasonic treatment for 30 min was used to prepare the test solution. The mobile phase system of methanol-water and acetonitrile-water (phosphoric acid) was used to determine the content of Baizhu by the HPLC-ultraviolet (UV)/DAD method. Using the gradient elution HPLC-DAD method and determination of Atractylodes lactones I, II, and III, two signal record chromatograph charts were created. They included Atractylodes lactone I and III detection at a wavelength of 220 nm and Atractylodes lactone Ⅱ at a wavelength of 276 nm for the content determination method, to ensure quality control of the raw product and to limit the quality of processed products to provide the reference (Duan et al., 2009). Most studies focus on the quality control of Atractylodes lactone components, and atractylon is also one of the indicators of quality control. However, because atractylon is unstable and difficult to detect, most of the studies are the detection of Atractylodes lactones Ⅰ, Ⅱ, and Ⅲ. The microtubule liquid phase method was used to determine the content of Baizhu samples from different producing areas. At the same time, the contents of atractylon and Atractylodes lactones I and III were determined, and data were then applied to the limit of medicinal material quality evaluation and medicinal germplasm resources and cultivation and for the selection of excellent provenance. They showed positive effects (Shou et al., 2008). The components extracted *via* headspace solid-phase microextraction (HS-SPME) and steam distillation (SD) were analyzed *via* gas chromatography-mass spectrometry (GC-MS). The experimental results showed that the extracted components of the two methods were the same, but SPME was simpler, faster, and more suitable for the extraction and semiquantitative analysis of volatile components in Baizhu. Owing to this feature, the HS-SPME-GC-MS method was established for the separation and identification of volatile components in Baizhu (Guo et al., 2007). The optimization of the HPLC-DAD Baizhu index component content determination method led to the determination of four index components of Atractylodes lactone I, Atractylodes lactone II, Atractylodes lactone Ⅲ, and atractylon. The method demonstrates the advantages of simplicity, efficiency, accuracy, and stability (Wang L. M. et al, 2020).

The fingerprint of Baizhu (Youyang County, Chongqing) was established using HPLC. The chromatogram analysis and data management software were used to automatically match the peaks, and 11 common peaks were identified. The chromatogram of sample 2 was considered as the template, and the similarity of the fingerprint was calculated using the correlation coefficient method, including the angle cosine method. The similarity of the two methods was greater than 93%, which indicated that the established fingerprint was stable and reproducible and could be used as the quality control standard for Baizhu. Thirteen batches of Baizhu samples from different production areas were determined, and twenty-four common peaks were identified. The similarity and other related parameters were calculated using the “Chinese medicine fingerprint computer-aided similarity software” and cluster analysis methods, and the common pattern of the Baizhu finger pattern was established. The similarity between each sample was above 0.9, and the similarity with the common pattern was 0.95, which provided a scientific basis for better control of the internal quality of Baizhu (Li Q. H. et al, 2007). HPLC chromatographic analysis of 13 batches of Baizhu samples was performed. All chromatographic fingerprints were compared using the “Chinese Medicine Chromatographic Fingerprint Similarity Evaluation System 2004 Edition” recommended by the Pharmacopoeia Committee, and 12 peaks were identified as common peaks. RSDs of the relative peak area and relative retention time of common peaks ranged from 16.61 to 65.04% and 0.02–0.03%, respectively (Ye et al., 2009). Wan et al. used ultraperformance liquid chromatography coupled with triple-quadrupole tandem mass spectrometry (UPLC-MS/MS) to identify and validate the Atractylodes lactone I in Baizhu, which is one of the main components of the Lichong decoction which was positively absorbed in different intestinal segments. Atractylodes lactone I could be used as an indicator component of the Lichong decoction. (Wan et al., 2021).

Baizhu is a commonly used TCM. It has been widely used since ancient times. There are also many compounds with Baizhu as the main medicinal material used in clinical practice, which present with high development and application value. Quality control of Baizhu is necessary. Many studies have conducted experiments, and liquid chromatography is the most common method reported; however, other methods such as GC-MS, microtubule liquid method, and serum pharmacochemistry can be used for quality control of Baizhu. There are few studies on the fingerprint of Baizhu, and few studies have been conducted on the basic pharmacological substances of Baizhu. Therefore, it is impossible to use pharmacological substances to truly guide the quality control of medicinal materials, decoction pieces, and preparations; hence, further researches are needed. Nevertheless, a thorough study of the functional characteristics and mechanisms of biomacromolecules is helpful for a thorough understanding of the medicinal properties and health care efficacy of TCM. Most studies were conducted on the constituents of Baizhu and focused on the constituents of volatile oil and lactones. Compounds with similar structures are likely to exert the same pharmacological effects on the same target, and this phenomenon may provide experimental evidence for the concept of “effective forms” and the hypothesis of “additive effect” (Zhang et al., 2021). The main quality control methods for Baizhu are shown in Supplementary Table S13.

## Processing

The purpose of most TCM processing methods is to reduce stimulation, moderate medicinal properties, and increase the curative effect. Baizhu is no exception. Presently, the main processing methods of Baizhu are as follows (Du & Nie, 2004):

1) Steamed Baizhu: place the clean Baizhu in the steamer; add the appropriate amount of rice to Baizhu; cover with gentle fire for 8 h until the exterior of the Baizhu is black and the inside is brown. When the rice is thoroughly cooked, take Baizhu out and spread out.
2) Stir-fried Baizhu: heat a wok and sprinkle the bran evenly. When cooking of the wheat bran begins with the gradual generation of smoke, pour the Baizhu decoction pieces into the wok, and stir quickly until the drug is yellow on the surface and yellowish-brown on the inside. Remove when an aroma is produced. This method can alleviate dryness, invigorate the *spleen*, and replenish *qi* of the effect of Baizhu.
3) Soil-fried Baizhu: put old soil in a wok over medium heat and stir fry the soil. Then, add the Baizhu decoction pieces and stir rapidly until Baizhu is colored and fragrant. This method is excellent for achieving Baizhu for invigorating the *spleen* and for cessation of diarrhea, and such a product is mainly used for treating *spleen* deficiency and diarrhea.
4) Soaked Baizhu with rice swill: soak clean Baizhu decoction pieces and bleach in water for 2 days. Then, remove the soy-colored water, and bleach the rice water for 1 day to obtain white coloration. Remove and sun-dry the product obtained therein. This method increases the product’s efficacy in invigorating the *spleen* and *stomach.*
5) Focal Baizhu: put clean Baizhu decoction pieces into a stir-fry pot, heat them at an extremely high flame, and stir them rapidly until the exterior is black and the inside is yellow. Spray some water when there is a spark, and then spread out and dry.
6) Bran-fried Baizhu: heat a wok and sprinkle wheat bran. When smoke is generated, add Baizhu decoction pieces in the pan until they become brownish; then, remove the wheat bran and cool the product. This method can neutralize the effect of invigorating the *spleen*, which is mainly used for treating abnormal transportation, less food fullness, and other diseases.
7) Fried Baizhu with honey bran: place the honey in a pot and heat it until boil. Add wheat bran such that the ratio of wheat bran to honey is 5:1. Fry the bran until it can be kneaded into a ball. Stir the Baizhu and fry until the surface is browned. The honey bran is removed and cooled. This method exerts the best effect because the nature and flavor of honey play the roles of nourishing and invigorating the *spleen.*

Studies have shown that a few chemical components of Baizhu undergo changes after processing. Chen et al. determined the chemical components in several processed Baizhu samples, and the results showed that the content of volatile oil changed. The volatile oil content levels were as follows: raw Baizhu > fried Baizhu with honey and bran > bran-fried Baizhu > soil-fried Baizhu > fried Baizhu. They also found that Atractylodes lactones content increased (Chen et al., 2005). The method also provides Baizhu concocted using an objective evaluation method. Atractylon, Atractylodes lactone I, Atractylodes lactone III, and biatractylolide content were determined before and after processing atractylon into Atractylodes lactone I and Atractylodes lactone III (Yu et al., 2005). Weng et al. studied the effects of different processed products of Baizhu on gastric emptiness and intestinal propulsion in mice with spleen deficiency. The results showed that the processing group experienced significant inhibition of gastric emptying rate and small intestinal propulsion rate in mice with spleen deficiency, and the effect was better than that observed in the raw Baizhu group (Weng et al., 2015). This may be the reason why raw Baizhu can promote gastrointestinal peristalsis better than processed Baizhu, a phenomenon that also provides a basis for the clinical use of raw Baizhu in the treatment of constipation.

Studies have shown that the volatile oil content of Baizhu is reduced after processing (Jia et al., 1998), and the volatile oil content of Baizhu promotes intestinal motility. After processing, the content of volatile oil in Baizhu was significantly reduced, which reduced the stimulation of the gastrointestinal tract and boosted the spleen strengthening effect in mice with spleen deficiency. However, the content of Atractylodes lactones increased after processing (Wen et al., 1999; Li et al., 2006b), which has been considered to be the main component of spleen strengthening. This enhanced the spleen strengthening effect of Baizhu in mice with spleen deficiency. The polysaccharide content in different soil-fried Baizhu was determined *via* ultraviolet spectrophotometry. The results showed that there was a significant difference between processed and raw products and that the polysaccharide content of Baizhu increased after it was processed with different auxiliary materials and soil; however, there was no significant difference between the processed products (Chen et al., 2010). This indicated that the processing of Baizhu exerted an effect on the polysaccharide composition. Zhang et al. studied the effects of different processed products of Baizhu on the movement of the small intestine in mice and found that, compared with the decoction of stir-fried Baizhu and burnt Baizhu, the decoction of raw Baizhu demonstrated a remarkable function of promoting small intestinal movement in animals (Zhang and Dou, 2005). Before and after processing, the composition of Baizhu is regarded as the basis of its therapeutic effect change, and certain studies have found that the processing time of Baizhu exerts a substantial influence on the composition change. These studies on the processed products of Baizhu show that the processed products of Baizhu present with potential utility in the development of drugs and clinical application. Processing is a skill that is inherited as a part of the legacy from ancient China; records of processing methods provide evidence for the techniques that can be used for the development of the efficacy of TCMs and expand the scope of TCM. It is evident that the development of the processing technology of Baizhu will also be the focus of future research.

## Toxicity and Pharmacokinetics

### Toxicity

Acute toxicity tests show that Baizhu possesses minor toxicity (Zhou et al., 1996). Yang et al. observed the toxic reactions caused by continuous and repeated administration of the volatile oil of Baizhu and conducted long-term toxicity tests using the volatile oil of Baizhu in rats. Rats were divided into high-dose and low-dose groups (300 mg/kg and 100 mg/kg of the volatile oil emulsion of Baizhu), and the control group was subjected to continuous intragastric administration for 3 months. The results showed that administration of these two doses of the volatile oil emulsions for three consecutive months was safe (Yang et al., 2003), further indicating that its toxicity is less and its long-term use does not present with considerable side effects. After the administration of Baizhu in stroke-prone rats with spontaneous hypertension, hematological, blood biochemical, and histopathological tests were performed within 1 month. No abnormalities were found, confirming that it was safe and effective (Yi, 2005). Modern pharmacology researchers conducted experiments on acute toxicity, cytotoxicity, and genetic toxicity of the extract of Baizhu through animal experiments and obtained desirable findings, indicating that the acute toxicity of the water extract of Baizhu is relatively low, with relatively low cytotoxicity and no genetic toxicity (Zhao et al., 2006). Li et al. performed an acute toxicity test of Baizhu extract (extracted with water and then precipitated with ethyl alcohol). It was found that the intraperitoneal and intramuscular injections of the extract of Baizhu were safe and could be used in clinical trials (Wang et al., 2011). Hu et al. also found that AMP-pretreated nerve cells within 1.0 g/L did not present with any toxic effect (Hu et al., 2014). There is a lack of clinical studies that directly support its safety in humans; however, the existing information shows the absence of serious adverse events. These studies show that Baizhu is safe, can be applied in clinical practice, and can be considered reducing the toxicity and side effects of drug use.

### Pharmacokinetics

The pharmacokinetic characteristics of Baizhu decoction (the ratio of extract yielded: 34%; Baizhu extract: 28.2 g/kg) were observed by intraperitoneal injection of mice at different intervals and were as follows: 1) the distribution rate constant was greater than the elimination rate constant, and the drug was distributed rapidly to tissues; 2) the elimination half-life of Baizhu was long and the CLB was inversely proportional to it, indicating that the elimination of Baizhu *in vivo* was slow; 3) the central compartment transport rate constant was greater than the peripheral compartment transport rate constant. The central compartment distribution volume was greater than the peripheral ventricular distribution volume, indicating that drugs were distributed in blood, extracellular fluid, liver, kidneys, and other tissues with rich blood vessels and unobstructed blood flow; and 4) the considerable apparent volume of distribution indicated that Baizhu was widely distributed in the body (Deng, 1993). Atractylodes lactone III was studied *in vivo*. The plasma C-T curve showed the pattern of a two-compartment model of first-order absorption after disposable lavage on SD rats with Atractylodes lactone III (100 mg/kg). According to the pharmacokinetic parameters of Atractylodes lactone III, it was rapidly absorbed by oral administration. T_max_ was 0.85 ± 0.01 h, elimination half-life was 0.85 ± 0.01 h, volume of apparent distribution was 5.48 ± 0.23 L/kg, and total clearance of plasma was 4.63 ± 0.65 L/hkg, indicating that Atractylodes lactone Ⅲ was rapidly balanced *in vivo* distribution and was rapidly cleared in the blood. At 1.5 h after administration, the concentration distribution in the main effector organs was in the order C_lung_ > C_epencephalon_ > C_heart_ > C_brain_, and the concentration distribution in the main eliminated organs was characterized as C_spleen_ > C_liver_ > C_renal_ (Li CQ. et al, 2006; Zhu et al., 2018). Atractylodes lactone III was absorbed quickly in rats and the elimination rate was fast. The distribution in the lungs and Atractylodes lactone Ⅲ levels in the body provided a reference basis for future researches. Using an *in vivo* intestinal circulation model and HPLC method to study Atractylodes lactone I in the absorption characteristics of various intestinal segments of rats, results showed that there was no significant difference in absorption rate constants of Atractylodes lactone I in duodenum, jejunum, ileum, and colon of rats. After adding P-gp inhibitors verapamil and digoxin, the absorption rate constant of Atractylodes lactone I was significantly increased (Tsuji and Tamai, 1996). The use of P-gp inhibitors can compete with the drug at the P-gp-binding site, may help reduce the outflow of the drug, and may promote drug absorption. The absorption of Atractylodes lactone I is affected by P-gp present outside epithelial cells, emerging as a mechanism in rat small intestine transit for passive diffusion, and there is no special absorption window (Wang C. et al, 2009).

The latest study conducted *via* the UPLC-MS/MS method for simultaneous determination of Atractylodes lactones I, II, and III and their pharmacokinetics in rats *in vivo* presented several remarkable results. Results showed that the methodology validation for the extracted lactones Ⅰ, Ⅱ, and Ⅲ was appreciable. The retention times of Atractylodes lactones I, II, and III and internal standard were 2.55, 3.1, 1.8, and 1.2 min, respectively. The linear ranges of Atractylodes lactone I, II, and III were 0.1–100 ng/ml, 0.138–138 ng/ml, and 0.141–141 ng/ml, respectively. Accuracy exceeded 95%, while intraday and intraday precision values were less than 10%. The RSD of the matrix effect was less than 10%, which exerted no influence on sample analysis. The extraction recoveries exceeded 79%. Atractylodes lactones I, II, and III were absorbed rapidly into the blood, could be detected at 0.0833 h, and could last for approximately 12 h in plasma. The first peak time of Atractylodes lactone I and II components was close, with a demonstration of peak time at 0.5 h, and the peak time of Atractylodes lactone III components was 1 h. It was also found that AUC_0_-t of Atractylodes lactones I, II, and III in rats was positively correlated with the dose (Xu et al., 2020). Most studies have been conducted on Atractylodes lactone I, II, and III pharmacokinetic studies, and pharmacokinetic studies of other ingredients are lacking. However, it can be implied through the existing literature that Baizhu pharmacokinetic parameters in the body are good and can help provide an important basis for clinical applications.

## Conclusion and Future Perspectives

As an important Chinese medicinal material, Baizhu presents with a long history of use and demonstrates many pharmacological effects. Additionally, the clinical application, toxicity, and pharmacokinetics of Baizhu are briefly described, but there remain several deficiencies.

1) Baizhu exerts multiple pharmacological effects, but most experiments have focused on its gastrointestinal effects. There are few studies on other pharmacological effects, and the reported data and information are relatively few; this provides more research possibilities to counter a lack of information. Further researches should focus on the mechanism underlying the pharmacological action of Baizhu.
2) Baizhu has a complex chemical composition, but most studies have focused on the same, few elements, such as Atractylodes lactone Ⅰ, Atractylodes lactone Ⅱ, Atractylodes lactone Ⅲ, atractylon, and others; essentially, a class of volatile oil has been studied. The other components also warrant extensive development and research. Among them, lactones are the main bioactive ingredients, while volatile oils are unstable and warrant further studies. The UPLC fingerprint is used to establish a scientific quality standard for Baizhu. Much remains unknown about the structure and biological activity of different polysaccharides; therefore, further researches are needed.
3) There are few reports available on the use of Baizhu alone for the treatment of diseases. In most studies, Baizhu is combined with other crude drugs to treat certain diseases, and this is also in line with the traditional application of crude drugs. These studies also explained the dosage of crude drugs combined with Baizhu. The mechanism of action and synergies between Baizhu and different compounds also should be clarified. It lays a good foundation for explaining the compatibility principle scientifically and for promoting the development of modern TCM. It is also possible to further study the effective action form and synergistic effect of medicinal substances based on the same pharmacophore, to better understand the medicinal substances and mechanism of action of TCMs.
4) Processing is a traditional drug processing method in ancient China, and there are many processing methods of Baizhu recorded in ancient books, and it has been stated that the processing of Baizhu will have an impact on its chemical composition and pharmacological action, which may be one of the main directions for future researches on Baizhu.
5) Toxicity tests showed that the toxicity of Baizhu was extremely low, with almost no toxicity, which might be one of the reasons for the observation of lower toxicity and fewer side effects in the clinical application of the Baizhu compound. However, the toxicity and safety of the chemical components in the extracts have not been determined, and further experiments are warranted.
6) There are few studies available on Baizhu *in vivo*, but it has been found that only a few chemical components in Baizhu can be easily detected, and this creates obstacles for pharmacokinetic experimental studies *in vivo*; however, pharmacokinetic studies of these chemical components *in vivo* should be further explored.

Baizhu is a TCM with a wide range of applications, and has been used for a long period; however, there are also many chemical components and pharmacological effects that have not been developed, which suggests that studies should be conducted for the exploration of other chemical components and pharmacological effects of Baizhu in future researches. These may also be future research directions, and the limited potential should be investigated further.

## References

[B1] Cai H. Guo Y. Zhao Z. Chen Y. Zhao S. Chen B. (2018). Banxia Baizhu Tianma Decoction for Hyperlipidemia: Protocol for a Systematic Review and Meta-Analysis. Medicine (Baltimore) 97 (44), e13067. 10.1097/MD.0000000000013067 30383686 PMC6221624

[B2] Cao G. Zhang X. Y. Cong X. D. Zhang Y. Cai B. C. (2009). Research Progress of Polysaccharides from Baizhu. J. Beijing Union Univ. 23 (3), 14–18. 10.16255/j.cnki.ldxbz.2009.03.014

[B3] Chao C. L. Huang H. C. Lin H. C. Chang T. C. Chang W. L. (2016). Sesquiterpenes from Baizhu Stimulate Glucose Uptake by Activating AMPK and PI3K. Am. J. Chin. Med. 44 (5), 963–979. 10.1142/S0192415X16500531 27430917

[B4] Chen B. B. (2012). Progress in Pharmacological Research of Atractylodes. Inner Mongolia J. Traditional Chin. Med. 31 (10), 101–102. 10.16040/j.cnki.cn15-1101.2012.10.042

[B5] Chen H. P. Liu Y. P. Liu C. P. Li P. (2010). Comparison of the Content of Atractylodes Lactone Ⅲ and Atractylodes Polysaccharide in Different Stir-Fried Baizhu. China Pharm. 21 (39), 3680–3683.

[B6] Chen J. Sun Y. C. Ran X. K. Yuan Y. Dou D. Q. (2016). Study on Diuretic Effect of Baizhu. Mod. Chin. Med. 18 (5), 563–567. 10.1007/s11655-016-2610-2

[B7] Chen M. Z. Zhang Y. (1961). Diuresis of Atractylodes. Acta Physiol. Sinica 24 (3-4), 227–237.

[B8] Chen X. Liu Y. Deng Y. Yuan B. Li Y. Ren X. (2015). Hemorheological Study and Treatment with Enema Retention of Li Chong Tang Combined with Moxibustion in Women Suffering from Chronic Pelvic Inflammatory Diseases. Pak J. Pharm. Sci. 28 (4), 1465–1469. 10.13862/j.cnki.cn43-1446/r.2005.07.044 26431659

[B9] Chen Y. Y. Zhang T. S. Zhong Q. (2005). Quality Comparison of Different Processed Products of Baizhu. Guiding J. Traditional Chin. Med. Pharm. 11 (7), 86–87.

[B10] Chinese Pharmacopoeia Commission (2020). The 2020 Edition of Pharmacopoeia of the People’s Republic of China, Vol. 1. Beijing, China: Chemical Industry Press.

[B11] Chou Y. J. Kuo C. Y. Chen P. Y. Chen L. L. Yeh K. Y. Kuo H. S. (2012). Oral Administration of Qing-Shu-Yi-Qi-Tang Reduce Lung Cancer-Induced Cachexia in Mice. Afr. J. Pharm. Pharmacol. 6 (2), 84–91. 10.5897/ajpp11.186

[B12] Dai G. H. Wang C. P. (2007). Treatment of 106 Cases of Chronic Eczema in Children by Shenling Baizhu Powder Combined with External Washing of Traditional Chinese Medicine. China Med. Herald 4 (20), 95–96. 10.3969/j.issn.1673-7210.2007.20.072

[B13] Deng X. L. (1993). To Investigate Pharmacokinetics of Baizhu by Drug Accumulation *In Vivo* . J. Southwest Minzu Univ. (Natural Sci. Edition) 1993 (1), 61–84.

[B14] Ding S. Q. Ding Y. J. Zhang S. M. Wang Y. H. Zheng X. P. (2005). Observation on the Curative Effect of Decoction of Baizhu on 36 Cases of Colonic Slow Transit Constipation. J. New Chin. Med. 37 (9), 30–31. 10.3969/j.issn.1674-7860.2015.14.013

[B15] Dong F. C. (2015). Pharmacological Effects of Different Chemical Components of Atractylodes. Clin. J. Chin. Med. 7 (14), 28–29. 10.3969/j.issn.1674-7860.2015.14.013

[B16] Dong H. He L. Huang M. Dong Y. (2008). Anti-inflammatory Components Isolated from Atractylodes Macrocephala Koidz. Nat. Prod. Res. 22 (16), 1418–1427. 10.1080/14786410801931629 19023804

[B17] Du C. Y. Choi R. C. Dong T. T. Lau D. T. Tsim K. W. (2014). Yu Ping Feng San, an Ancient Chinese Herbal Decoction, Regulates the Expression of Inducible Nitric Oxide Synthase and Cyclooxygenase-2 and the Activity of Intestinal Alkaline Phosphatase in Cultures. PLOS ONE 9 (6), e100382. 10.1371/journal.pone.0100382 24967898 PMC4072625

[B18] Du C. Y. Zheng K. Y. Bi C. W. Dong T. T. Lin H. Tsim K. W. (2015). Yu Ping Feng San, an Ancient Chinese Herbal Decoction, Induces Gene Expression of Anti-viral Proteins and Inhibits Neuraminidase Activity. Phytother Res. 29 (5), 656–661. 10.1002/ptr.5290 25586308

[B19] Du P. F. Nie J. H. (2004). Review of the Dried Rhizome of Atractylodes Macrocephala Research. J. Pharm. Res. 23 (9), 41–43. 10.3969/j.issn.1672-7738.2004.09.019

[B20] Duan Q. Li C. P. Zhao Z. D. Ma L. (2009). Determination of Atractylodes Lactone Ⅰ, Ⅱ, Ⅲ in Stir-Fried Baizhu by High-Performance Liquid Chromatography. Cent. South Pharm. 7 (5), 321–323.

[B21] Feng G. Q. (2001). Preliminary Study on Biaxially Regulating Gastrointestinal Function of Baizhu. Mod. Traditional Chin. Med. 1, 24–25. 10.3969/j.issn.1672-0571.2001.01.017

[B22] Feng X. Wang Z. L. Lin Y. C. Zhou Y. Liu Y. Z. Yang H. Z. (2009). Effect of Biatractylenolide on Aβ1-40 Induced Dementia Rats. Chin. Pharmacol. Bull. 25 (7), 949–951. 10.3321/j.issn:1001-1978.2009.07.029

[B23] Feng Z. F. Tang S. H. Guo L. J. He L. Yang R. B. (2019). Polysaccharide from Baizhu Inhibits the Growth of HT-29 Orthotopic Xenograft of colon Cancer Cells in Mice by Activating Immune Cells. Chin. J. Cancer Biother. 26 (11), 1209–1213.

[B24] Gao H. Zhu X. Xi Y. Li Q. Shen Z. Yang Y. (2018). Anti-depressant-like Effect of Atractylenolide I in a Mouse Model of Depression Induced by Chronic Unpredictable Mild Stress. Exp. Ther. Med. 15 (2), 1574–1579. 10.3892/etm.2017.5517 29434743 PMC5776173

[B25] Gao Q. (2017). Neuroprotective Effect of Baizhu on Focal Cerebral Ischemia/reperfusion in Rats. J. Med. Theor. Pract. 30 (15), 2185–2186. 10.19381/j.issn.1001-7585.2017.15.001

[B26] Gao Q. Yu X. Y. (2016b). Effect of Baizhu on Cognitive Ability and Expression of Related Signaling Proteins in Brain Aging Mice. J. Heze Med. Coll. 28 (1), 1–3. + 45. 10.3969/j.issn.1008-4118.2016.01.001

[B27] Gao Q. Yu X. Y. (2016a). Effect of Baizhu on Memory Ability and Synaptic Structure in Brain Aging Mice. J. Heze Med. Coll. 28 (2), 1–3. + 14.

[B28] Gao X.-L. Wang B.-Y. Chen Y.-L. Zai Y.-B. Bai M. (2013). Atractylenolide II Significantly Reduces Proliferation of Mouse colon Cancer Cells. Wcjd 21 (26), 2690–2693. 10.11569/wcjd.v21.i26.2690

[B29] Gao X. L. Wang B. Y. Yin S. G. Zhang Z. Y. Chen Y. L. (2015). Effect of Atractylodes Lactone on Proliferation of IEC-6 Cells and Esophageal Cancer Cell ECA9706. China J. Traditional Chin. Med. Pharm. 30 (3), 921–923.

[B30] Ge X. Y. Wu S. H. (2006). Treating 36 Cases of Infantile Anorexia with Shenlingbaizhu Powder Modified. Henan Traditional Chin. Med. 26 (7), 63. 10.16367/j.issn.1003-5028.2006.07.046

[B31] Guan X. H. Qu X. Yang Z. P. Huang W. L. Sun H. X. (2001). Effect of Volatile Oil of Baizhu on Immune Function in Mice. J. Beihua Univ. (Natural Science) 2, 122–124. 10.3969/j.issn.1009-4822.2001.02.008

[B32] Guo F. Huang L. Zhou S. (2007). Headspace Solid-phase Microextraction-Gas Chromatography-Mass Spectrometry for Analysis of Volatile Components from Atractlodes Macrocephala Koidz. Se Pu 25 (1), 43–47. 10.3321/j.issn:1000-8713.2007.01.009 17432574

[B33] Guo Z. Su Z. Wang Z. Luo X. Lai R. (2017). The Effect of Chinese Herbal Medicine Banxia Baizhu Tianma Decoction for the Treatment of Vertebrobasilar Insufficiency Vertigo: A Systematic Review and Meta-Analysis of Randomized Controlled Trials. Complement. Ther. Med. 31, 27–38. 10.1016/j.ctim.2017.01.004 28434468

[B34] Guo Z. X. Liang Z. H. Lou L. Q. Zhang L. P. (2012). Study on Extraction, Purification and Activity of Polysaccharide from Baizhu. J. Anhui Agric. Sci. 40 (24), 2011–2013. 10.13989/j.cnki.0517-6611.2012.24.022

[B35] Hao F. Y. Hao F. X. (2004). Shenling Baizhu Powder in the Treatment of Simple Dyspepsia in Children. Chin. Community Doctors (comprehensive edition) 6 (1), 40–41.

[B36] Hao X. B. (2017). Clinical Study on the Treatment of Vascular Dementia with Cerebral Collateral-Stasis by Modified Pinellia Atractylodes Tianma Decoction Combined with Moxibustion. Chronic Pathematology J. 18 (5), 579–581. 10.16440/j.cnki.1674-8166.2017.05.035

[B37] Hao Y. J. Sang Y. L. Li B. L. Wang L. Jia T. Z. (2006). Effect of Atractylenolide Ⅰ and Atractylenolide Ⅲ on Salivary Amylase Activity. Lishizhen Med. Materia Med. Res. 17 (9), 1617–1618. 10.3969/j.issn.1008-0805.2006.09.002

[B38] Heinrich M. Appendino G. Efferth T. Fürst R. Izzo A. A. Kayser O. (2020). Best Practice in Research - Overcoming Common Challenges in Phytopharmacological Research. J. Ethnopharmacol 246, 112230. 10.1016/j.jep.2019.112230 31526860

[B39] Hu W. X. Xiang Q. Fu A. Z. Wen Z. He D. Hu G. Z. (2014). Effect of Polysaccharide of Baizhu on Neuronal Cytotoxicity and Anti-hypoxia-induced Neuronal Apoptosis. Pharmacol. Clin. Chin. Materia Med. 30 (3), 89–91. 10.13412/j.cnki.zyyl.2014.03.028

[B40] Huang H. L. Chen C. C. Yeh C. Y. Huang R. L. (2005a). Reactive Oxygen Species Mediation of Baizhu-Induced Apoptosis in Human Leukemia Cells. J. Ethnopharmacol 97 (1), 21–29. 10.1016/j.jep.2004.09.058 15652270

[B41] Huang Y. Y. Ding X. Y. Sun W. Li X. Chen T. Wang Q. (2005b). Effect of Topical Application of Atractylodes Decoction on Serum TNF-α Content in Inflammatory Mice. J. Beijing Univ. Traditional Chin. Med. 28 (6), 57–58. 10.3321/j.issn:1006-2157.2005.06.017

[B42] Huang Z. R. Wang Y. Wang R. P. Chen J. J. (2010). Effect of Shenlingbaizhu Powder on Serum IL-2, IFN-γ and TNF-α in Tumor-Bearing Mice. Guangming J. Chin. Med. 25 (9), 1584–1586. 10.3969/j.issn.1003-8914.2010.09.021

[B43] Hwang J. M. Tseng T. H. Hsieh Y. S. Chou F. P. Wang C. J. Chu C. Y. (1996). Inhibitory Effect of Atractylon on Tert-Butyl Hydroperoxide Induced DNA Damage and Hepatic Toxicity in Rat Hepatocytes. Arch. Toxicol. 70 (10), 640–644. 10.1007/s002040050323 8870957

[B44] Jia T. Z. Wang Y. N. Xu M. (1998). Comparison of Thin Layer and GC-MS of Volatile Oil before and after Processing of Atractylodes. Liaoning J. Traditional Chin. Med. 25 (9), 47–48. 10.13192/j.ljtcm.1998.09.47.jiatzh.037

[B45] Jiang H. Shi J. Li Y. (2011). Screening for Compounds with Aromatase Inhibiting Activities from Atractylodes Macrocephala Koidz. Molecules 16 (4), 3146–3151. 10.3390/molecules16043146 21494203 PMC6260643

[B46] Jin C. Zhang P. J. Bao C. Q. Gu Y. L. Xu B. H. Li C. W. (2011). Protective Effects of Atractylodes Macrocephala Polysaccharide on Liver Ischemia-Reperfusion Injury and its Possible Mechanism in Rats. Am. J. Chin. Med. 39 (3), 489–502. 10.1142/S0192415X11008981 21598417

[B47] Kim C. K. Kim M. Oh S. D. Lee S. M. Sun B. Choi G. S. (2011). Effects of Atractylodes Macrocephala Koidzumi Rhizome on 3T3-L1 Adipogenesis and an Animal Model of Obesity. J. Ethnopharmacol 137 (1), 396–402. 10.1016/j.jep.2011.05.036 21669278

[B48] Kim H. G. Han J. M. Lee H. W. Lee J. S. Son S. W. Choi M. K. (2013). CGX, a Multiple Herbal Drug, Improves Cholestatic Liver Fibrosis in a Bile Duct Ligation-Induced Rat Model. J. Ethnopharmacol 145 (2), 653–662. 10.1016/j.jep.2012.12.005 23228913

[B49] Kim H. G. Kim J. M. Han J. M. Lee J. S. Choi M. K. Lee D. S. (2014). Chunggan Extract, a Traditional Herbal Formula, Ameliorated Alcohol-Induced Hepatic Injury in Rat Model. World J. Gastroenterol. 20 (42), 15703–15714. 10.3748/wjg.v20.i42.15703 25400454 PMC4229535

[B50] Kiso Y. Tohkin M. Hikino H. (1983). Antihepatotoxic Principles of Atractylodes Rhizomes. J. Nat. Prod. 46 (5), 651–654. 10.1021/np50029a010 6418860

[B51] Li C. He L. (2006). Establishment of the Model of white Blood Cell Membrane Chromatography and Screening of Antagonizing TLR4 Receptor Component from Atractylodes Macrocephala Koidz. Sci. China C Life Sci. 49 (2), 182–189. 10.1007/s11427-006-0182-7 16704122

[B52] Li C. Q. He L. C. Deng T. (2006c). Pharmacokinetics and Tissue Distribution of Atractylenolide III in Rats. Zhong Yao Cai 29 (8), 807–809. 10.13863/j.issn1001-4454.2006.08.024 17076240

[B53] Li C. Q. He L. C. Dong H. Y. Jin J. Q. (2007b). Screening for the Anti-inflammatory Activity of Fractions and Compounds from Atractylodes Macrocephala Koidz. J. Ethnopharmacol 114 (2), 212–217. 10.1016/j.jep.2007.08.002 17869038

[B54] Li C. Q. He L. C. Jin J. Q. (2007a). Atractylenolide I and Atractylenolide III Inhibit Lipopolysaccharide-Induced TNF-Alpha and NO Production in Macrophages. Phytother Res. 21 (4), 347–353. 10.1002/ptr.2040 17221938

[B55] Li D. Du S. J. Yu Y. Q. (2016b). Effect of Atractylodes Lactone Ⅰ on Related Inflammatory Factors in Rats with Chronic Atrophic Gastritis. Med. Pharm. J. Chin. People's Liberation Army 28 (8), 10–14. 10.3969/j.issn.2095-140X.2016.08.003

[B56] Li H. (2005). Clinical Observation on the Treatment of 64 Cases of Cirrhosis Ascites with Addition and Subtraction of Qiwei Baizhu Powder Combined with Diuretic. Guiding J. Traditional Chin. Med. Pharm. 11 (5), 16–17. 10.13862/j.cnki.cn43-1446/r.2005.05.007

[B57] Li J. (2009). Clinical Observation on the Treatment of Cerebral Infarction Assisted by Tianma Decoction of Pinellia Ternata Atractylodes. Chin. J. Rural Med. Pharm. 16 (1), 44–45. 10.3969/j.issn.1006-5180.2009.01.034

[B58] Li J. C. Lv Z. L. Shi Y. H. Shen Y. Chen Y. F. (1996b). Drug Regulation and Computer Image Processing of the Peritoneal Foramen. Acta Academiae Medicinae Sinicae 18 (3), 219–223. 9388996

[B59] Li J. Feng J. (1997). Summary of Research on Dual Pharmacological Effects of Single Chinese Herbal Medicine on Lowering Blood Pressure and Blood Lipid. Lishizhen Med. Materia Med. Res. 8 (1), 69–70.

[B60] Li N. Xu Z. Zhang H. Zhao Y. (2008). “New Triterpene Prosaponin and Sapogenin with Unsaturated Lactone Skeleton from Gynostemma Pentaphfllum and the Antitumor Activities Assay,” in Proceedings of the 2008 International Conference on Gin-Seng (Changchun, Jilin, China: IEEE), 1–3.

[B61] Li Q. C. Tang Y. G. Gu C. Z. Li X. H. Deng Y. L. Qin T. (2015a). Study on the Curative Effect of Guizhiqugui and Fuling Baizhu Decoction on Septic Acute Kidney Injury. China J. Mod. Med. 25 (11), 63–66. 10.3969/j.issn.1005-8982.2015.11.014

[B62] Li Q. H. Ma X. T. Xie C. Gong X. Q. (2007c). Study on Fingerprint of Atractylodes. Chin. Traditional Herbal Drugs 38 (6), 929–931. 10.3321/j.issn:0253-2670.2007.06.052

[B63] Li R. Fu C. M. Zhou R. R. Gao M. H. Li Y. R. Tao X. H. (2020). Meta-analysis of Banxia Baizhu Tianma Decoction for Meniere’s Disease. World Chin. Med. 15 (21), 3266–3271. 10.3969/j.issn.1673-7202.2020.21.013

[B64] Li S. Lin Z. W. Zhu Z. Q. (2009). Treatment of 87 Cases of Ulcerative Colitis with Baizhu Shaoyao Powder Combined with Traditional Methods. Strait Pharm. J. 21 (8), 130. 10.3969/j.issn.1006-3765.2009.08.070

[B65] Li W. Zhi W. Liu F. He Z. Wang X. Niu X. (2017). Atractylenolide I Restores HO-1 Expression and Inhibits Ox-LDL-Induced VSMCs Proliferation, Migration and Inflammatory Responses *In Vitro* . Exp. Cell Res 353 (1), 26–34. 10.1016/j.yexcr.2017.02.040 28274716

[B66] Li W. Wen H. M. Cui X. B. Li X. Hong M. (2006a). Study on Effective Components of Atractylodes for Invigorating Spleen. J. Nanjing Univ. Traditional Chin. Med. 22 (6), 366–367. 10.3969/j.issn.1000-5005.2006.06.009

[B67] Li W. Wen H. M. Cui X. B. Zhang F. C. (2006b). Effect of Processing on the Content of Atractylodes Lactone Ⅱ in Atractylodes. J. Chin. Med. Mater. 5, 497–498. 10.13863/j.issn1001-4454.2006.05.036

[B68] Li W. Wen H. M. Cui X. B. Zhang F. C. (2005). HPLC Method Was Used to Determine the Content of Atractylodes Ⅱ in Atractylodes. Res. Pract. Chin. Medicines 19 (5), 35–36. 10.3969/j.issn.1006-3765.2009.08.070

[B69] Li W. Wen H. M. Zhang A. H. Ge J. H. Wu H. (2001). Study on Quality Standard of Atractylodes Ⅰ - HPLC Method for Determination of Two Kinds of Atractylodes Lactone. Chin. J. Pharm. Anal. 21 (3), 170–172. 10.1007/s11769-001-0027-z

[B70] Li X. Liu F. Li Z. Ye N. Huang C. Yuan X. (2014). Atractylodes Macrocephala Polysaccharides Induces Mitochondrial-Mediated Apoptosis in Glioma C6 Cells. Int. J. Biol. Macromol 66 (2), 108–112. 10.1016/j.ijbiomac.2014.02.019 24556116

[B71] Li X. Wei G. Wang X. Liu D. H. Deng R. D. Li H. (2012). Targeting of the Sonic Hedgehog Pathway by Atractylenolides Promotes Chondrogenic Differentiation of Mesenchymal Stem Cells. Biol. Pharm. Bull. 35 (8), 1328–1335. 10.1248/bpb.b12-00265 22863933

[B72] Li Y. Chen S. H. Ji X. Geng Z. G. Lv G. Y. (2015b). Effects of Polysaccharides from Atractylodes Macrocephala on Blood Glucose and Related Indexes in Mice with Spontaneous Type 2 Diabetes. Chin. J. Exp. Traditional Med. Formulae 21 (10), 162–165. 10.13422/j.cnki.syfjx.2015100162

[B73] Liu C. Zhao H. Ji Z. H. Yu X. Y. (2014). Neuroprotection of Atractylenolide III from Atractylodis Macrocephalae against Glutamate-Induced Neuronal Apoptosis via Inhibiting Caspase Signaling Pathway. Neurochem. Res. 39 (9), 1753–1758. 10.1007/s11064-014-1370-7 24958167

[B74] Liu H. C. (2009). Clinical Observation of Different Doses of Atractylodes in the Treatment of Constipation after Anorectal Disease. Chin. J. Mod. Drug Appl. 3 (5), 101–102. 10.3969/j.issn.1673-9523.2009.05.092

[B75] Liu H. Zhong Q. (2006). Clinical Observation of 55 Cases of Transient Ischemic Attack Treated by Integrated Traditional Chinese and Western Medicine. Shandong J. Traditional Chin. Med. 25 (1), 46–47. 10.16295/j.cnki.0257-358x.2006.01.025

[B76] Liu L. S. Wang R. Xu R. H. Shang N. Wang Y. Fan Q. (2010). Growth Promoting Effect and Structure Analysis of Polysaccharides from Atractylodes on Probiotics. Food Sci. 31 (19), 124–128.

[B77] Liu T. Yang L. L. Zou L. Li D. F. Wen H. Z. Zheng P. Y. (2013a). Chinese Medicine Formula Lingguizhugan Decoction Improves Beta-Oxidation and Metabolism of Fatty Acid in High-Fat-Diet-Induced Rat Model of Fatty Liver Disease. Evid. Based Complement. Alternat Med. 2013, 429738. 10.1155/2013/429738 23762134 PMC3664975

[B78] Liu Y. Liao C. M. (2006). Study on the Effect of Diatractylolide on Mice with Dementia Induced by Aluminum Trichloride. J. Hunan Normal Univ. (Medical Sciences) 3 (3), 25–27. 10.3969/j.issn.1673-016X.2006.03.008

[B79] Liu Y. P. Hu M. L. Zhang H. T. Shen Y. S. (2007). Observation on the Therapeutic Effect of Modified Banxia Baizhu Tianma Decoction in the Treatment of Middle-Aged Essential Hypertension. Inf. Traditional Chin. Med. 24 (1), 27–28. 10.3969/j.issn.1002-2406.2007.01.011

[B80] Long F. Y. Jia P. Wang H. F. Qing Y. He M. J. Wang X. L. (2017). Mechanisms and Proliferation Inhibitory Effects of Atractylenolide Ⅰ on SK-OV-3 and OVCAR-3 Ovarian Cancer Cell. J. Reg. Anat. Oper. Surg. 26 (2), 89–93.

[B81] Lu J. J. (2016). A GC-MS Analysis of Volatile Components of Atractylodes Macrocephala Koidz and Their Research of Inhibitory Activity on Five Tumor Cells. Strait Pharm. J. 28 (6), 28–31.

[B82] Lu J. M. (2006). 40 Cases of Obesity Treated with Traditional Chinese Medicine and Auricular point Sticking. Jilin J. Chin. Med. 26 (3), 48. 10.13463/j.cnki.jlzyy.2006.03.037

[B83] Ma Q. H. Zhang P. X. Guo H. Y. Wei X. D. Ou Q. (2006). Antioxidant Effect of Polysaccharide from Atractylodes on Nerve Cells Induced by D-Galactose in Rats. Chin. J. Gerontol. 26 (12), 1658–1660. 10.3969/j.issn.1005-9202.2006.12.030

[B84] Ma X. L. Mei H. M. (2007). Effect of Polysaccharide of Atractylodes on Physiological Function of Isolated Frog Heart. J. Huangshan Univ. 9 (3), 94–96. 10.3969/j.issn.1672-447X.2007.03.028

[B85] Mao L. Z. (1994). The Bidirectional Regulating Effect of Buzhong Yiqi Decoction. Chin. J. Traditional Chin. Med. 9 (5), 33–34.

[B86] Meng S. X. Liu Q. Tang Y. J. Wang W. J. Zheng Q. S. Tian H. J. (2016). A Recipe Composed of Chinese Herbal Active Components Regulates Hepatic Lipid Metabolism of NAFLD *In Vivo* and *In Vitro* . Biomed. Res. Int. 2016, 1026852. 10.1155/2016/1026852 27069915 PMC4812184

[B87] Miao L. J. Yang Y. Z. Xing H. Y. Cheng G. Z. (2017). Clinical Effect Observation of Pinellia Atractylodes Tianma Decoction Combined with Captopril in Treating Hypertension. Inner Mongolia Med. J. 49 (5), 601–604. 10.16096/J.cnki.nmgyxzz.2017.49.05.041

[B88] Nemere I. (1995). Nongenomic Effects of 1,25-dihydroxyvitamin D3: Potential Relation of a Plasmalemmal Receptor to the Acute Enhancement of Intestinal Calcium Transport in Chick. J. Nutr. 125 (6), 1695S–1698S. 10.1093/jn/125.suppl_6.1695S 7782929

[B89] Nikles S. Monschein M. Zou H. Liu Y. He X. Fan D. (2017). Metabolic Profiling of the Traditional Chinese Medicine Formulation Yu Ping Feng San for the Identification of Constituents Relevant for Effects on Expression of TNF-α, IFN-γ, IL-1β and IL-4 in U937 Cells. J. Pharm. Biomed. Anal. 145, 219–229. 10.1016/j.jpba.2017.03.049 28667937

[B90] Ning J. L. Zhong X. Q. (2009). The Treatment of 80 Cases of Concussion by Pinellia Atractylodes Tianma Decoction and Nimodipine. J. Guangxi Univ. Chin. Med. 12 (1), 37–38. 10.3969/j.issn.1008-7486.2009.01.020

[B91] Ninomiya I. Yonemura Y. Matsumoto H. Sugiyama K. Kamata T. Miwa K. (1991). Expression of C-Myc Gene Product in Gastric Carcinoma. Oncology 48 (2), 149–153. 10.1159/000226915 1997938

[B92] Peng F. L. (2009). Treatment of 82 Cases of Chronic Enteritis with Shenling Baizhu Powder Modified. Yunnan J. Traditional Chin. Med. Materia Med. 30 (5), 42–43. 10.16254/j.cnki.53-1120/r.2009.05.019

[B93] Peng M. Gu S. J. Jiang L. J. Fang N. B. Yu S. G. (2011). Study on the Effective Part of Atractylodes Macrocephala Extract in Lowering Blood Lipid. Shizhen Traditional Chin. Med. Materia Med. 22 (10), 2363–2365. 10.3969/j.issn.1008-0805.2011.10.020

[B94] Pu H. L. Wang Z. L. Huang Q. J. Xu S. B. Lin Y. C. Wu X. Y. (2000). Effect of Diatractylodide on Isolated guinea Pig Atrial Muscle. Chin. Pharmacol. Bull. 16 (1), 60–62. 10.3321/j.issn:1001-1978.2000.01.020

[B95] Qiu X. M. Lu Y. H. Shen P. Liu A. X. Zhang Y. Li L. L. (2005). Determination of Polysaccharide Content of Atractylodes from Different Producing Areas. ’ China Pharm. 14 (4), 40–41. 10.3969/j.issn.1006-4931.2005.04.030

[B96] Rivera D. Allkin R. Obón C. Alcaraz F. Verpoorte R. Heinrich M. (2014). What Is in a Name? the Need for Accurate Scientific Nomenclature for Plants. J. Ethnopharmacol 152 (3), 393–402. 10.1016/j.jep.2013.12.022 24374235

[B97] Shan J. J. Tian G. Y. (2003). Studies on Physico-Chemical Properties and Hypoglycemic Activity of Complex Polysaccharide AMP-B from Atractylodes Macrocephala Koidz. Yao Xue Xue Bao 38 (6), 438–441. 10.1023/A:1022865704606 14513804

[B98] Shen G. Q. He F. L. Li F. X. Sun Y. Zhang H. G. (2009). Experimental Study on Chemical Constituents and Antitumor of Volatile Oil from Atractylodes. J. Beijing Univ. Traditional Chin. Med. 32 (6), 413–415. 10.3321/j.issn:1006-2157.2009.06.015

[B99] Shi N. Su J. Yang Z. B. Lv G. Y. Chen S. H. (2014). Antioxidant Effect of Polysaccharides from Atractylodes on D-Galactose Induced Aging Model Mice. Chin. J. New Drugs 23 (5), 577–584.

[B100] Shi W. R. Liu Y. Chen L. Tu C. X. Zhang Z. L. (2007). Study on the Effect of Dryness and Dampness of Atractylodes. J. Fujian Univ. Traditional Chin. Med. 17 (3), 29–31.

[B101] Shimato Y. Ota M. Asai K. Atsumi T. Tabuchi Y. Makino T. (2018). Comparison of Byakujutsu (Atractylodes Rhizome) and Sojutsu (Atractylodes Lancea Rhizome) on Anti-inflammatory and Immunostimulative Effects *In Vitro* . J. Nat. Med. 72 (1), 192–201. 10.1007/s11418-017-1131-4 28983786

[B102] Shou D. Dai S. Zhang J. Li H. Yu Z. (2008). Simultaneous Determination of Atractylenolide III, Atractylenolide I and Atractylon in Artactylodis Macrocephala Using Microbore Liquid Chromatography. Se Pu 26 (5), 637–639. 10.1002/cjoc.200890112 19160769

[B103] Son M. J. Kim Y. E. Song Y. I. Kim Y. H. (2017). Herbal Medicines for Treating Acute Otitis media: A Systematic Review of Randomised Controlled Trials. Complement. Ther. Med. 35, 133–139. 10.1016/j.ctim.2017.11.001 29154058

[B104] Song H. P. Li R. L. Chen X. Wang Y. Y. Cai J. Z. Liu J. (2014). Atractylodes Macrocephala Koidz Promotes Intestinal Epithelial Restitution via the Polyamine-VvoltageGgated K+ Channel Pathway. J. Ethnopharmacol 152 (1), 163–172. 10.1016/j.jep.2013.12.049 24417867

[B105] Song L. Y. Gu J. M. (2007). Experimental Study on the Effect of Different Processing Methods on Anti-aging of Atractylodes. Mod. Med. J. China 9 (11), 15–17. 10.3969/j.issn.1672-9463.2007.11.005

[B106] Sun W. P. Li F. S. Chen C. Luo H. Yang G. Liu H. (2011). Study on the Regulation of Immune Function of Mice by Atractylodes Polysaccharide. Chin. J. Microecology 23 (10), 881–882. 10.13381/j.cnki.cjm.2011.10.012

[B107] Sun W. P. Li F. S. Hou D. D. Liu H. (2008). Effects of Angelica Sinensis, Atractylodes Macrocephala and White Aconite Polysaccharide on Immunomodulatory Effect in Mice. Chin. J. Inf. Traditional Chin. Med. 15 (7), 37–38. 10.3969/j.issn.1005-5304.2008.07.017

[B108] Sun X. (2008). Observation on the Curative Effect of Peptic Ulcer in 50 Cases. Bright Chin. Med. 23 (6), 818. 10.3969/j.issn.1003-8914.2008.06.070

[B109] Sun Y. N. (1999). Progress in Pharmacological experiment and Clinical Study of Atractylodes. Chin. J. Traditional Chin. Med. Sci. Tech. 4, 279–280.

[B110] Tan C. S. Loh Y. C. Ng C. H. Ch'ng Y. S. Asmawi M. Z. Ahmad M. (2018). Anti-hypertensive and Vasodilatory Effects of Amended Banxia Baizhu Tianma Tang. Biomed. Pharmacother. 97, 985–994. 10.1016/j.biopha.2017.11.021 29136777

[B111] Tang Y. M. (2007). 55 Cases of Slow Transit Constipation Were Mainly Treated with Raw Atractylodes. J. Liaoning Univ. Traditional Chin. Med. 9 (6), 103. 10.13194/j.jlunivtcm.2007.06.105.tangym.049

[B112] Tsuji A. Tamai I. (1996). Carrier-mediated Intestinal Transport of Drugs. Pharm. Res. 13 (7), 963–977. 10.1023/a:1016086003070 8842032

[B113] Wan Y. Shen Y. M. Zou J. F. Chen M. J. Zhang Z. M. Jiang S. (2021). Study on Intestinal Absorption Characteristics of Five Main Active Components from Lizhong Decoction Extract. Acta Pharmaceutica Sinica 56 (6), 1689–1695. 10.16438/j.0513-4870.2021-0097

[B114] Wang A. Xiao Z. Zhou L. Zhang J. Li X. He Q. (2016a). The Protective Effect of Atractylenolide I on Systemic Inflammation in the Mouse Model of Sepsis Created by Cecal Ligation and Puncture. Pharm. Biol. 54 (1), 146–150. 10.3109/13880209.2015.1024330 25853971

[B115] Wang C. Duan H. He L. (2009a). Inhibitory Effect of Atractylenolide I on Angiogenesis in Chronic Inflammation *In Vivo* and *In Vitro* . Eur. J. Pharmacol. 612 (1-3), 143–152. 10.1016/j.ejphar.2009.04.001 19356732

[B116] Wang C. C. Chen L. G. Yang L. L. (2002a). Cytotoxic Activity of Sesquiterpenoids from Atractylodes Ovata on Leukemia Cell Lines. Planta Med. 68 (3), 204–208. 10.1055/s-2002-23144 11914954

[B117] Wang D. S. Li J. Y. Luo C. Y. Zheng J. F. Zhang S. D. Hu Z. Y. (2011). Acute Toxicity Test of Extract of Atractylodes. Acta Agriculturae Boreali-Occidentalis Sinica 20 (5), 40–43. 10.3969/j.issn.1004-1389.2011.05.008

[B118] Wang F.-Y. Zhong L. L. D. Kang N. Dai L. Lv L. Bian L.-Q. (2016b). Chinese Herbal Formula for Postprandial Distress Syndrome: Study Protocol of a Double-Blinded, Randomized, Placebo-Controlled Trial. Eur. J. Integr. Med. 8 (5), 688–694. 10.1016/j.eujim.2016.03.010

[B119] Wang G. F. Li Y. Y. Shi R. Wang T. M. Li Y. F. Li W. K. (2020a). Yinchenzhufu Decoction Protects against Alpha-Naphthylisothiocyanate-Induced Acute Cholestatic Liver Injury in Mice by Ameliorating Disordered Bile Acid Homeostasis and Inhibiting Inflammatory Responses. J. Ethnopharmacol 254, 112672. 10.1016/j.jep.2020.112672 32084553

[B120] Wang G. W. Feng J. Qiu X. M. Liu Y. L. (2009c). Effect of Polysaccharide from Atractylodes on Cerebral Edema after Traumatic Brain Injury in Rats and its Mechanism. Chin. Herbal Medicines 40 (6), 948–950. 10.3321/j.issn:1002-6630.2008.12.158

[B121] Wang H. Y. (2008). Therapeutic Effect of Shenlingbaizhu Powder on 60 Cases of Cirrhosis. Sichuan Traditional Chin. Med. 28 (2), 64–65. 10.1016/s0254-6272(09)60006-6

[B122] Wang J. H. (2007). 60 Cases of Ulcerative Functional Dyspepsia Treated with Atractylodes and Hawthorn Decoction. Chin. J. Mod. Drug Appl. 4 (4), 3. 10.3969/j.issn.1673-9523.2007.04.051

[B123] Wang J. J. Wang R. G. (2004). Progress in Antitumor Research of Atractylodes and its Compound. Chin. J. Inf. Traditional Chin. Med. 11 (10), 927–928. 10.3969/j.issn.1005-5304.2004.10.049

[B124] Wang K. T. Chen L. G. Wu C. H. Chang C. C. Wang C. C. (2010a). Gastroprotective Activity of Atractylenolide III from Atractylodes Ovata on Ethanol-Induced Gastric Ulcer *In Vitro* and *In Vivo* . J. Pharm. Pharmacol. 62 (3), 381–388. 10.1211/jpp.62.03.0014 20487223

[B125] Wang L. M. Zhou Y. H. Yang Y. Du W. F. Ge W. H. Li C. Y. (2020b). Optimization of Determination Method for Multi-index Components of Atractylodes. J. Zhejiang Chin. Med. Univ. 44 (7), 657–667. 10.16466/j.issn1005-5509.2020.07.012

[B126] Wang Q. Fan W. T. (2005). Research Progress of Atractylodes in Regulating Gastrointestinal Motility. Mod. Traditional Chin. Med. 1 (1), 65–66.

[B127] Wang R. Zhou G. Wang M. Peng Y. Li X. (2014). The Metabolism of Polysaccharide from Atractylodes Macrocephala Koidz. And its Effect on Intestinal Microflora. Egypt: Evidence-Based Complementary and Alternative Medicine. 10.1155/2014/926381 PMC4258363 25505927

[B128] Wang X. Liu Y. Y. Shi T. L. Zhang H. X. Yang G. W. (2002b). Study on the Effect of Volatile Oil of Atractylodes on Solid Tumor. Chin. Remedies Clin. 2 (4), 239–240. 10.3969/j.issn.1671-2560.2002.04.014

[B129] Wang X. Y. (2015). A Randomized Parallel Controlled Study of Buzhong Yiqi Decoction in the Treatment of Insomnia. J. Pract. Chin. Med. Intern. Med. 29 (1), 42–43. 10.13729/j.issn.1671-7813.2015.01.19

[B130] Wang X. Y. (2009). Treatment of 34 Cases of Female Idiopathic Edema with Shenlingbaizhu Powder. Health Vocational Edu. 27 (10), 109. 10.3969/j.issn.1671-1246.2009.10.081

[B131] Wang Y. J. Su Y. J. (2009). Inhibitory Effect and Mechanism of Volatile Oil from Atractylodes on Hepatocellular Metastasis of H22 Liver Cancer in Mice. Shaanxi Traditional Chin. Med. 30 (6), 735–736. 10.3969/j.issn.1000-7369.2009.06.072

[B132] Wang Z. Li R. L. Xu S. F. Chen W. W. (2010b). Effect of Sugar Complex of Atractylodes on Differentiation and Villus Protein Expression in IEC-6 Cells. Chin. Med. Mater. 33 (6), 938–944. 10.13863/j.issn1001-4454.2010.06.037 21049619

[B133] Wen H. M. Zhang A. H. Wang L. Wu H. Li W. (1999). Effect of Processing on the Content of Atractylodes Lactone Ⅰ in Atractylodes. Chin. Med. Mater. 22 (3), 125–126. 10.13863/j.issn1001-4454.1999.03.011

[B134] Weng P. Wang W. K. Zhang X. T. (2015). Effects of Different Processed Products of Atractylodes on Gastrointestinal Function in Mice with Spleen Deficiency. Jiangxi J. Traditional Chin. Med. 46 (5), 30–32.

[B135] Wu H. G. Zhu L. Ma Y. J. (2005). Experimental Study on the Effect of Atractylodes on Gastrointestinal Activities in Mice. Jiangsu J. Traditional Chin. Med. 26 (11), 66–67. 10.13424/j.cnki.jsctcm.2015.01.036

[B136] Wu T. T. Li R. L. Zeng D. Hu L. Shi Y. X. Wang D. X. (2017). Effect of Atractylodes Macrocephala Polysaccharides on the Regulation of Calcium Ions to Promote Cell Migration and E-Cadherin Expression. New Chin. Med. Clin. Pharmacol. 28 (2), 145–150. 10.19378/j.issn.1003-9783.2017.02.01

[B137] Xiang X. L. Xu D. N. Cao N. Qian L. Tian Y. B. Li W. Y. (2020). Repair Effect of Polysaccharide from Atractylodes on the Number and Function of white Blood Cells in Immunosuppressed Mice Induced by Cyclophosphamide. Chin. J. Vet. Med. 56 (7), 2+42–47.

[B138] Xu L. L. Yao Z. M. Wang D. L. Zhou Y. Y. Zhang M. Li C. H. (2020). Study on the Pharmacokinetic Parameters of Atractylodes LactoneⅠ, Ⅱ, Ⅲ in Rats Based on UPLC-MS/MS. J. Yunnan Univ. Traditional Chin. Med. 43 (3), 17–23. 10.19288/j.cnki.issn.1000-2723.2020.03.004

[B139] Xu Q. Wang Y. Guo S. Shen Z. Wang Y. Yang L. (2014). Anti-inflammatory and Analgesic Activity of Aqueous Extract of Flos Populi. J. Ethnopharmacol 152 (3), 540–545. 10.1016/j.jep.2014.01.037 24508857

[B140] Xu X. J. (2006). 58 Cases of Gastrointestinal Dysfunction after Treatment with Shenling Baizhu Powder for Malignant Tumor of Digestive System. Jilin J. Traditional Chin. Med. 26 (1), 22. 10.13463/j.cnki.jlzyy.2006.01.017

[B141] Xu Y. Cai H. Cao G. Duan Y. Pei K. Tu S. (2018). Profiling and Analysis of Multiple Constituents in Baizhu Shaoyao San before and after Processing by Stir-Frying Using UHPLC/Q-TOF-MS/MS Coupled with Multivariate Statistical Analysis. J. Chromatogr. B Analyt Technol. Biomed. Life Sci. 1083, 110–123. 10.1016/j.jchromb.2018.03.003 29529536

[B142] Yan W. L. Wang S. S. Ren X. (2011a). Experimental Study on Regulating Effect of Atractylodes on Intestinal flora of Mice. Shandong J. Traditional Chin. Med. 30 (6), 417–419. 10.16295/j.cnki.0257-358x.2011.06.036

[B143] Yan Y. Chou G. X. Hui Wang H. Chu J. H. Fong W. F. Yu Z. L. (2011b). Effects of Sesquiterpenes Isolated from Largehead Atractylodes Rhizome on Growth, Migration, and Differentiation of B16 Melanoma Cells. Integr. Cancer Ther. 10 (1), 92–100. 10.1177/1534735410378660 20713377

[B144] Yang C. P. Lao Y. X. Wu F. W. Su W. W. (2002). Research Progress of Atractylodes. J. Traditional Chin. Med. 25 (3), 206–208. 10.13863/j.issn1001-4454.2002.03.028 14746339

[B145] Yang G. W. Zhu X. Q. Wang X. Liu Y. Y. Zhang J. X. Shi T. L. (2003). Study on the Long-Term Toxicity of Volatile Oil of Atractylodes by Gastric Gavage in Rats. Chin. Remedies Clin. 3 (5), 411–412. 10.3969/j.issn.1672-397X.2005.11.042

[B146] Ye Y. Cheng X. M. Chou G. X. Wang Z. T. (2009). Study on Fingerprint of Atractylodes by HPLC. Jiangsu J. Traditional Chin. Med. 41 (4), 59–60. 10.3969/j.issn.1672-397X.2009.04.038

[B147] Yi Y. (2005). Study on Weight Loss and Subacute Toxicity of Atractylodes. Int. J. Traditional Chin. Med. Materia Med. 5, 313.

[B148] Yin R. Wang J. G. (2019). Study on the Regulating Effect and Mechanism of Atractylodes Polysaccharide on Immune Function of Lung Cancer Model Rats. Chin. J. Pharm. Biotechnol. 14 (6), 527–533. 10.3969/j.issn.1673-713X.2019.06.009

[B149] Yu P. Fei Y. Li H. Y. Wang N. N. Shou D. (2017). To Evaluate the Effect of Bran Fried Processing Technology on the Efficacy of Atractylodes by the Inhibition of Intestinal Muscle *In Vitro* . Chin. J. Traditional Chin. Med. 35 (5), 1091–1093. 10.13193/j.issn.1673-7717.2017.05.010

[B150] Yu X. Y. Qiu S. P. (2007). Shenling Baizhu Powder for Treating 30 Cases of Uremia Combined with Malnutrition. New Chin. Med. 23 (1), 145. 10.13457/j.cnki.jncm.2006.12.033

[B151] Yu Y. M. Song C. Y. Jia T. Z. (2005). Study on Quality Standard of Processing Baizhu. Chin. Traditional Patent Med. 27 (6), 669–672. 10.3969/j.issn.1001-1528.2005.06.016

[B152] Zhang C. X. Zhang Y. J. Jiang B. Long H. Y. Ruan J. Zhu W. N. (2017). The Effects of Atractylodolide Ⅱ on Apoptosis and Expression of PARP1 and Caspase-3 in Colorectal Cancer LoVo Cells. Chin. J. Exp. Formulae 23 (5), 157–161. 10.13422/j.cnki.syfjx.2017050157

[B153] Zhang J. D. Chen J. Zhao X. Ding K. Hao H. B. Zou Y. (2006a). Clinical Observation on 48 Cases of Diabetes Treated by Integrated Traditional Chinese and Western Medicine. J. Intern. Med. Pract. Chin. Med. 20 (6), 627–628. 10.13729/j.issn.1671-7813.2006.06.052

[B154] Zhang S. T. Zhang Z. Z. (2007). Treatment of 67 Cases of Infantile Diarrhea with Qiwei Baizhu Powder. J. Pediatr. Traditional Chin. Med. 3 (6), 28–29. 10.3969/j.issn.1673-4297.2007.06.015

[B155] Zhang W. J. Zhao Z. Y. Chang L. K. Cao Y. Wang S. Kang C. Z. (2021). Atractylodis Rhizoma: A Review of its Traditional Uses, Phytochemistry, Pharmacology, Toxicology and Quality Control. J. Ethnopharmacol 266, 113415. 10.1016/j.jep.2020.113415 32987126 PMC7521906

[B156] Zhang X. L. Wang L. Li J. F. Zou L. (2008). Effect of Atractylodes on Contractile Activity of Human Uterine Smooth Muscle during Pregnancy. J. Wuhan Univ. (Medical Edition) 29 (3), 383–386. 10.3969/j.issn.1671-8852.2008.03.027

[B157] Zhang X. L. Wang L. Xu L. Zou L. (2009). Effect of Atractylodes on Calcium-dependent Potassium Channel Currents in the Membrane of Human Uterine Smooth Muscle during Pregnancy. Matern. Child Health Care China 24 (3), 366–368.

[B158] Zhang Y. Dou Y. Q. (2005). Effects of Different Processed Products of Atractylodes on Small Intestinal Motility in Mice. Chin. J. Med. Tribune 20 (5), 13–14. 10.3969/j.issn.1002-1078.2005.05.012

[B159] Zhang Y. Q. Xu S. B. Lin Y. C. Li Q. Zhang X. Lai Y. R. (2000). Antagonistic Effects of 3 Sesquiterpene Lactones from Atractylodes Macrocephala Koidz on Rat Uterine Contraction *In Vitro* . Acta Pharmacol. Sin 21 (1), 91–96. 11263255

[B160] Zhang Y. Q. Hao T. H. (2009). Treating 33 Cases of Vascular Headache with Modified and Decoction of Pinellia Atractylodes Tianma. J. Shaanxi Coll. Traditional Chin. Med. 32 (5), l4–l5.

[B161] Zhang Y. Q. Xu S. B. Lin Y. C. (1999). Gastrointestinal Inhibition of Atractylodes Lactone Series. Chin. Materia Med. 22 (12), 636–640. 10.13863/j.issn1001-4454.1999.12.012 12571906

[B162] Zhang Z. L. Chen W. W. (2006). Preliminary Study on Pharmacological Effects of Astragalus Membranaceus and Atractylodes on Gastric Mucosa Injury. Chin. J. Pract. Traditional West. Med. 19 (4), 470–471.

[B163] Zhang Z. Zhang H. X. Shi T. L. Liu Y. Y. (2006b). Study on Antitumor Effect of Volatile Oil from Atractylodes. Cancer Res. Clinic 18 (12), 799–800. 10.3760/cma.j.issn.1006-9801.2006.12.003

[B164] Zhao A. S. Sun Y. L. Zhang L. S. (2006). Safety Evaluation of Extract of Atractylodes. Chin. J. Public Health 22 (1), 43–45. 10.11847/zgggws2006-22-01-23

[B165] Zhao G. Jiang Y. X. Chi Y. H. (2017b). Research Progress of Atractylodes in the Treatment of Slow Transit Constipation. J. Qingdao Univ. (Medical Sciences) 53 (1), 124–126. 10.13361/j.qdyxy.201701039

[B166] Zhao H. M. Zhu Q. J. Zheng G. J. Zhang D. Zhang J. (2005). Effect of Atractylodes Macrocephala Extract on Invasion of Human Lung Cancer PG Cells *In Vitro* . J. Shandong Univ. Traditional Chin. Med. 29 (6), 465–466. 10.16294/j.cnki.1007-659x.2005.06.023

[B167] Zhao L. Zhu X. Cong R. Yang X. Zhu Y. (2018). The Protective Effects of Danggui-Baizhu-Tang on High-Fat Diet-Induced Obesity in Mice by Activating Thermogenesis. Front. Pharmacol. 9, 1019. 10.3389/fphar.2018.01019 30258363 PMC6143821

[B168] Zheng L. Zhang M. Qin K. Cai H. Cao G. Cai B. (2015). Simultaneous Determination of 10 Active Components in Baizhu Shaoyao San and its Single Herbs by High-Performance Liquid Chromatography Coupled with Diode Array Detection. J. Chromatogr. Sci. 53 (4), 633–640. 10.1093/chromsci/bmu101 25217704

[B169] Zheng Y. (2002). Study on the Correlation between Different Doses of Atractylodes and Diuresis in Patients with Hepatic Ascites. J. Gansu Univ. Chin. Med. 3, 14–15. 10.3969/j.issn.1003-8450.2002.03.008

[B170] Zhou D. W. Zhou L. Y. Yin L. Y. (1996). Pharmacology and Efficacy of Atractylodes Class. Drugs & Clinic 11 (3), 120–122.

[B171] Zhu B. Zhang Q. L. Hua J. W. Cheng W. L. Qin L. P. (2018). The Traditional Uses, Phytochemistry, and Pharmacology of Atractylodes Macrocephala Koidz.: A Review. J. Ethnopharmacol 226, 143–167. 10.1016/j.jep.2018.08.023 30130541

[B172] Zhu C. Yang C. Guo C. X. Qian J. (2019). Effect of Atractylodes on Proliferation of Gastric Cancer SGC-7901 Side Cell *In Vitro* . J. Community Med. 17 (10), 574–578. 10.19790/j.cnki.JCM.2019.10.05

[B173] Zhu J. Z. Leng E. R. Gui X. Y. Chen D. F. (2000). Experimental Study on the Effect of Chinese Herbs Such as Atractylodes and Agastache Rugosus on Gastric Emptying and Intestinal Propulsion. China J. Basic Med. Traditional Chin. Med. 1, 24–26.

[B174] Zhu J. Z. Zhang J. Xu Q. Z. Zhang Z. J. Leng E. R. Chen D. F. (2001). Study on the Mechanism of Atractylodes Promoting Gastrointestinal Motility in Rats. Chin. J. Clin. Pharm. 10 (6), 365–368. 10.19577/j.cnki.issn10074406.2001.06.009

[B175] Zhu Q. J. Zheng G. J. Zhang D. (2006). Study on the Antitumor Effect and Mechanism of Water Extract of Atractylodes. J. Shandong Univ. Traditional Chin. Med. 30 (1), 69–71. 10.16294/j.cnki.1007-659x.2006.01.028

[B176] Zhuang Z. H. (2018). Identification and Clinical Application of Atractylodes Macrocephala Koidz. Capital Food Med. 25 (10), 100–101.

[B177] Zuo F. Liu W. Wang H. (2007). Shenling Baizhu Powder in the Treatment of 52 Cases of Middle and Late Ankylosing Spondylitis. Tianjin J. Traditional Chin. Med. 24 (3), 207. 10.3969/j.issn.1672-1519.2007.03.031

