# Supplementary material for: A Review of the Ethnopharmacology, Phytochemistry, Pharmacology, Application, Quality Control, Processing, Toxicology, and Pharmacokinetics of the Dried Rhizome of Atractylodes macrocephala
Source: Front Pharmacol. 2021 Nov 3;12:727154. doi: 10.3389/fphar.2021.727154 (PMC8595830; doi:10.3389/fphar.2021.727154)
Supplement: Supplementary file 1 [file Table1.docx]

Table 1. The difference of original plant species of between Baizhu and Cangzhu

| The source plant | Morphological characteristics | Traditional Chinese medicine | Medicinal characteristics |
| --- | --- | --- | --- |
| *Atractylodes macrocephala* | It is thick mass or irregular mass in shape, with 3-13 cm in length and 1.5-7 cm in diameter. The surface is grayish yellow or grayish brown, with irregular nodular protrusions, intermittent longitudinal wrinkles and furrows, fibrous root marks, and sunken disc-shaped stem base and bud marks at the top. It is very hard and not easy to break. It smells fragrant, tastes sweet and slightly pungent, and chews slightly sticky. | Baizhu (the dried rhizome) | It can invigorate *spleen* and *qi*, eliminate *dampness* and promote diuresis, reduce perspiration and prevent miscarriage. It can be used for *spleen* deficiency, anorexia, abdominal distention, diarrhea, dizziness due to phlegm retention, edema. |
| *Atractylodes lancea* | It is irregular beaded or nodular cylindrical in shape. The length is 3-10 cm and the diameter is 1-2 cm. The surface is gray-brown, with wrinkles, transverse curved lines and residual fibrous roots, with stem marks and residual stem base at the top. It is very hard, and when exposed for a long time, it can precipitate white hairy crystals, which is commonly called frosting. The aroma is special, and the taste is slightly sweet, spicy and bitter. | Cangzhu (the dried rhizome) | It can eliminate *dampness* and strengthen *spleen*, dispel *wind* and cold, and improve eyesight. It can be used for damp-blocking middle energizer, abdominal distention, diarrhea, edema, beriberi, rheumatism, cold and night blindness. |
| *Atractylodes chinensis* | It is a lumpy or nodular cylinder in shape, with 4-19 cm in length. The surface is brown and black, and the skin is yellow and brown. It is a bit loose, with yellow-brown oil spots scattered on the cross section, and no white hair-like clean crystals precipitated. The aroma is light, and the taste is bitter and bitter | Cangzhu (the dried rhizome) | It can eliminate *dampness* and strengthen *spleen*, dispel *wind* and cold, and improve eyesight. It can be used for damp-blocking middle energizer, abdominal distention, diarrhea, edema, beriberi, rheumatism, cold and night blindness. |

Table 2. The formulas containing Baizhu and their ingredients

|  | Formula name | Ingredients | Extraction method | Major function | References | Ancient book |
| --- | --- | --- | --- | --- | --- | --- |
| 1 | Baizhu Fuling decoction | Baizhu 15 g  Paeoniae Radix Alba (the dried root of *Paeonia lactiflora* (Paeoniaceae))15 g  Glycyrrhizae Radix et Rhizoma Praeparata cum Melle (the processed product of the dried root and rhizome of *Glycyrrhiza uralensis* (Fabaceae)) 10 g  Zingiberis Rhizoma Recens (the fresh rhizome of *Zingiber officinale* (Zingiberaceae)) 15 g  Jujubae Fructus (the dried ripe fruit of *Ziziphus jujuba* (Rhamnaceae)) four  Poria (the dried sclerotia of *Poria cocos* (Polyporaceae)) 15 g | Water decoction | It can treat the discomfort of muscle traction at the back of the head and fever. In clinical practice, it has been found to have obvious therapeutic effect on acute kidney injury (AKI). | (Gu, Li, Tang, Yin, Yang, Ma, Lian, & Qin, 2015) | Treatise on Febrile Diseases (伤寒论) |
| 2 | Shenling Baizhu powder | Baizhu 15 g  Ginseng Radix et Rhizome (the dried root and rhizome of *Panax ginseng* (Araliaceae)) 100g  Poria 100 g  Dioscoreae Rhizoma (the dried root of *Dioscorea polystachya* (Dioscoreaceae))100g  Lablab Semen Album (the dried mature seed of *Lablab purpureu* (Fabaceae)) 75g (Fried)  Nelumbinis Semen (the dried mature seed of *Nelumbo nucifera* (Nelumbonaceae)) 50g  Coicis Semen (the dried mature kernel of *Coix lacryma-jobi* (Poaceae)) 50g (Fried)  Amomi Fructus (the dried ripe fruit of *Amomum villosum* (Zingiberaceae)) 50g  Platycodonis Radix (the dried root of *Platycodon grandifloras* (Campanulaceae)) 50g  Glycyrrhizae Radix et Rhizoma (the dried root and rhizome of *Glycyrrhiza uralensis* (Fabaceae)) 100g | Crushing and uniformly stirring | Weakness of *spleen* and *stomach*, anorexia, vomiting or diarrhea, tightness of chest, weakness of limbs, emaciation, pale yellow complexion, pale red color on the tongue, slow pulse. | (Wu, Zhou, & Yao, 2015) | Tai Ping Hui Min He Ji Ju Fang(太平惠民和剂局方) |
| 3 | Qingshu Yiqi decoction | Astragali Radix (the dried root of *Astragalus membranaceus* (Fabaceae)) 3g  Cangzhu 3g (Soak and peel)  Cimicifugae Rhizoma (the dried root of *Cimicifuga foetida* (Ranunculaceae)) 3g  Ginseng Radix et Rhizome 1.5g  Alismatis Rhizoma (the dried tuber of *Alisma plantago-aquatica* (Alismataceae)) 1.5g  Massa Medicata Fermentata 1.5g (Fried)  Citri Reticulatae Pericarpium (the dried mature pericarp of *Citrus reticulata* and its cultivated varieties (Rutaceae)) 1.5g  Ophiopogonis Radix (the dried root tuber of *Ophiopogon japonicus* (Liliaceae)) 0.9g  Angelicae Sinensis Radix (the dried root of *Angelica sinensis* (Apiaceae)) 0.9g  Glycyrrhizae Radix et Rhizoma Praeparata cum Melle 0.9g  Citri Reticulatae Pericarpium Viride (the peel of dried young fruit or immature fruit of *Citrus reticulata* and its cultivated varieties (Rutaceae)) 0.75g  Phellodendri Chinensis Cortex (the dried bark of *Phellodendron chinense* (Rutaceae)) 0.9g (Wash with wine and peel)  Puerariae Lobatae Radix (the dried root of *Pueraria lobata* (Fabacea)) 0.75g  Schisandrae Chinensis Fructus (the dried ripe fruit of *Schisandra chinensis* (Schisandraceae)) nine  Baizhu 1.5g | Water decoction | It is often used to treat increasing temperature of baby body in summer. | (Chou, Kuo, Chen, Chen, Yeh, Kuo, & Chang, 2012) | Pi Wei Lun  (脾胃论) |
| 4 | Baizhu shaoyao powder | Baizhu 30 g  Paeoniae Radix Alba 30g  Citri Reticulatae Pericarpium 15g  Saposhnikoviae Radix (the dried root of *Saposhnikovia divaricate* (Apiaceae)) 20g | Water decoction | It can tonify *spleen* and emolliate the *liver*, remove *dampness* and antidiarrheal effects. | (Xu, Cai, Cao, Duan, Pei, Tu, Zhou, Xie, Sun, Zhao, Liu, Wang, & Shen, 2018) | Dan Xi Xin Fa (丹溪心法) |
| 5 | Xiangsha Liujunzi decoction | Baizhu 6g  Ginseng Radix et Rhizome 3g  Poria 6g  Glycyrrhizae Radix et Rhizoma 2g  Citri Reticulatae Pericarpium 2.5g  Pinelliae Rhizoma (the dried tuber of *Pinellia ternate* (Araceae)) 3g  Amomi Fructus 2.5g  Aucklandiae Radix (the dried root of *Aucklandia lappa* (Asteraceae)) 2g | Water decoction | It can treat *spleen* deficiency and phlegm stagnation. | (Wang, & Liu, 2007) | Gu Jin Ming Yi Fang Lun(古今名医方论) |
| 6 | Qiwei Baizhu powder | Baizhu 12g  Ginseng Radix et Rhizome 6g  Poria 2g  Glycyrrhizae Radix et Rhizoma 3g  The leaf of Pogostemonis Herba (the dried aerial parts of *Pogostemon cablin* (Lamiaceae)) 2g  Aucklandiae Radix 6g  Puerariae Lobatae Radix 15g | Water decoction | Treatment of *spleen* and *stomach* weakness, vomiting and diarrhea disease. | (Zhang, & Zhang, 2007) | Xiao Er Yao Zheng Zhi Jue (小儿药证直诀) |
| 7 | Banxia Baizhu Tianma decoction | Baizhu 9g  Pinelliae Rhizoma 4.5g  Gastrodiae Rhizoma (the dried tuber of *Gastrodia elata* (Orchidaceae)) 3g  Poria 3g  Citri Exocarpium Rubrum (the dried outer pericarp of *Citrus reticulata* and its cultivated varieties (Rutaceae)) 3g  Glycyrrhizae Radix et Rhizoma 1.5g | Water decoction | It mainly treats megrim, headache, nausea and vomiting, whitish glossy coating of the tongue. | (Guo, Su, Wang, Luo, & Lai, 2017) | Yi Xue Xin Wu (医学心悟) |
| 8 | Danggui Baizhu decoction | Baizhu 90g  Poria 90g  Angelicae Sinensis Radix 30g  Scutellariae Radix (the dried root of *Scutellaria baicalensis* (Lamiaceae)) 30g  Artemisiae Scopariae Herba (the dried aerial parts of *Artemisia capillaris* (Asteraceae)) 30g  Peucedani Radix (the dried root of *Peucedanum praeruptorum* (Apiaceae)) 60g  Aurantii Fructus Immaturus (the dried young fruit of *Citrus sinensis* (Rutaceae)) (Stir-fry with bran, remove seeds) 60g  Glycyrrhizae Radix et Rhizoma Praeparata cum Melle 60g  Armeniacae Semen Amarum (the dried mature seed of *Prunus armeniaca* (Rosaceae)) 60g (Stir-fried with bran, remove the peel and tip)  Pinelliae Rhizoma 75g (Wash seven times) | Water decoction | Clearing *heat* and *dampness*, subsiding jaundice. | (Zhao, Zhu, Cong, Yang, & Zhu, 2018) | San Yin Fang(三因方) |
| 9 | Yinchen Zhufu decoction | Baizhu 6g  Artemisiae Scopariae Herba 3g  Aconm Lateralis Radix Praeparaia (the processed product of the lateral root of *Aconitum carmichaelii* (Ranunculaceae)) 1.5g  Zingiberis Rhizoma Recens 1.5g  Glycyrrhizae Radix et Rhizoma Praeparata cum Melle 3g  Cinnamomi Cortex (the dried bark of *Cinnamomum cassia* (Lauraceae))1g (remove the peel) | Water decoction | Mainly treating *yin* jaundice syndrome. Modern clinical application in hepatitis, chronic liver failure and other liver diseases, remarkable effect. | (Wang, Li, Shi, Wang, Li, Li, Zheng, Fan, Zou, Zan, Wu, & Ma, 2020a) | Yi Xue Xin Wu(医学心悟) |
| 10 | Li Chong decoction | Baizhu 6g  Dioscoreae Rhizoma 15g  Trichosanthis Radix (the dried root of *Trichosanthes rosthornii* (Cucurbitaceae)) 12g  Anemarrhenae Rhizoma (the dried rhizome of *Anemarrhena asphodeloides* (Liliaceaev)) 12g  Astragali Radix 9g  Sparganii Rhizoma (the dried tuber of *Sparganium stoloniferum* (Typhaceae)) 9g  Curcumae Rhizoma (the dried rhizome of *Curcuma phaeocaulis* (Zingiberaceae)) 9g  Galli Gigerii Endothelium Corneum (the dried inner wall of sand sac of gallus gallus domesticus (Phasianidae)) 9g  Codonopsis Radix (the dried root of *Codonopsis pilosula* (Campanulaceae)) 6g | Water decoction | It can treat the *spleen* and *stomach* deficiency cold, vomiting and anorexia. | (Wang, & Liu, 2007) | Yi Xue Zhong Zheng Can Xi Lu (医学衷中参西录) |
| 11 | Buzhong Yiqi decoction | Baizhu 10g  Astragali Radix 15g  Ginseng Radix et Rhizome 15g  Glycyrrhizae Radix et Rhizoma Praeparata cum Melle 15g  Angelicae Sinensis Radix 10g  Citri Reticulatae Pericarpium 6g  Cimicifugae Rhizoma 6g  Bupleuri Radix (the dried root of *Bupleurum scorzonerifolium* (Apiaceae)) 12g | Water decoction | An effective prescription for improving the digestive system function, quality of life and nutritional status in elderly patients with chronic obstructive pulmonary disease. a representative prescription that is increasingly applied to treat gastrointestinal dysfunction, such as constipation. | (Mao, 1994) | Nei Wai Shang Bian Huo Lun (内外伤辨惑论) |
| 12 | Baizhu decoction | Baizhu 80g  Ginseng Radix et Rhizome 80g  Magnoliae Officinalis Cortex (the dried bark, root bark and branch bark of *Magnolia officinalis* (Magnoliaceae)) 60g (scrape the coarsed cortex, process with ginger juice)  Citri Reticulatae Pericarpium (Remove the tangerine pith) 60g  Cinnamomi Cortex 40g (scrape the coarsed cortex) | Water decoction | The main treatment of *stomach* flatulence, cold sweat symptoms. | (Zhao, Zhu, Cong, Yang, & Zhu, 2018) | Sheng Ji Zong Lu (圣济总录) |
| 13 | Sijunzi decoction | Baizhu 9g  Ginseng Radix et Rhizome 9g  Poria 9g  Glycyrrhizae Radix et Rhizoma 6g | Water decoction | It is a good recipe for treating weak *spleen* and *stomach*, lack of vital energy, poor appetite, pale complexion, weak limbs and weak pulse. | (Wang, & Liu, 2007) | Tai Ping Hui Min He Ji Ju Fang(太平惠民和剂局方) |
| 14 | Linggui Zhugan decoction | Baizhu 6g  Poria 12g  Cinnamomi Cortex (remove the peel) 9g  Glycyrrhizae Radix et Rhizoma Praeparata cum Melle 6g | Water decoction | Prescription for treatment syndromes of middle energizer and stagnant fluid retention. | (Wang, & Liu, 2007) | Synopsis of golden chamber(金匮要略) |
| 15 | Liujunzi decoction | Baizhu 9g  Codonopsis Radix 9g  Poria 9g  Glycyrrhizae Radix et Rhizoma Praeparata cum Melle 6g  Citri Reticulatae Pericarpium 3g  Pinelliae Rhizoma 4.5g | Water decoction | Treating *spleen* deficiency and phlegm stagnation. | (Wang, & Liu, 2007) | Yi Xue Zheng Zhuan (医学正传) |
| 16 | Er Zhu decoction | Baizhu 3g  Cangzhu 4.5g (soak in rice slops)  Arisaematis Rhizome (the dried tuber of *Arisaema heterophyllum* (Araceae)) 3g  Citri Reticulatae Pericarpium 3g  Poria 3g (remove the peel)  Cyperi Rhizoma (the dried rhizome of *Cyperus rotundus* (Cyperaceae)) 3g  Scutellariae Radix 3g  Clematidis Radix et Rhizoma (the dried root and rhizome of *Clematis chinensis* (Ranunculaceae)) 3g  Notopterygii Rhizoma et Radix (the dried rhizomes and root of *Notopterygium inchum* (Apiaceae)) 3g  Glycyrrhizae Radix et Rhizoma 3g  Pinelliae Rhizoma 6g (with ginger processing) | Water decoction | It is effective in treating various diseases and syndromes caused by *spleen* deficiency, *dampness* and *spleen* *yang* deficiency. | (Wang, & Liu, 2007) | Wan Bing Hui Chun (万病回春) |
| 17 | Zhi Zhu decoction | Baizhu 30g  Cinnamomi Cortex 22g (remove the peel)  Aconm Lateralis Radix Praeparaia 30g (remove the peel)  Asari Radix et Rhizoma (the dried root and rhizome of *Asarum heterotropoides* (Aristolochiaceae))30g  Platycodonis Radix 22g  Arecae semen (the dried mature seed of *Areca catechu* (Arecaceae)) 22g  Glycyrrhizae Radix et Rhizoma Praeparata cum Melle 22g  Aurantii Fructus Immaturus 15g | Water decoction | Prescription for *spleen* deficiency and *qi* stagnation. | (Wang, & Liu, 2007) | Chong Ding Yan Shi Ji Sheng Fang (重订严氏济生方) |
| 18 | Yi Ai powder | Baizhu 30g  Pinelliae Rhizoma 30g  Arcae Concha (the shell of Arca subcrenata (Arcidae)) 30g,  Aucklandiae Radix 9g  Draconis Sanguis (the processed resin exuded from the fruit of *Daemonorops draco* (Arecaceae)) 9g,  Realgar (a sulfide mineral (mainly containing arsenic disulfide)) 6g | Crushing and uniformly stirring | In alleviating abdominal pain, abdominal distension, improving appetite, nausea and vomiting. Treating constipation is particularly outstanding. | (Chen, 1997) | (Chen, 1997) |
| 19 | Baizhu ointment | Baizhu 500g  Citri Reticulatae Pericarpium 120g | Decoctionl decocted extract | The *spleen* and *stomach* disharmony, anorexia, stool diarrhea. |  | Yi Xue Ru Men(医学入门) |
| 20 | Baizhu Fuzi decoction | Baizhu 6g  Aconm Lateralis Radix Praeparaia 10g  Glycyrrhizae Radix et Rhizoma Praeparata cum Melle 3g  Zingiberis Rhizoma Recens 4.5g  Jujubae Fructus six | Water decoction | Improving the level of related hormones in patients. | (Ma, 2020) | Synopsis of golden chamber |
| 21 | Chunggan extract (CGX) | Baizhu 3g  Artemisiae Scopariae Herba 5g  Trionycis Carapax (the carapace of Trionyx sinensis Wiegmann (Trionychidae)) 5g  Raphani Semen (the dried mature seed of *Raphanus sativus* (Brassicaceae))5g  Cangzhu 3g  Poria 3g  Alismatis Rhizoma 3g  Salviae Miltiorrhizae Radix et Rhizoma (the dried root and rhizome of *Salvia miltiorrhiza* (Lamiaceae))3g  Polyporus (the dried sclerotia of *Polyporus umbellatus* (Polyporaceae)) 2g  Aurantii Fructus Immaturus 2g  Amomi Fructus 2g  Glycyrrhizae Radix et Rhizoma 1g  Aucklandiae Radix 1g | Water decoction | It has been used in South Korea since 2001 as a remedy for patients with chronic liver disorders, such as alcoholic liver injury. | (Kim, Kim, Han, Lee, Choi, Lee, Park, & Son, 2014) | (Kim, Kim, Han, Lee, Choi, Lee, Park, & Son, 2014) |
| 22 | Wu Ling powder | Baizhu 9g  Polyporus 9g (remove the peel)  Poria 9g  Alismatis Rhizoma 15g  Cinnamomi Cortex 6g (remove the peel) | Crushing and uniformly stirring | It can promote *kidney* function and diuresis | (Yang, Zhang, Liu, Hu, Xue, Ding, & Kong, 2015) | Treatise on Febrile Diseases |
| 23 | Danggui powder | Baizhu 125g  Angelicae Sinensis Radix 250g  Scutellariae Radix 250g  Paeoniae Radix Alba 250g  Chuanxiong Rhizoma (the dried root of *Ligusticum chuanxiong* (Apiaceae)) 250g | Crushing and uniformly stirring | It can treat blood deficiency, *dampness* and heat and threatened abortion. | (Zhang, 2017) | Synopsis of Golden Chamber· The pulse syndrome of pregnancy and treatment |

Table 3. The components of terpenoids and their glycosides

| Chemical compounds | No. | Compound name | Structure | Ref. |
| --- | --- | --- | --- | --- |
| Terpenoids and their glycosides | 1 | Atractyloside A |  | (Kitajima, Kamoshita, Ishikawa, Takano, Fukuda, Isoda, & Ida, 2003b) |
|  | 2 | Atractyloside B |  | (Kitajima, Kamoshita, Ishikawa, Takano, Fukuda, Isoda, & Ida, 2003b) |
|  | 3 | Atractyloside A 14-O-β-D-fructofuranoside |  | (Kitajima, Kamoshita, Ishikawa, Takano, Fukuda, Isoda, & Ida, 2003b) |
|  | 4 | Eremanthin |  | (Toda, Shigemori, Ueda, Miyamoto, 2017) |
|  | 5 | 1-patchoulene-4α,7α-diol |  | (Wang, Liu, Liu, & Gao, 2008a) |
|  | 6 | (1S,4S,5S,7R,10R)-10,11,14-trihydroxyguai-3-one 11-O-β-D-glucopyranoside |  | (Kitajima, Kamoshita, Ishikawa, Takano, Fukuda, Isoda, & Ida, 2003b) |
|  | 7 | (1S,4S,5R,7R,10R)-11,14-dihydroxyguai-3-one11-O-β-D-glucopyranoside |  | (Kitajima, Kamoshita, Ishikawa, Takano, Fukuda, Isoda, & Ida, 2003b) |
|  | 8 | (1S,5R,7R,10R)-secoatractylolactone11-O-β -D-glucopyranoside |  | (Kitajima, Kamoshita, Ishikawa, Takano, Fukuda, Isoda, & Ida, 2003b) |
|  | 9 | (1S,4S,5S,7R,10R)-10.11,14-trihydroxyguai-3-one11-O-β-D-glucopyranoside |  | (Kitajima, Kamoshita, Ishikawa, Takano, Fukuda, Isoda, & Ida, 2003b) |
|  | 10 | (1S,4S,5S,7R,10S)-10,11,14-trihydroxyguai-3-one 11-O-β-D-glucopyranoside |  | (Kitajima, Kamoshita, Ishikawa, Takano, Fukuda, Isoda, & Ida, 2003b) |
|  | 11 | (1S,5R,7R,10R)-secoatractylolactone11-O-β-D-glucopyranoside |  | (Kitajima, Kamoshita, Ishikawa, Takano, Fukuda, Isoda, & Ida, 2003b) |
|  | 12 | (1S,7R,10R)-11,15-dihydroxy-4-guaien-3-one 11-O-β-D-glucopyranoside |  | (Xu, Jiang, Feng, Yang, Li, Zang, & Zhang, 2016a) |
|  | 13 | (1R,7R,10S)-10,11-dihydroxy-4-guaien-3-one 11-O-β-D-glucopyranoside |  | (Xu, Jiang, Feng, Yang, Li, Zang, & Zhang, 2016a) |
|  | 14 | 3,4,11,14-tetrahydroxyguai-9-en-11-O-β-D-glucopyranoside |  | (Chen, 2007) |
|  | 15 | (3R,4R,7R,10R)-2-hydroxypancherione-11-O-β-D-glucopyranoside |  | (Xu, Jiang, Feng, Yang, Li, Zang, & Zhang, 2016a) |
|  | 16 | 4α,7α-epoxyguaiane-10α,11-diol |  | (Wang, Liu, Liu, & Gao, 2008a) |
|  | 17 | (5R,7R,10S)-3-O-β-D-glucopyranosylisopterocarpolone-11-O-β-D-apiofur- anosyl-(1→6)-β-D-glucopyranoside |  | (Jiang, Xu, Feng, Yang, & Zhang, 2018) |
|  | 18 | 7α,10α-epoxyguaiane-4α,11-diol |  | (Wang, Liu, Liu, & Gao, 2008a) |
|  | 19 | 10-epi-atractylosideA |  | (Kitajima, Kamoshita, Ishikawa, Takano, Fukuda, Isoda, & Ida, 2003b) |
|  | 20 | 10β,11β-epoxyguaiane-1α, 4α-diol |  | (Wang, Liu, Liu, & Gao, 2008a) |
|  | 21 | 10β,11β-epoxyguaiane-1α,4α,7α-triol |  | (Wang, Liu, Liu, & Gao, 2008a) |
|  | 22 | Atractylon |  | (Resch, Steigel, Chen, & Bauer, 1998) |
|  | 23 | Atractylodes lactone I |  | (Resch, Steigel, Chen, & Bauer, 1998) |
|  | 24 | Atractylodes lactone II |  | (Resch, Steigel, Chen, & Bauer, 1998) |
|  | 25 | Atractylodes lactone III |  | (Hikino, Hikino, Yosioka, 1964) |
|  | 26 | Atractylodes lactone IV |  | (Hikino, Hikino, Yosioka, 1964) |
|  | 27 | Atractyloside C |  | (Kitajima, Kamoshita, Ishikawa, Takano, Fukuda, Isoda, & Ida, 2003b) |
|  | 28 | Atractyloside D |  | (Kitajima, Kamoshita, Ishikawa, Takano, Fukuda, Isoda, & Ida, 2003b) |
|  | 29 | Atractyloside E |  | (Kitajima, Kamoshita, Ishikawa, Takano, Fukuda, Isoda, & Ida, 2003b) |
|  | 30 | Atractyloside F |  | (Yahara, Higashi, Iwaki, Nohara, Marubayashi, Ueda, Kohda, Goto, Izumi, Nuno, Katsuki, Isoda, & Satake, 1989) |
|  | 31 | Atractyloside G |  | (Kitajima, Kamoshita, Ishikawa, Takano, Fukuda, Isoda, & Ida, 2003b) |
|  | 32 | Atractyloside I |  | (Kitajima, Kamoshita, Ishikawa, Takano, Fukuda, Isoda, & Ida, 2003b) |
|  | 33 | Atractyloside G 2-O-β-D-glucopyranoside |  | (Kitajima, Kamoshita, Ishikawa, Takano, Fukuda, Isoda, & Ida, 2003b) |
|  | 34 | Cis-atractyloside I |  | (Kitajima, Kamoshita, Ishikawa, Takano, Fukuda, Isoda, & Ida, 2003b) |
|  | 35 | Eudesm-4 (15)-ene-7α,11-diol |  | (Wang, Liu, Liu, & Gao, 2008a) |
|  | 36 | Eudesm-4 (15),7-diene-9α,11-diol |  | (Wang, Liu, Liu, & Gao, 2008a) |
|  | 37 | Eudesm-4 (15),7-diene-11-ol-9-one |  | (Wang, Liu, Liu, & Gao, 2008a) |
|  | 38 | Eudesma-4(14),7(11)-dien-8-one |  | (Endo & Hikino, 1979) |
|  | 39 | Eudesm-7 (11)-en-4-ol |  | (Hong, Kim, & Kim, 2012) |
|  | 40 | (+) Eudesma-4(14),7(11)-dien-8-one |  | (Endo & Hikino, 1979) |
|  | 41 | Kudtdiol |  | (Kamauchi, Kinoshita, Takatori, Sugita, Takahashi, & Koyama, 2015) |
|  | 42 | Officinoside C |  | (Kitajima, Kamoshita, Ishikawa, Takano, Fukuda, Isoda, et al., 2003a) |
|  | 43 | Pterocarpol |  | (Yahara, Higashi, Iwaki, Nohara, Marubayashi, Ueda, Kohda, Goto, Izumi, Nuno, Katsuki, Isoda, & Satake, 1989) |
|  | 44 | *β*-eudesmol |  | (Duan, Wang, Qian, Su, & Tang, 2008) |
|  | 45 | (1R,7R,10R)-1-hydroxylcarissone-11-O-β-D-glucopyranoside |  | (Xu, 2017) |
|  | 46 | 2,11,13-trihydroxy-*β*-selinene |  | (Kamauchi, Kinoshita, Takatori, Sugita, Takahashi, & Koyama, 2015) |
|  | 47 | (2R,3R,5R,7R,10S)-Atractyloside G 2-O-*β*-Glucopyranoside |  | (Kitajima, Kamoshita, Ishikawa, Takano, Fukuda, Isoda, & Ida, 2003b) |
|  | 48 | (2R,7R,10S)-2-hydroxylcarissone-11-O-β-D-glucopyranoside |  | (Xu, 2017) |
|  | 49 | 3α-hydroxy-pterocarpol |  | (Kamauchi, Kinoshita, Takatori, Sugita, Takahashi, & Koyama, 2015) |
|  | 50 | (3S)-3-hydroxyatractylenolide III 3-O-D-glucopyranoside |  | (Kitajima, Kamoshita, Ishikawa, Takano, Fukuda, Isoda, et al., 2003a) |
|  | 51 | (3S,4R,5S,7R)-13-hydroxylhinesolone-11-O-*β*-D-glucopyranoside |  | (Xu, 2017) |
|  | 52 | 4 (15),11-eudesmadien |  | (Van Minh, Van Kiem, Huong, Lee, & Kim, 2004) |
|  | 53 | (5R,7R,10S)-isopterocarpolone-*β*-D-glucopyranoside |  | (Kitajima, Kamoshita, Ishikawa, Takano, Fukuda, Isoda, & Ida, 2003b) |
|  | 54 | (5R,7R,10S)-3-hydroxylisopterocarpolone-3-O-*β*-D-glucopyranoside |  | (Xu, 2017) |
|  | 55 | (5R,7R,10S)-6′′-O-*β*-D-apiofuranosylatractyloside I |  | (Xu, 2017) |
|  | 56 | (5R,7R,10S)-6′′-O- acetylatractyloside I |  | (Xu, 2017) |
|  | 57 | (5R,7R,10S)-6′-O- acetylatractyloside I |  | (Xu, 2017) |
|  | 58 | (5R,7R,10S)- isopterocarpolone-11-O-*β*-D-apiofuranosyl-(1→6)-*β*-D-glucopyranoside |  | (Xu, 2017) |
|  | 59 | (5R,7R,10S)-14-hydroxylisopterocarpolone-11-O-*β*-D-glueopyranoside |  | (Xu, 2017) |
|  | 60 | (5R,7R,10S)-14-carboxylisopterocarpolone-11-O-*β*-D-glucopyranoside |  | (Xu, 2017) |
|  | 61 | (11R)-2,11,12-trihydroxy-*β*-selinene |  | (Kamauchi, Kinoshita, Takatori, Sugita, Takahashi, & Koyama, 2015) |
|  | 62 | 14-hydroxy-isopterocarpolone |  | (Kamauchi, Kinoshita, Takatori, Sugita, Takahashi, & Koyama, 2015) |
|  | 63 | Hinesol |  | (Hashimoto, Noma, Kato, Tanaka, Takaoka, Asakawa, 1999) |
|  | 64 | Hinesolone |  | (Long, Wang, Qi, Yang, & Gao, 2020) |
|  | 65 | 2-oxo-12-hydroxy-hinesol |  | (Kamauchi, Kinoshita, Takatori, Sugita, Takahashi, & Koyama, 2015) |
|  | 66 | 2-oxo-15-hydroxy-hinesol |  | (Kamauchi, Kinoshita, Takatori, Sugita, Takahashi, & Koyama, 2015) |
|  | 67 | (3S,4R,5S,7R)-13-hydroxylhinesolone-11-O-*β*-D-glucopyranoside |  | (Xu, Yang, Feng, Jiang, & Zhang, 2016b) |
|  | 68 | (3S,4R,5R,7R)-3,11-dihydroxy-11,12-dihydronootkatone-11-O-*β*-D-glucopyranoside |  | (Xu, Yang, Feng, Jiang, & Zhang, 2016b) |
|  | 69 | (3S,4R,5S,7R)-3,4,11-trihydroxy-11,12-dihydronootkatone-11-O-*β*-D- glucopyranoside |  | (Xu, Yang, Feng, Jiang, & Zhang, 2016b) |
|  | 70 | (4S,5S,7R)-15-hydroxylhinesolone-15-O-*β*-D-xylopyranoside |  | (Xu, Yang, Feng, Jiang, & Zhang, 2016b) |
|  | 71 | (4S,5S,7R)-14-hydroxylhinesolone-14-O-*β*-D-xylopyranoside |  | (Xu, Yang, Feng, Jiang, & Zhang, 2016b) |
|  | 72 | (5R,7R)-14-hydroxy-3,4-dehydrohinesolone-14-O-*β*-D-xylopyranoside |  | (Xu, Yang, Feng, Jiang, & Zhang, 2016b) |
|  | 73 | (5R,7R)-14-hydroxy-3,4-dehydrohinesolone-11-O-*β*-D-apiofuranosyl-(1→6)-*β*-D-glucopyranoside |  | (Xu, Yang, Feng, Jiang, & Zhang, 2016b) |
|  | 74 | (5R,7R)-14-hydroxy-3,4-dehydrohinesolone-11-O-*β*-D-glueopyranoside |  | (Xu, Yang, Feng, Jiang, & Zhang, 2016b) |
|  | 75 | (7R)-3,4-dehydrohinesolone-11-O-*β*-D-glucopyranosideosideyranoside- hydroxyeud |  | (Xu, Yang, Feng, Jiang, & Zhang, 2016b) |
|  | 76 | (7R)-3,4-dehydrohinesolone-11-O-*β*-D-glucopyranoside |  | (Xu, Yang, Feng, Jiang, & Zhang, 2016b) |
|  | 77 | Daucosterol |  | (Duan, Wang, Qian, Su, & Tang, 2008) |
|  | 78 | Oleanolic acid |  | (Duan, Wang, Qian, Su, & Tang, 2008) |
|  | 79 | Stigmasterol |  | (Duan, Wang, Qian, Su, & Tang, 2008) |
|  | 80 | Stigmasterol 3-O-*β*-D-glucopyranoside |  | (Duan, Wang, Qian, Su, & Tang, 2008) |
|  | 81 | Traxerol acetate |  | (Duan, Wang, Qian, Su, & Tang, 2008) |
|  | 82 | *β*-sitosterol |  | (Duan, Wang, Qian, Su, & Tang, 2008) |
|  | 83 | Stigmasterol 3-O-*β*-D-glucopyranoside  Traxerol acetate  *β*-sitosterol |  | (Duan, Wang, Qian, Su, & Tang, 2008) |

Table 4. The components of polyacetylenes of diene-diyne types and their glycosides.

| Chemical compounds | No. | Compound name | Structure | Ref. |
| --- | --- | --- | --- | --- |
| Polyacetylenes of diene-diyne types and their glycosides | 1 | Acetylatractylodinol |  | (Zhang, Zhao, Chang, Cao, Wang, Kang, Wang, Zhou, Huang, &Guo, 2021) |
|  | 2 | Atractylodin 2-[(1E,7E)-nona-1,7-dien-3,5-diynyl]furan |  | (Zhang, Zhao, Chang, Cao, Wang, Kang, Wang, Zhou, Huang, &Guo, 2021) |
|  | 3 | Diacetyl atractylodiol |  | (Yosioka, Tani, Hirose, & Kitagawa,1974) |
|  | 4 | (1Z)-atractylodinol |  | (Meng, Li, Dai, Ma, Zhang, Zhang, Li, & Wang, 2010) |
|  | 5 | (1Z)-acetylatractylodinol |  | (Resch, Heilmann, Steigel, & Bauer, 2001) |
|  | 6 | (1Z)-atractylodin  2-[(1Z,7E)-nona-1,7-dien-3,5-diynyl]furan |  | (Resch, Heilmann, Steigel, & Bauer, 2001) |
|  | 7 | 1,4-acetoxytetradeca-6,12-diene-8,10-diyne |  | (Sakurai, Sugawara, Saito, &Kano, 1994) |
|  | 8 | 1-(2-Furyl)-(1E,7E)-nonadiene-3,5-diyne- 9-ol |  | (Chen, Wu, Wang, & Gao, 2012) |
|  | 9 | 1-(2-Furyl)-(1E,7E)-nonadiene-3,5-diyne-9-al |  | (Chen, Wu, Wang, & Gao, 2012) |
|  | 10 | 1-(2-Furyl)-(1E,7Z)-nonadiene-3,5-diyne-9-ol |  | (Chen, Wu, Wang, & Gao, 2012) |
|  | 11 | 1-(2-Furyl)-(1E,7E)-nonadiene-3,5-diyne-9-yl benzoate |  | (Chen, Wu, Wang, & Gao, 2012) |
|  | 12 | 1-(2-Furyl)-(1E,7E)-nonadiene-3,5-diyne-9-yl 4- methylbenzoate |  | (Chen, Wu, Wang, & Gao, 2012) |
|  | 13 | 1-(2-Furyl)-(1E,7E)-nonadiene-3,5-diyne-9-acid |  | (Chen, Wu, Wang, & Gao, 2012) |
|  | 14 | (1Z)-atractylodinol 1-(2-Furyl)-(1Z,7E)-nonadiene-3,5- diyne-9-ol |  | (Resch, Heilmann, Steigel, & Bauer, 2001) |
|  | 15 | (1Z)-acetylatractylodinol 1-(2-Furyl)-(1Z,7E)-nonadiene- 3,5-diyne-9-yl acetate |  | (Resch, Heilmann, Steigel, & Bauer, 2001) |
|  | 16 | (2E,8E)-9-(furan-2-yl)nona-2,8-dien-4,6- diyn-1-ol |  | (Zhang, Zhao, Chang, Cao, Wang, Kang, Wang, Zhou, Huang, &Guo, 2021) |
|  | 17 | (2E,8E)-decadiene-4,6-diyne-1,10-diol 1-O-β-D- glucopyranoside |  | (Kitajima, Kamoshita, Ishikawa, Takano, Fukuda, Isoda, & Ida, 2003b) |
|  | 18 | (2E,8Z)-deca-2,8-diene-4,6-diyne-1,10-diol-1-O-β-D-  glucopyranoside |  | (Xu, 2017) |
|  | 19 | (2E,8E)-deca-2,8-diene-4,6-diyne-1,10-diol-1-O-β-D- apiofuranosyl-(1→6)-β-D-glucopyranoside |  | (Xu, 2017) |
|  | 20 | (2E,8Z)-deca-2,8-diene-4,6-diyne-1,10-diol-1-O-β-D- apiofuranosyl-(1→6)-β-D-glucopyranoside |  | (Xu, 2017) |
|  | 21 | (2Z,8E)-deca-2,8-diene-4,6-diyne-1,10-diol-1-O-β-D- glucopyranoside |  | (Xu, 2017) |
|  | 22 | (2E,8E,10R)-tridecatriene-4,6-diyne-1,10,11,12,13- pentol-10-O-β-D-glucopyranoside |  | (Xu, 2017) |
|  | 23 | (2E,8E,10R)-tridecane-2,8-diene-4,6-diyne-1,10, 13-di-O-β-D-xylopyranoside |  | (Xu, 2017) |
|  | 24 | (2E,8E,12S)-tetradecadiene-4,6-diyne-1,10,14-triol-1-O-β-D-apiofuranosyl-(1→6)-β-D-glucopyranoside |  | (Xu, 2017) |
|  | 25 | (3R,8E,10E)-tetradecadiene-4,6-diyne-3,12,14-triol-3-O-β-D-glucopyranoside |  | (Xu, 2017) |
|  | 26 | (4E,10E)-dodeca-4,10-dien-6,8-diyne-1,3-diyl diacetate |  | (Meng, Li, Dai, Ma, Zhang, Zhang, Li, & Wang, 2010) |
|  | 27 | (6E,12E)-tetradecadiene-8,10-diyne-1,3-diol |  | (Meng, Li, Dai, Ma, Zhang, Zhang, Li, & Wang, 2010) |
|  | 28 | (6E,12Z)-tetradecadiene-8,10-diyne-1,3-diol |  | (Meng, Li, Dai, Ma, Zhang, Zhang, Li, Wang, 2011) |
|  | 29 | (6Z,12Z)-tetradecadiene-8,10-diyne-1,3-diol |  | (Meng, Li, Dai, Ma, Zhang, Zhang, Li, Wang, 2011) |
|  | 30 | (6E,12E)-tetradecadiene-8,10-diyne-1,3-diol diacetate |  | (Meng, Li, Dai, Ma, Zhang, Zhang, Li, & Wang, 2010) |
|  | 31 | (6E,12E)-3-acetoxytetradeca-6,12-dien-8,10-diyn-1-ol |  | (Meng, Li, Dai, Ma, Zhang, Zhang, Li, & Wang, 2010) |
|  | 32 | (6E,12E)-1-acetoxytetradeca-6,12-dien-8,10-diyn-3-ol |  | (Meng, Li, Dai, Ma, Zhang, Zhang, Li, & Wang, 2010) |
|  | 33 | (6E,12E)-aetradecadiene-8,10-diyne-1,3-diol |  | (Meng, Li, Dai, Ma, Zhang, Zhang, Li, & Wang, 2010) |
|  | 34 | (8R,9S)-2E,10Z-tridecadiene-4,6-diyne-8,9,12,13-triol-9- O-β-D-glucopyranoside |  | (Xu, 2017) |
|  | 35 | (8R,9S)-2E,10Z-tridecadiene-4,6-diyne-8,9,12,13-triol-8-O-β-D-glucopyranoside |  | (Xu, 2017) |
|  | 36 | (8R,9S)-2E,10Z-tridecadiene-4,6-diyne-8,9,12,13-triol |  | (Xu, 2017) |
|  | 37 | 9-nor-atractylodin |  | (Chen, Wu, Wang, & Gao, 2012) |

Table 5. The components of triene-diyne types of atractylodes polyacetylenes and their glycosides.

| Chemical compounds | Number | Compound name | Structure | Ref. |
| --- | --- | --- | --- | --- |
| Triene-diyne types of atractylodes polyacetylenes and their glycosides | 1 | Erythro-(1,3Z,11E)-tridecatriene-7,9-diyne-5,6-diyl- diacetate |  | (Resch, Heilmann, Steigel, & Bauer, 2001) |
|  | 2 | Erythro-(1,5E,11E)-tridecatriene-7,9-diyne-3,4-diacetate |  | (Lehner, Steigel, & Bauer, 1997) |
|  | 3 | Threo-(1,5E,11E)-tridecatriene-7,9-diyne-3,4-diacetate |  | (Lehner, Steigel, & Bauer, 1997) |
|  | 4 | (1,3Z,11E)-Tridecatriene-7,9-diyne-5-hydroxyl-6-O-β-D- glucopyranoside |  | (Ji, Feng, Xiao, Dong, Wang, Wang, & Zhao, 2010) |
|  | 5 | (1,5E,11E)-trideca-1,5,11-trien-7,9-diyne-3,4-diacetate |  | (Kim, 2016) |
|  | 6 | (1,5E,11E)-tridecatriene-7,9-diyne-3,4-diacetate |  | (Resch, Heilmann, Steigel, & Bauer, 2001) |
|  | 7 | (2Z,4E,10E)-trideca-2,4,10-trien-6,8-diynyl acetate |  | (Kim, 2016) |
|  | 8 | (2E,8E,10E,12R)-tridecatriene-4,6-diyne-1,12,13-triol-1,12-di-O-β-D-glucopyranoside |  | (Xu, 2017) |
|  | 9 | (2E,8E,10E,12R)-tridecatriene-4,6-diyne-1,12,13-triol-1- O-β-D-apiofuranosyl-(1→6)-β-D-glucopyranoside |  | (Xu, 2017) |
|  | 10 | (2E,8E,10E,12R)-tetradeca-2,8,10-triene-4,6-diyne- 1,2,14-triol-1-O-β-D-apiofuranosyl-(1→6)-β-D- Glucopyranoside |  | (Xu, 2017) |
|  | 11 | (2E,8E,10E,12R)-tetradeca-2,8,10-triene-4,6-diyne- 1,12,14-triol-1-O-β-D-glucopyranoside |  | (Xu, 2017) |
|  | 12 | (3S,4E,6E,12E)-1-isovaleryloxy-tetradeca- 4,6,12-triene-8,10-diyne-3,14-diol |  | (Kim, 2016) |
|  | 13 | (3Z,5E,11E)-tridecatriene-7,9-diynyl-1-O-(E)-ferulate |  | (Resch, Heilmann, Steigel, & Bauer, 2001) |
|  | 14 | (3E,5E,11E)-tridecatriene-7,9-diyne-l,2-diacetate |  | (Lehner, Steigel, & Bauer, 1997) |
|  | 15 | (3Z,5E,11E)-tridecatriene-7,9-diyne-1,2-diacetate |  | (Resch, Heilmann, Steigel, & Bauer, 2001) |
|  | 16 | (3E,5Z,11E)-tridecatriene-7,9-diyne-1,2-diacetate |  | (Lehner, Steigel, & Bauer, 1997) |
|  | 17 | (3E,5Z,11E)-tridecatriene-7,9-diyne-1,2-diyl diacetate |  | (Resch, Heilmann, Steigel, & Bauer, 2001) |
|  | 18 | (4E,6E,12E)-tetradecatriene-8,10-diyne-1,3-diyl diacetate |  | (Yahara, Higashi, Iwaki, Nohara, Marubayashi, Ueda, Kohda, Goto, Izumi, Nuno, Katsuki, Isoda, & Satake, 1989) |
|  | 19 | (4E,6E,12E)-Tetradecatriene-8,10-diyne-1,3-diol |  | (Resch, Heilmann, Steigel, & Bauer, 2001) |
|  | 20 | (4E,6E,12E)-Tetradecatrien-8,10-diyn-1-ol |  | (Resch, Heilmann, Steigel, & Bauer, 2001) |
|  | 21 | (4E,6E,12E)-3-isovaleryloxy-tetradeca-4,6,12-triene-8,10- diyne-1,14-diol |  | (Resch, Heilmann, Steigel, & Bauer, 2001) |
|  | 22 | (4E,6E,12E)-tetradeca-4,6,12-trien-8,10-diyne-1,3,14- triol |  | (Kim, 2016) |
|  | 23 | (4E,6E,12E)-tetradecadiene-triene-8,10-diol |  | (Meng, Li, Dai, Ma, Zhang, Zhang, Li, & Wang, 2010) |
|  | 24 | (4E,6E,12E)-1-acetoxy-3-isovaleryloxy-4,6,12-trien-8,10- diyn-14-ol |  | (Kim, 2016) |
|  | 25 | (4E,6E,12E)-1-acetoxy-3-(2-methylbutyryloxy)-4,6,12- trien-8,10-diyn-14-ol |  | (Kim, 2016) |
|  | 26 | (5E,11E)-trideca-1,5,11- trien-7,9-diyne-3,4- diyldiacetate |  | (Kim, 2016) |
|  | 27 | (8S,9R)-2E,10Z,12-tridecadiene-4,6-diyne-1,8,9-triol-8-O-β-D-glucopyranoside |  | (Xu, 2017) |
|  | 28 | (8S,9R)-2E,10Z,12-tridecadiene-4,6-diyne-1,8,9-triol-8-O-β-D-apiofuranosyl-(1→6)-β-D-glucopyranoside |  | (Xu, 2017) |
|  | 29 | (8S,9R)-2E,10Z,12-tridecadiene-4,6-diyne-1,8,9-triol-9-O-β-D-glucopyranoside |  | (Xu, 2017) |
|  | 30 | (8S,9R)-2E,10Z,12-tridecadiene-4,6-diyne-1,8,9-triol-9-O-β-D-apiofuranosyl-(1→6) -β-D-glucopyranoside |  | (Xu, 2017) |
|  | 31 | (8E,10E)-tetradecadiene-4,6-diyne-3,12,14-triol-3-O-β-D- glucopyranoside |  | (Xu, 2017) |
|  | 32 | (10R,11R)-2R,8E,12-tridecatriene-4,6-diyne-1,10,11- triol-10-O-β-D-glucopyranoside |  | (Xu, 2017) |
|  | 33 | (10R,11R)-2E,8E,12-tridecatriene-4,6-diyne-1,10,11-triol- 1-O-β-D-glucopyranoside |  | (Xu, 2017) |
|  | 34 | (10R,11R)-2E,8E,12-tridecatriene-4,6-diyne-1,10,11-triol- 1,10-O-di-β-D-glucopyranoside |  | (Xu, 2017) |
|  | 35 | (10R,11R)-2E,8E,12-tridecatriene-4,6-diyne-1,10,11-triol- 10-O-β-D- apiofuranosyl-(1→6) -β-D-glucopyranoside |  | (Xu, 2017) |
|  | 36 | (10R,11S)-2R,8E,12-tridecatriene-4,6-diyne-1,10,11-triol- 10-O-β-D-glucopyranoside |  | (Xu, 2017) |
|  | 37 | (10R,11S)-2E,8E,12-tridecatriene-4,6-diyne-1,10,11-triol- 10-O-β-D- apiofuranosyl-(1→6)-β-D-glucopyranoside |  | (Xu, 2017) |
|  | 38 | (10S,11R)-2E,8E,12-tridecatriene-4,6-diyne-1,10,11-triol- 10-O-β-D-glucopyranoside |  | (Xu, 2017) |
|  | 39 | 12,14- diacetate- 2E,8E,10E- trien- 4,6-diyn-1-ol |  | (Kim, 2016) |

Table 6. The components of monoene-diyne types of AMP and their glycosides.

| Chemical compounds | Number | Compound name | Structure | Ref. |
| --- | --- | --- | --- | --- |
| Monoene-diyne types of aractylodes  polyacetylenes and their  glycosides | 1 | (E)-deca-2-ene-4,6-diyne-1,10-diol-1-O-β-D- glucopyranoside |  | (Xu, 2017) |
|  | 2 | (E)-deca-2-ene-4,6-diyne-1,10-dio1-1-O-β-D- apiofuranosyl-(1→6)-β-D-glucopyranoside |  | (Xu, 2017) |
|  | 3 | 1-(2-Furyl)-(7E)-nonene-3,5-diyne-1,2-diacetate |  | (Lehner, Steigel, & Bauer, 1997) |
|  | 4 | (2E)-decene-4,6-diyne-1,8-diol8-O-β-D-apiofuranosyl-(1→6)-β-D-glucopyranoside |  | (Kitajima, Kamoshita, Ishikawa, Takano, Fukuda, Isoda, & Ida, 2003b) |
|  | 5 | (2E,8S)-decane-4,6-diyne-1,8-diol-8-O-β-D- glucopyranoside |  | (Xu, 2017) |
|  | 6 | (2E,8R)-decene-4,6-diyne-1,8-diol-1-O-β-D-apiofuranosyl- (1→6)-β-D-glucopyranoside |  | (Xu, 2017) |
|  | 7 | (2E,8R)-decane-4,6-diyne-1,8-diol-O-di-β-D- glucopyranoside |  | (Xu, 2017) |
|  | 8 | (2E,10S)-tridecane-2-ene-4,6-diyne-1,10,13-triol-1,13-di- O-β-D-xylopyranoside |  | (Xu, 2017) |
|  | 9 | (2E,10S)-tridecatriene-4,6-diyne-1,12,13-triol-13-O-β-D- glucopyranoside |  | (Xu, 2017) |
|  | 10 | (2E,10S)-tridecatriene-4,6-diyne-1,12,13-triol-10-O-β-D- glucopyranoside |  | (Xu, 2017) |
|  | 11 | (2E,10S)-tetradecadiene-4,6-diyne-1,10,14-triol-10-O-β-D-apiofuranosyl-(1→6)-β-D-glucopyranoside |  | (Xu, 2017) |
|  | 12 | (8R,9R)-8,9-dihydroxylatractylodinol-8-O-β-D- glucopyranoside |  | (Xu, 2017) |
|  | 13 | (8R,9R)-8,9-dihydroxylatractylodinol-9-O-β-D- glucopyranoside |  | (Xu, 2017) |

Table 7. The components of other alkynes and their glycosides.

| Chemical compounds | Number | Compound name | Structure | Ref. |
| --- | --- | --- | --- | --- |
| Other alkynes and their glycosides | 1 | Bis (5-[(1E,7E)-nona-1,7-dien-3,5-diyn-1-yl)]furan-2-yl) methane |  | (Chen, Wu, Wang, & Gao, 2012) |
|  | 2 | 2-[(2E)-3,7-dimethyl-2,6-octadienyl]-6-methyl-2,5- cyclohexadiene-1,4-dione |  | (Resch, Steigel, Chen, & Bauer, 1998) |
|  | 3 | 2-[(2′E)-3′,7′-dimethyl-2′,6′-octadienyl]-4-methoxy-6- methylphenol |  | (Resch, Steigel, Chen, & Bauer, 1998) |
|  | 4 | 2,8-dimethyl-6-hydroxy-2-(4-methyl-3-pentenyl)-2H- chromene |  | (Resch, Steigel, Chen, & Bauer, 1998) |
|  | 5 | (8S)-decane-4,6-diyne-1,8-diol-O-β-D-glucopyranoside |  | (Xu, 2017) |
|  | 6 | (10R)-atracthioenyneside A |  | (Xu, 2017) |
|  | 7 | (10R)-atracthioenyneside B |  | (Xu, 2017) |
|  | 8 | (10S,11R)-atracthioenyneside C |  | (Xu, 2017) |
|  | 9 | (10S,11R)-atracthioenyneside D |  | (Xu, 2017) |
|  | 10 | (10R,11S)-atracthioenyneside E |  | (Xu, 2017) |

Table 8. The components of aromatic glycosides.

| Chemical compounds | Number | Compound name | Structure | Ref. |
| --- | --- | --- | --- | --- |
| Aromatic glycosides | 1 | Chlorogenic acid (1S,3R,4R,5R)-3-[3-(3,4-dihydroxyphenyl) prop-2-enoyloxy]-1,4,5- trihydroxycyclohexanecarboxylic acid |  | (Feng, Xu, Wang, Du, Zhang, Yang, Jiang, & Zhang, 2018) |
|  | 2 | Icariside F2 |  | (Kitajima, Kamoshita, Ishikawa, Takano, Fukuda, Isoda, & Ida, 2003b) |
|  | 3 | Icariside D1 |  | (Kitajima, Kamoshita, Ishikawa, Takano, Fukuda, Isoda, & Ida, 2003b) |
|  | 4 | Phenethyl *α*-L-rhamnopyranosyl-(1→6)-*β*-D-glucopyranoside |  | (Kitajima, Kamoshita, Ishikawa, Takano, Fukuda, Isoda, & Ida, 2003b) |
|  | 5 | P-hydroxybenzoic acid-4-O-*β*-D-glucopyranosyl-(1→3)-*α*-L-rhamnopyranoside |  | (Xu, Yang, Feng, Jiang, & Zhang, 2016b) |
|  | 6 | Seguinoside B,4-hydroxyphenyl 1-O-*β*-D - apiopyranosyl-(1→6)-*β*-D-glucopyranoside |  | (Kitajima, Kamoshita, Ishikawa, Takano, Fukuda, Isoda, & Ida, 2003b) |
|  | 7 | Scopoletin-D-xylopyranosyl-(1→6)-D-glucopyranoside |  | (Kitajima, Kamoshita, Ishikawa, Takano, Fukuda, Isoda, & Ida, 2003b) |
|  | 8 | Vanillic acid-4-O-*β*-D-glucopyranosyl-(1→3)-*α*-L-rhamnopyranoside |  | (Xu, Yang, Feng, Jiang, & Zhang, 2016b) |
|  | 9 | 1,3-di-O-caffeoylquinic acid |  | (Feng, Xu, Wang, Du, Zhang, Yang, Jiang, & Zhang, 2018) |
|  | 10 | 4-hydroxy-3-methoxyphenol-*β*-D-glucopyranoside |  | (Kitajima, Kamoshita, Ishikawa, Takano, Fukuda, Isoda, & Ida, 2003b) |
|  | 11 | 4-[(1E)-3-Hydroxyprop-1-en-1-yl]-2,6-dimethoxyphenyl-*β*-D-glucopyranoside |  | (Kitajima, Kamoshita, Ishikawa, Takano, Fukuda, Isoda, & Ida, 2003b) |
|  | 12 | 4-hydroxy-3-methoxyphenol-*β*-D-apiopyranosyl-(1→6)-D-glucopyranoside |  | (Kitajima, Kamoshita, Ishikawa, Takano, Fukuda, Isoda, & Ida, 2003b) |
|  | 13 | 4-hydroxy-3-methoxyphenyl-*β*-xylopyranosyl-(1→6)-*β*-glucopyranoside |  | (Kitajima, Kamoshita, Ishikawa, Takano, Fukuda, Isoda, & Ida, 2003b) |
|  | 14 | 5-O-feruloylquinic acid (1R,3R,4S,5R)-1,3,4-trihydroxy-5-[(2E)-3-(4-hydroxy-3- methoxyphenyl)-prop-2-enoyl]-oxycyclohexane-1-carboxylic acid |  | (Feng, Xu, Wang, Du, Zhang, Yang, Jiang, & Zhang, 2018) |

Table 9. The components of acyl sugar compounds.

| Chemical compounds | Number | Compound name | Structure | Ref. |
| --- | --- | --- | --- | --- |
| Acyl sugar compounds | 1 | 2,1′,3′,6′-tetra (3-methylbutanoyl) sucrose |  | (Tanaka & Ina, 2009) |
|  | 2 | 2,4,3′,4′-tetra (3-methylbutanoyl) sucrose |  | (Tanaka & Ina, 2009) |
|  | 3 | 2,6,3′,4′-tetra (3-methylbutanoyl) sucrose |  | (Tanaka & Ina, 2009) |
|  | 4 | 2,4,3′,6′-tetra (3-methylbutanoyl) sucrose |  | (Tanaka & Ina, 2009) |
|  | 5 | 2,6,3′,6′-tetra (3-methylbutanoyl) sucrose |  | (Tanaka & Ina, 2009) |
|  | 6 | 3′,4′,6′-tris (3-methylbutanoyl)-1’-(2-methylbutanoyl) sucrose |  | (Tanaka & Ina, 2009) |

Table 10. The components of other compounds.

| Chemical compounds | No. | Compound name | Structure | Ref. |
| --- | --- | --- | --- | --- |
| Other compounds | 1 | Atractylochromene [2,8-dimethyl-2-(4- methyl-3-penten-1-yl)-2H-chromen-6-ol] |  | (Resch, Steigel, Chen, & Bauer, 1998) |
|  | 2 | Diethyl phthalate |  | (Yang, 2007) |
|  | 3 | D-tryptophan [(R)-*α*-amino-3- indolepropionic Acid] |  | (Kitajima, Kamoshita, Ishikawa, Takano, Fukuda, Isoda, & Ida, 2003b) |
|  | 4 | Isopropyl *β*-D-apiofuranosyl-(1→6)-*β*-D- glucopyranoside |  | (Kitajima, Kamoshita, Ishikawa, Takano, Fukuda, Isoda, & Ida, 2003b) |
|  | 5 | L-phenylalanine |  | (Kitajima, Kamoshita, Ishikawa, Takano, Fukuda, Isoda, & Ida, 2003b) |
|  | 6 | Osthol |  | (Resch, Steigel, Chen, & Bauer, 1998) |
|  | 7 | Palmitic acid |  | (Resch, Heilmann, Steigel, & Bauer, 2001) |
|  | 8 | Trans-2-hydroxyisoxypropyl-3-hydroxy-7- isopentene-2,3-dihydrobenzofuran-5- carboxylic acid |  | (Duan, Wang, Qian, Su, & Tang, 2008) |
|  | 9 | Vanillic acid (4-Hydroxy-3-methoxy- benzoic acid) |  | (Liang, Li,  & Li, 2002) |
|  | 10 | Wogonin [5,7-dihydroxy-8- methoxyflavone] |  | (Li, Wang, Li, & liang, 2002) |
|  | 11 | 2-[(2′E)-3′,7′-dimethyl-2′,6′-octadienyl]-4- methoxy-6-methylphenol |  | (Resch, Heilmann, Steigel, & Bauer, 2001) |
|  | 12 | 3,5-dimethoxy-4-hydroxybenzoic acid |  | (Li, Wang, Li, & Liang, 2002) |
|  | 13 | 3-methoxy-4-hydroxybenzoic acid |  | (Li, Wang, Li, & Liang, 2002) |
|  | 14 | 3-methyl-2-butenyl *β*-D-apiofuranosyl-(1→6)-*β*-D-glucopyranoside |  | (Kitajima, Kamoshita, Ishikawa, Takano, Fukuda, Isoda, & Ida, 2003b) |
|  | 15 | 5-hydroxymethyl furaldehyde |  | (Meng, Li, Dai, Ma, Zhang, Zhang, Li, & Wang, 2010) |
|  | 16 | ALP-1 |  | (Qin, Wang, Zhuang, Meng, Zhang, Huang, & Lv, 2019) |
|  | 17 | ALP-3 |  | (Qin, Wang, Zhuang, Meng, Zhang, Huang, & Lv, 2019) |
|  | 18 | ACPS |  | (Xu, Chen, Liu, Wu, Dong, & Zhou, 2016c) |
|  | 19 | FOS |  | (Zhuang, Qin, Wang, Zhang, Liu, Ding, & Lv,2019) |
|  | 20 | Oligo-1 |  | (Taguchi, Kiyohara, Matsumoto, & Yamada, 2004) |
|  | 21 | Oligo-2 |  | (Taguchi, Kiyohara, Matsumoto, & Yamada, 2004) |
|  | 22 | Oligo-3 |  | (Taguchi, Kiyohara, Matsumoto, & Yamada, 2004) |
|  | 23 | Oligo-4 |  | (Taguchi, Kiyohara, Matsumoto, & Yamada, 2004) |
|  | 24 | Oligo-5 |  | (Taguchi, Kiyohara, Matsumoto, & Yamada, 2004) |
|  | 25 | Oligo-6 |  | (Taguchi, Kiyohara, Matsumoto, & Yamada, 2004) |
|  | 26 | Oligo-7 |  | (Taguchi, Kiyohara, Matsumoto, & Yamada, 2004) |
|  | 27 | Oligo-8 |  | (Taguchi, Kiyohara, Matsumoto, & Yamada, 2004) |

Table 11. The main pharmacological effects of Baizhu

| Pharmacology | Therapeutic ingredients | Outcome measure | The dose of medicine | Administration routes | Biological activity | Refer |
| --- | --- | --- | --- | --- | --- | --- |
| Diuretic effect | Decoction and liquid infusion of Baizhu | Renal tubular | 1.0 g/kg in rats; 1.0 g/kg in rabbits; 0.05-0.25 g/kg in dogs, 1.0 to 3.0 g/kg | Intravenously; Intragastric administration | Inhibits renal tubule reabsorption and promotes urine excretion. | (Chen, & Zhang 1961) |
|  | *β*-eucalyptus alcohol of Baizhu | (Na^+^-K^+^)-ATP enzyme | 1.6×10^-4^ mol/L Inhibition rate of 50%; 2.7×10^-3^ mol/L Inhibition rate of 85% | Not mentioned | Inhibitory phosphorylation of (Na^+^-K^+^)-ATP enzyme increases urine output and Na^+^ excretion. | (Zhou, & Zhou, 1996) |
|  | Atractylon | (Na^+^-K^+^)-ATP enzyme | 8.9 ×10^-6^ mol/L | In vitro | Inhibits (Na^+^-K^+^)-ATP enzyme activity. | (Satoh, Nagai, Ushiyama, & Kano, 1996) |
|  | Baizhu decoction | Peritoneal hole | High dose 60 g/d in mus musculus | Intragastric administration | Opening large peritoneal foramen, increasing the number of opening peritoneal foramen and increasing the average distribution density of peritoneal foramen can control the peritoneal foramen to realize the effect of eliminating ascites and indirectly producing diuretic effect. | (Li, Lv, Shi, Shen, & Chen, 1996b) |
|  | Quansheng Baizhu San Jiawei Granules | The increase of K^+^ content in urine had no obvious effect on Na^+^ in urine | 11.8, 23.6, 47.2 g/kg | Intragastric administration | The content of K^+^ in urine of rats was increased, but there was no obvious effect on Na^+^ in urine, and the capillary permeability was significantly promoted. | (Tao, Chen, & Zhou, 2002) |
| Antidiuretic effect | Baizhu decoction and volatile oil | No description | Low dose 10 g/d, medium dose 30 g/d Baizhu decoction in mus musculus; High-dose Baizhu decoction (7.20 g/kg) and high-dose Baizhu volatile oil (6.22×10^-2^ g/kg) in rat. | Intragastric administration | The medium and high dose decoction of Baizhu had certain antidiuretic effect on normal rat. | (Chen, Sun, Ran, Yuan, & Dou, 2016) |
| Anti-inflammatory effects | Atractylodes lactones | Acute inflammation | Not mentioned | Not mentioned | It has an inhibitory effect on acute inflammation in animals. | (Zhou, & Zhou, 1996) |
|  | Baizhu water decoction | TNF-*α* | Contains 0.06 g of crude drug per milliliter | Apply ointment | The content of TNF-*α* in serum of inflammatory mice was decreased. | (Huang, Ding, Sun, Li Chen, & Wang, 2005b) |
|  | Atractylodes lactone I | TLR4 receptor | Low dose 30 mg/kg, medium dose 100 mg/kg, high dose 300 mg/kg | Intragastric administration | The inflammatory swelling degree of auricle induced by xylene was reduced, and the increase of capillary permeability induced by acetic acid was significantly reduced. | (Li, & He, 2005) |
|  | Atractylodes lactone I | Inflammatory cytokine | 10, 20 and 40 mg/kg | Not mentioned | The levels of inflammatory cytokines were significantly reduced, which could improve sepsis syndrome and liver and kidney function. | (Wang, Xiao, Zhou, Zhang, Li, & He, 2016a) |
|  | AMP | Neutrophile granulocyte, ICAM-1 | 40 mg/kg | Intravenous administration | Reduces neutrophil infiltration and ICAM-1 expression in ischemic area. | (Wang, Feng, Liu, Li, & Qiu, 2009b) |
|  | Baizhu decoction | T cell | 1 g/ml 2ml | Intragastric administration | Causing the activation and proliferation of T cells and promoting the killing effect of T cells on inflammatory cytokines; IL-2 was up-regulated and IL-6 and IL-17 were down-regulated. | (Zhu, Qiu, Chen, Song, Chen, Ye, & Zhu, 2014) |
|  | Baizhu | COX-2, TNF-*α*mRNA, HO-1 | 100 mg/kg in 100 μL | Intragastric administration | Decrease the expression of COX-2 and TNF-*α* mRNA, and increase the expression of HO-1. | (Han, Park, Jeong, Han, Go, Park, Kim, Han, Kwon, & Hahm, 2017) |
|  | Alcohol extract of Baizhu | The degree of auricle swelling in mice | 0.25, 0.5, 1 g/(kg·d) | Intragastric administration | High and medium dose groups inhibited auricle swelling more significantly than low dose groups. | (Zhao, Pu, Zhou, Liang, Hu, Zhang, & Xu, 2016) |
|  | Alcohol extract of Baizhu | TNF-*α*、IL-1*β* and PGE2 | 0.25, 0.5, 1 g/(kg·d) | Intragastric administration | Inhibit the contents of TNF-*α*, IL-1*β* and PGE2 in serum and inflammatory tissue of AA rats. | (Zhao, Xu, Pu, Liang, Hu, & Wei, 2017a) |
| Antitumor effect | 1% Atractylodes lactone B oiling agent | Mouse sarcoma 180 | 100, 200 mg/kg | Intraperitoneal injection | Inhibition can reach more than 30%. | (Tang, Hao, Liu, Miao, Wei, & Wu, 1984) |
|  | Volatile oil of Baizhu | Mouse liver cancer H22, sarcoma S180 | 250 mg/ kg | Intragastric administration | The inhibitory effect was significant and dose-dependent. | (Wang, Liu, Shi, Zhang, & Yang, 2002b) |
|  | Atractylodes lactone Ⅰ | S180 tumor-bearing mice | 60 mg/kg | Intragastric administration | It has the effect of inhibiting tumor. | (Shen, He, Li, Sun, & Zhang, 2009) |
|  | Atractylodes lactone Ⅲ | S180 tumor-bearing mice | 60 mg/kg | Intragastric administration | It has the effect of inhibiting tumor. | (Shen, He, Li, Sun, & Zhang, 2009) |
|  | Water extract of Baizhu | S180 tumor-bearing mice | The concentration of crude drug in the low-dose group was 0.25 g/mL, in the medium-dose group was 0.5 g/mL, in the high-dose group was 1.0 g/mL，0.2 mL/10 g | Intragastric administration | The thymus index of S180 tumor bearing mice could be increased in medium and low dose groups. | (Zhu, Zheng, & Zhang, 2006) |
|  | Volatile oil of Baizhu | S180 tumor-bearing mice | 0.025, 0.05, 0.1 mL/kg | Intragastric administration | The expression of apoptosis-related gene bCL-2 was decreased in mice S180 sarcoma. | (Wang, Su,  & He, 2008b) |
|  | Volatile oil of Baizhu | SMMC772, HepG 2, A549, MCF-7 and HT29 | Not mentioned | Cell experiment | The volatile oil of Baizhu has a wide range of anti-tumor effects, especially on hepatocellular carcinoma cells. | (Lu, 2016) |
|  | Water extract of Baizhu | Meth A tumor | 250-500 mg/kg | Intragastric administration | Promotes delayed anaphylaxis specific to Meth A tumours in immunocompromised animals. | (Zhou, & Zhou, 1996) |
|  | Volatile oil of Baizhu | Macrophages | 15 g/kg | Intragastric administration | The number and activity of macrophages were greatly increased, and the function of macrophages was significantly enhanced, which contributed to the production of antibody-dependent cytotoxic effect mediated by macrophages. | (Nemere, 1995; Ninomiya, Yonemura, Matsumoto, Sugiyama, Kamata, Miwa, Miyazaki, & Shiku, 1991; Guan, Qu, Yang, Huang, & Sun, 2001) |
|  | Volatile oil of Baizhu | H22 liver cancer | 0. 025, 0.05, 0.1 ml /kg | Intragastric administration | It can significantly inhibit the lung metastasis rate of H22 liver cancer model, and significantly reduce the content of MMP-9 in serum of H22 liver cancer model mice. | (Wang, & Su, 2009) |
|  | Baizhu | HL-60 | 100, 125, 150, 200 μg/ml | Cell experiment | Increase the content of reactive oxygen species (ROS) to induce the apoptosis of HL-60. | (Huang, Chen, Yeh, & Huang, 2005a) |
|  | AMP | H22 liver cancer | 50, 100, 200 mg/ml | Intraperitoneal injection | The content of VEGF in serum was decreased, the content of IL-2 was increased, the expression of Bcl-2 gene was down-regulated, and the expression of p21 gene was up-regulated. | (Zhou, Su, & Song, 2015) |
|  | Baizhu | S180 tumor-bearing mice | 0.25, 0.5, 1.0 g/ml 0.4ml | Intragastric administration | It significantly increased the T cell transformation ability and promoted the secretion of interleukin 2(IL-2) in tumor bearing mice. | (Yao, Liu, & Lv, 2006) |
|  | Volatile oil of Baizhu | Macrophages | 15 g/kg | Intragastric administration | Enhance the body's non-specific immune function. | (Guan, Qu, Yang, Huang, & Sun, 2001) |
|  | Baizhu | Cell mutation | Not mentioned | Cell experiment | It has the effect of anti-cell mutation. | (Qiu, Tang, Yang, Shen, & Zheng, 1993) |
|  | Volatile oil of Baizhu | PG cells of lung cancer | Clinical equivalent dose of drug in rats, 3 ml/d | Intragastric administration | The ability of cell proliferation was reduced, the ability of cell adhesion and invasion were also significantly reduced. | (Zhao, Zhu, Zheng, Zhang, & Zhang, 2005) |
|  | AMP | PG cells of lung cancer | Clinical equivalent dose of drug in rats, 3 ml/d | Intragastric administration | The adhesion ability and invasion ability of the cells were also significantly reduced. | (Zhao, Zhu, Zheng, Zhang, & Zhang, 2005) |
|  | Atractylodes lactone Ⅰ | HL-60, P-338 cell | 30 μg/ml | Cell experiment | The apoptosis of cell lines HL-60 and P-338 was significantly induced. | (Wang, Chen, & Yang, 2002a) |
|  | AMP | Tumor weight and tumor volume ratio | 100 mg/kg, 200 mg/kg | Parenteral administration | Inhibits the proliferation of tumor cells and induces their apoptosis, which leads to the cessation of tumor cell growth. | (Zhang, Xu, Lin, Li, Zhang, & Lai, 2000; Cao, Zhang, Cong, Zhang, & Cai, 2009) |
|  | Baizhu | SGC-7901 cell | 40-640 mg/L | Cell experiment | Downregulation of c-myc gene expression inhibited the proliferation and enhanced immunity of SGC-7901 cells. | (Chen, Chen, Cui, Zheng, Xu, Chen, & Jia, 2007) |
|  | Atractylodes lactone II | B16 cells | 100 mM | Cell experiment | Inhibits the growth of cancer cells. | (Yan, Chou, Wang, Chu, Fong, & Yu, 2011) |
|  | Atractylodes lactone Ⅱ | CT26 cell | 50 μg/mL | Cell experiment | The inhibitory effect of cell proliferation was very obvious. | (Gao, Wang, Chen, Zai, & Bai, 2013) |
|  | Atractylodes lactone Ⅱ | Esophageal cancer ECA9706 cells | 50, 100, 200 μg/mL | Cell experiment | Inhibits cell proliferation. | (Gao, Wang, Yin, Zhang, & Chen, 2015) |
|  | Atractylodes lactone I | Melanoma cell A875 cells | 30, 60 μmol/L | Cell experiment | In the cell cycle, the proportion of cells in G2/M phase and S phase was significantly increased, while the proportion of cells in G0/G1 phase was decreased. Meanwhile, the proportion of cell apoptosis was significantly increased, which inhibited the tube formation of endothelial cells. | (Pan, & Song, 2015) |
|  | Atractylodes lactone I | PI3K/AKT pathway | 5, 10, 20, 40, 80, 160 μM | Cell experiment | The expression of CDK1 in SK-OV-3 and OVCAR-3 cells was down-regulated, and the cells were blocked in the G2/M phase. | (Long, Jia, Wang, Qing, Meng, & Wang, 2017) |
|  | Volatile oil of Baizhu | Cervical cancer HeLa, lung cancer A549 cells | 5, 10, 20, 50, 100, 200, 500, 1000 μg /mL | Cell experiment | In vitro experiments showed that the cell proliferation was significantly inhibited. | (Zhang, Shao, Wu, Shao, Zhao, & Xu, 2016) |
|  | Atractylodes lactone Ⅱ | Lovo cells from colorectal cancer | 150, 300 mg/L | Cell experiment | The growth and proliferation of Lovo cells in colorectal cancer were inhibited in a dose-dependent manner. | (Zhang, Zhang, Jiang, Long, Ruan, & Zhu, 2017) |
|  | Baizhu | Gastric cancer SGC-7901SP cells | Not mentioned | Drug serum was added to the cells | Inhibition is done by altering its cell cycle. | (Zhu, Yang, Guo, & Qian, 2019) |
|  | Atractylodes lactone I | Lung cancer A549 cells | 100 μM/L | Cell experiment | Inhibition of TLR4 and MyD88 protein expression decreased the invasion ability of these cells. | (Liu, Chu, Liu, & Wang, 2019) |
|  | Atractylodes lactone Ⅱ | Lovo cells from colorectal cancer | 100 mg/L | Cell experiment | The cleavage of PARP1 and caspase-3 promoted the apoptosis of LoVo cells in colorectal cancer. | (Zhang, Zhang, Jiang, Long, Ruan, & Zhu, 2017) |
|  | AMP | Glioma C6 cells | 10, 50, 100, 250, 500, 1000 μg/mL | Cell experiment | Apoptosis is induced by mitochondria-dependent pathways triggered by the destruction of mitochondrial membrane potential (MMP) and the release of cytochrome C. | (Li, Liu, Li, Ye, Huang, & Yuan, 2014) |
|  | Volatile oil of Baizhu | ECA cell | 50, 100, 150 mg/kg | Intraperitoneal injection | A large dose once. It can prolong the life of mice with tumor. | (Zhang, & Chen, 2006) |
|  | Shenling Baizhu powder | A tumor-burdened mice | 0. 6 g/d, 0. 5 mg/d | Intragastric administration | It may directly stimulate Th1 cells to increase the secretion of IL-2, and then induce killer cells to secrete IFN-*γ* and TNF-*α* to increase the body's immunity and play an anti-tumor role. | (Huang, Wang, Wang, & Chen, 2010) |
|  | Fuzheng Guben decoction | Hepatitis B virus surface antigen (HBsAg) (+) with various malignant tumors | Contains 12 g of scorched Baizhu | Oral administration | It plays a synergistic and attenuated role in malignant tumor chemotherapy. | (Duan, Liu, & Bao, 2010) |
|  | Qingshu Yi qi decoction | Lewis lung cancer mice | 10 ml/kg | Intragastric administration | Reduce the secretion of IL-1*β*, IL-6 and TNF-*α* by mouse macrophages. | (Chou, Kuo, Chen, Chen, Yeh, Kuo, & Chang, 2012) |
| The effect on gastrointestinal tract | Baizhu decoction | Cholinergic receptors | 0.1 mg/10g | Intragastric administration | Promotes gastrointestinal propulsion in mice. | (Ma, Fan, Chen, & Xing, 1995) |
|  | Water extract decoction of Baizhu | Ileal smooth muscle | 12.5%, 25%, 50%, 75%, 100% 1ml | In vitro studies | The larger dose of Baizhu decoction could significantly enhance the contraction of smooth muscle in ileum of guinea pigs. | (Ma, Fan, Chen, & Xing, 1995) |
|  | Baizhu | Acetylcholinesterase (AChE), substance P (SP) positive nerve | 2.5 g/kg | Intragastric administration | It can significantly increase the content of AChE positive nerve in gastric antrum and intramuscular plexus of jejunum and SP positive nerve in intramuscular plexus of gastric antrum, submucosa and intramuscular plexus of jejunum. | (Zhu, Zhuang, Xu, Zhang, Leng, & Chen, 2001) |
|  | Alcohol extract of Baizhu | Gastric mucosal cell | 0.25 %,0.5 %,1 % | cell experiment | Promote the proliferation of gastric mucosa cells and stimulate the secretion of pepsin. | (Zhu, Zhang, Huang, Chen, Xie, & Wang, 2003) |
|  | Baizhu | Propulsion velocity of gastrointestinal contents | 0.001, 0.01, 0.1 g/ml 0.2 ml | Intragastric administration | Accelerate the speed of gastrointestinal contents, and then can produce the function of invigorating stomach, benefiting spleen and regulating *qi.* | (Wu, Zhu, & Ma, 2005) |
|  | Volatile oil of Baizhu | Intestines and stomach | 25, 50, 100 mg/kg | Intragastric administration | Can promote gastrointestinal movement. | (Chen, Xia, Huang, Ge, & Xu, 2009c) |
|  | Baizhu decoction | Constipation mice | The ratio of fructus aurantii immaturus and Baizhu is 1:2.0.02, 0.04, 0.08 g/ml | Intragastric administration | The propulsive function of the small intestine was significantly enhanced with the increase of the dose. | (Chen, 2010) |
|  | Acetone extract of Baizhu | Rats with pyloric ligation | 300 mg/kg | Duodenal administration | It can increase the pH value of gastric juice, reduce the acidity of gastric juice, reduce the excretion of gastric acid and pepsin, and inhibit pepsin activity. | (Li, Liang, Yamahara, & Taniguchi, 1991) |
|  | Alcohol extract of Baizhu | Intestinal muscle tension | 0.2-4.0 mg/ml | In vitro experiment | With the increase of concentration, the inhibition of intestinal muscle contractility was strengthened. | (Yu, Fei, Li, Wang, & Shou, 2017) |
|  | Atractylodes lactone Ⅰ | The ileum in vitro | 28, 56 μmol/L, 28 mg/kg | In vitro experiment; Intragastric administration | The contractility of ileum in vitro was decreased and gastrointestinal motility was inhibited in normal rats. | (Zhang, Xu, & Lin, 1999) |
|  | 4, 15-epoxy hydroxyl groups atractylodes lactone | The ileum in vitro | 28, 56 μmol/L, 28 mg/kg | In vitro experiment; Intragastric administration | The contractility of ileum in vitro was decreased and gastrointestinal motility was inhibited in normal rats. | (Zhang, Xu, & Lin, 1999) |
|  | Atractylodes lactone Ⅲ | The ileum in vitro | 28, 56 μmol/L, 28 mg/kg | In vitro experiment; Intragastric administration | The contractility of ileum in vitro was decreased and gastrointestinal motility was inhibited in normal rats. | (Zhang, Xu, & Lin, 1999) |
|  | AMP | IEC-6 cells | 62.5, 125, 200, 500, 1000 mg/L | Cell experiment | The expression and distribution of villus protein in IEC-6 cells were up-regulated to promote its differentiation, thus promoting the repair of gastrointestinal mucosa. | (Wang, Li, Xu, & Chen, 2010b) |
|  | Methanol extract of Baizhu | IEC-6 cells | 50, 100, and 200 μg/mL | Cell experiment | The polyamine content of IEC-6 in small intestinal epithelial cells was significantly increased, the cell membrane was hyperpolarized, the concentration of free calcium ions [Ca^2+^] _Cyt_ in cytoplasm was increased, and the expression of Kv1.1 channel gene was increased, which stimulated cell migration. | (Song, Li, Chen, Wang, Cai, Liu, & Chen, 2014) |
|  | Atractylodes lactone Ⅰ | IEC-6 cells | 5, 10 μM | Cell experiment | The polyamine-mediated Ca^2+^ signaling pathway promotes the migration and proliferation of intestinal epithelial cells IEC-6. | (Song, Hou, Li, Yu, Li, Zhou, Huang, Cai, & Zhou, 2017) |
|  | Atractylodes lactone Ⅰ | Gastric mucosa | 10, 20, 40 mg/kg | Intragastric administration | It can increase the expression of heat shock protein 70(HSP70), decrease the content of IL-8, and decrease the expression of NF-*κ*B and COX-2 in chronic atrophic gastritis (CAG) model rats. | (Li, Yu, Gao, & Liu, 2016a) |
|  | AMP | IEC-6 cells | 25, 50, 100 mg/L | Cell experiment | The calcium ion level of IEC-6 in small intestinal epithelial cells under DFMO was increased, and cell migration and E-cadherin expression were promoted. | (Wu, Li, Zeng, Hu, Shi, Wang, & Chen, 2017) |
|  | AMP | Gastric mucosa | 0. 05 g/L 1 mL/100g | Intragastric administration | The gastric ulcer index, SOD activity, MDA content, Bcl-2 protein expression and Bax protein expression were significantly increased in rats with exercise stress ulcer model. | (Cao, & Bai, 2016) |
|  | Atractylodes lactone Ⅲ | Gastric mucosa | 10 mg/kg | Cell experiment | By activating tissue inhibitors of MMP-2 and MMP-9 in gastric ulcer tissue, MMP-2 and MMP-9 are inhibited to protect gastric mucosa. | (Wang, Chen, Wu, Chang, & Wang, 2010a) |
|  | AMP | Intestinal flora | 0.035, 0.105 g/kg | Intragastric administration | Anaerobic culture of intestinal flora can promote the digestion of reducing sugar by intestinal bacteria. | (Wang, Zhou, Wang, Peng, & Li, 2014) |
|  | Atractylodes lactone Ⅰ | Salivary amylase | 0.4, 0.8 mg/ml | In vitro experiment | Effect of enhancing salivary amylase activity. | (Hao, Sang, Li, Wang, & Jia, 2006) |
| Improves nervous system | AMP | Cerebral ischemia | Not mentioned | Not mentioned | It can reduce the cerebral edema after focal cerebral ischemia and reperfusion, reduce the damage of nerve cells, and improve the neurological function defect. | (Dong, 2015) |
|  | Baizhu | Focal cerebral ischemia/reperfusion | 2400 mg/kg/d | Intragastric administration | It can significantly improve the neural behavior of ischemia/reperfusion rats, increase the activities of SOD, GSH and CAT, and reduce the content of MDA in rat brain. | (Gao, 2017) |
|  | Biatractylenolide | Dementia rats | 0.1, 0.3, 1.0 mg/kg/d | Intragastric administration | It may be that the increase of Ach content in the brain region of learning and memory function can improve the mental impairment of animals with dementia. | (Feng, Wang, Lin, Zhou, Liu, & Yang, 2009) |
|  | Biatractylenolide | Mice model of memory impairment | 0.1, 0.3, 1.0 mg/kg/d | Intragastric administration | It can effectively reduce the activity of cholinesterase in the brain of the model mice induced by aluminum trichloride and improve the memory ability of the dementia mice. | (Liu, & Liao, 2006) |
|  | Baizhu | Brain aging mouse model | 700, 1050, 1400 mg/kg/d | Intragastric administration | It can improve the changes of gray type I synaptic structure in hippocampal CA3 region of mice induced by brain aging and improve the ability of learning and memory. | (Gao, & Yu, 2016a) |
|  | Baizhu | Brain aging mouse model | 700, 1050, 1400 mg/kg/d | Intragastric administration | The mechanism may be related to the up-regulation of Syn, PKC and CREB expression and the influence of synaptic plasticity. Enhance learning and memory function. | Gao, & Yu, 2016b) |
|  | Atractylodes lactone Ⅲ | Glutamate induced nerve cells | 10, 20 and 40 μM | Cell experiment | The anti-apoptotic properties may be related to the partial inhibition of the mediated Caspase signaling pathway, so it has a certain neuroprotective effect. | (Liu, Zhao, Ji, & Yu, 2014) |
|  | Atractylodes lactone Ⅰ | Mouse depression model | 5, 10, 20 mg/kg | Intragastric administration | Inhibition of NLRP3 inflammasome activation reduces IL-1*β* production. | (Gao, Zhu, Xi, Li, Shen, & Yang, 2018) |
|  | Baizhu | Plant nerve | Not mentioned | Not mentioned | Adjust the vegetative nerve, and then treat the patients with spleen deficiency similar to the related diseases of digestive tract dysfunction. | (Du, & Nie, 2004) |
|  | *β*-eucalyptus oil alcohol | Nervous system | 20 μM | Cell experiment | Combat neostigmine - induced neuromuscular disorders by reducing the regenerative release of ethylphthalcholine from repetitive stimuli. | (Nojima, Kimura, & Kimura, 1992) |
|  | AMP | Nerve cells | 0.025, 0.05, 0.1 g/L | Cell experiment | It may inhibit the apoptosis of nerve cells by decreasing the apoptotic genes and up-regulating the production of anti-apoptotic proteins. | (Hu, Xiang, Fu, Wen, He, & Hu, 2014) |
|  | AMP | Nervous system | 100, 200 mg/kg | Intragastric administration | It is possible to reduce the degree of secondary brain edema after traumatic brain injury by down-regulating the expression of nitric oxide synthase in the lesion area. | (Shi, Su, Yang, Lv, & Chen, 2014; Wang, Wei, Liu, Li, & Qiu, 2009) |
| Anticoagulant effect | Baizhu | Blood indicators | 0.54, 1.08, 2.16, 4.32 g/kg | Intragastric administration | Fbgc was decreased, PT and APTT were prolonged in rats. | (An, & Liang, 2010) |
| Affects the immune system | Baizhu | Immunosuppressive animal spleen cells | 1 g/ml | Cell experiment | It can improve the survival rate of spleen cells in vitro and prolong the life of lymphocytes. Increase the number of Th cells, improve the ratio of Th/TS, adjust the distribution disorder of T cell subsets, IL-2 level was significantly increased and IL-2R expression on the surface of T lymphocytes was increased. | (Yu, Zhang, Zhao, & Yu, 1994) |
|  | Volatile oil of Baizhu | Macrophages | 15 g/kg | Intragastric administration | Enhance the activity of macrophages and enhance the non-specific immune function of the body. | (Guan, Qu, Yang, Huang, & Sun, 2001) |
|  | AMP | Antibody | Not mentioned | Intragastric administration | Specific IgG class antibodies and non-specific cross antibodies were produced. | (Sun, Li, Hou, & Liu, 2008) |
|  | AMP | Splenic lymphocyte | 1.25, 2.5, 5.0, 10.0 μg /ml | Cell experiment | Promote the proliferation of splenic lymphocytes. | (Guo, Liang, Lou, & Zhang, 2012) |
|  | AMP | The mice serum | 100, 300, 600 μg | Abdominal subcutaneous injection | The levels of specific antibodies and antibody subclasses were significantly increased. | (Chai, Xie, Ge, Hou, Zhang, & Hu, 2013) |
|  | AMP | Kupffer cells | 25, 50, 100, 200, 400, 800 mg/L | Cell experiment | Enhance the phagocytosis of neutral red A540, increase the activity of ACP and the production of NO and TNF-*α*, do not increase the leakage of LDH in liver parenchymal cells, have NO effect on the activity of sALT and SGST, can activate the immune function of Kupffer cells. | (Jiao, Tang,  & Wang, 2013) |
|  | AMP | Lymphocytes from normal mice | 200 mg/(kg·bw) | Intragastric administration | It significantly increased the lymphocyte function of normal mice. | (Xiang, Xu, Cao, Qian, Tian, & Li, 2020) |
| Regulates uterine smooth muscle | Alcohol extract of Baizhu | Isolated uterus of non-pregnant mice | 0.5 g/ml 0.2 ml | In vitro experiment | All showed significant inhibitory effect on uterine excitatory contractions.  It also completely counteracts the tonotonic contractions caused by oxytocin in the pregnant uterus of guinea pigs *in vivo.* | (Zhou, Xu,  & Yang, 1993) |
|  | Baizhu | Uterine smooth muscle cells | 1, 2, 4 mg/ml | Cell experiment | The potassium channel current (BKCA) of uterine smooth muscle cells in late pregnancy and the uterine smooth muscle cells treated with IL-6 was excited, and the excitatory effect of BKCA on uterine smooth muscle cells treated with IL-6 was stronger than that on normal uterine smooth muscle cells, which was helpful to maintain the membrane potential and resting state of uterine smooth muscle during pregnancy to prevent premature labor. | (Zhang, Wang, Xu, & Zou, 2009) |
| Antioxidation and anti-aging effect | Baizhu decoction | Aging mice | 10 g/kg | Intragastric administration | It can significantly increase the activity of glutathione peroxidase (GSH-Px) in the whole blood of aged mice, reduce the content of MDA, remove the damage caused by free radicals in the body, avoid the damage of peroxy liposomes to cell structure and function, enhance the antioxidant ability of red blood cells, and protect red blood cells. | (Li, Guo, Mao, Xiong, & Tong, 1996a) |
|  | Baizhu | Red blood cell autooxidation hemolysis | 0.8 g/ml | In vitro experiment | It can improve SOD activity of red blood cells, inhibit the activity of monoamine oxidase B in mouse brain, resist the autooxidation hemolysis of red blood cells, and scavenging the active oxygen free radicals. | (Lv, Li, & Liu, 1996) |
|  | AMP | Asenile rat nerve cells | 0.056 g/mL 0.28 g/kg/d | Intragastric administration | It significantly increased the activity of SOD and GSH-Px, decreased the content of MDA, and reduced DNA damage. | (Ma, Zhang, Guo, Wei, & Ou, 2006) |
|  | Bran fried Baizhu, soil fried Baizhu, Baizhu decoction | Acute senescence model mice | 0.2 g/ml 0.2 ml/20g | Intragastric administration | Can reduce serum MDA. Decreased the Lf content in liver tissue. The activity of serum SOD and CAT was increased. | (Song, & Gu, 2007) |
|  | AMP | Classic animal model of aging | 100, 200 mg/kg | Intragastric administration | To reduce the concentration of MAO in the brain tissue, reduce the generation and accumulation of Lipo, so as to achieve the effect of anti-oxidation and anti-aging of the body. | (Shi, Su, Yang, Lv, & Chen, 2014) |
| Hypoglycemic effect | AMP-B | Diabetic rats with alloxan. | 50, 100, 200 mg/kg | Intragastric administration | Increasing thymus mass index and pancreatic mass index and inhibiting pancreatic atrophy in diabetic rats may reduce the damage of alloxan to pancreatic beta cells or improve the function of damaged beta cells. | (Shan, & Tian, 2003) |
|  | AMP | Type 2 diabetic mice | 30, 90, 300 mg/kg | Intragastric administration | Fasting blood glucose and plasma insulin levels were decreased and glucose tolerance was improved in diabetic mice. | (Li, Chen, Ji, Geng, & Lv, 2015b) |
|  | Atractylodes lactone Ⅰ and Atractylodes lactone Ⅱ | Mouse skeletal muscle C_2_C_12_ cells | 50 μg/ml | Cell experiment | Significantly increased GLUT-4 protein levels and promoted GLUT-4 translocalization to the plasma membrane, improving TNF-*α*-induced insulin resistance in C_2_C_12_ skeletal muscle cells. | (Chao, Huang, Lin, Chang, & Chang, 2016) |
| Analgesic action | Alcohol extract of Baizhu | Hot plate pain threshold of mice. The number of writhing reactions in mice induced by intraperitoneal injection of acetic acid | 0.25, 0.5, 1.0 g/kg/d | Intragastric administration | Possible analgesic activity by inhibiting the synthesis of prostaglandins in the peripheral nervous system. | (Zhao, Pu, Zhou, Liang, Hu, Zhang, & Xu, 2016) |
| Antibacterial activity | Baizhu | Epidermosum flocculent, Nucardia stellaris and Meningococcus | Not mentioned | Not mentioned | Have an inhibiting effect. | (Du, & Nie, 2004) |
| Protective effect of hepatic ischemia reperfusion injury (IRI) in rats | AMP | ALT, AST, ICAM-1 mRNA, IL-1, NF-*κ*B in liver tissue | 0.4 g/kg/d | Intragastric administration | The reduction of IRI in liver of rats after autologous liver transplantation may be related to the inhibition of NF-*κ*B expression, the interference of oxygen free radicals on the destruction of liver cell membrane, the promotion of enzyme degradation and the reduction of morphological damage. | (Jin, Zhang, Bao, Gu, Xu, Li, Li, Bo, & Liu, 2011) |
|  | Water extract of Baizhu | Mouse of liver injury degree, ALT and AST content increased | 30 g/kg | Intragastric administration | Have counterbalance function, make liver coefficient decrease, play the role of lowering fat and protecting liver. | (Peng, Gu, Jiang, Fang, & Yu, 2011) |
| Aromatase inhibition | Atractylodes lactones and its derivatives | Aromatase inhibition rate | 10 μM | In vitro experiment | Can be used as a potential aromatase inhibitor. | (Jiang, Shi, & Li, 2011) |
| Enhance the contractility of the heart muscle | AMP | The contractility of the heart muscle | 100 μg/ml | In vitro experiment | It can enhance the contractibility of myocardium, and its effect on isolated frog myocardium is similar to that of epinephrine, which can be inhibited by *β*-blockers, while M blockers have little effect on its effect. | (Ma, & Mei, 2007) |
|  | Biatractylenolide | Isolated atrial muscle | 1.19×10^-5^ mol·L | *In vitro* experiment | It significantly reduced the contractility of isolated guinea pig right atrium muscle and slowed down its heart rate, which reduced the normal stepping function of isolated guinea pig left atrium muscle.  There was no effect on the increase of left atrial muscle after rest. | (Pu, Wang, Huang, Xu, Lin, & Wu, 2000) |
| Protect liver function | Atractylon, *β*-eucalyptus alcohol | Mouse liver poisoning model | 0.01, 0.1, 1.0 mg/ml | Cell experiment | It has certain hepatoprotective effect. | (Kiso, Tohkin, & Hikino, 1983) |
|  | Atractylon | DNA damage and hepatocytotoxicity in rats | 0.01, 0.1, 1.0 mg/ml | Cell experiment | Have an inhibiting effect. | (Hwang, Tseng, Hsieh, Chou, Wang, & Chu, 1996) |
| Fall hematic fat | Baizhu 100% methanol extract | Blood lipid in mice | 30 g/kg | Intragastric administration | The body weight and serum TG level of mice were decreased. The serum HDL-C and HDL-C/TC levels were significantly increased, and the urine volume of the diseased mice was increased. | (Peng, Gu, Jiang, Fang, & Yu, 2011) |
| Improved acute kidney injury | AMP | The glomerular apoptotic cells | Contains 15 g of Baizhu | Oral administration | The expression of apoptotic genes and cells in glomerular apoptotic cells were decreased, and the expression of anti-apoptotic proteins was effectively up-regulated. | (Li, Tang, Gu, Li, Deng, Qin, Ma, Ye, & Yin, 2015a) |
| Inhibitory lipogenesis | Baizhu aqueous extract | 3T3-L1 adipocyte | 1-25 μg/ml | Cell experiment | The serum triglyceride level of drug administration group was significantly lower than that of model group and normal diet group.  It can inhibit adipocyte differentiation and adipogenesis by reducing adipokine and inhibiting p-Akt level. | (Kim, Kim, Oh, Lee, Sun, Choi, Kim, Bae, Kang, & Min, 2011) |
| Hypotensive effect | Baizhu powder | Spontaneous hypertensive cerebral apoplexy rats | 0.9 g/d | Oral administration | It has a certain effect of lowering blood pressure. | (Li, & Feng, 1997) |
| Improve bone disease | Atractylodes lactone Ⅲ and Atractylodes lactone Ⅰ | Gli promoter, mesenchymal stem cells | 1, 3, 10, 30, 100, 300 μg/ml | Cell experiment | Promoting the activity of the Gli promoter. It can also induce mesenchymal stem cells (MSC) to differentiate into chondrocytes. | (Li, Wei, Wang, Liu, Deng, Li, Zhou, Li, Zeng, & Chen, 2012) |

Table 12. The main application of Baizhu

| Disease |  | Drug | Refer |
| --- | --- | --- | --- |
| Gastrointestinal disease | Colonic slow transport constipation. | Baizhu decoction | (Ding, Ding, Zhang, Wang, & Zheng, 2005) |
|  | Painful diarrhea, intestinal inflammation, irritable bowel syndrome, which is dominated by diarrhea. | Baizhu Shaoyao powder | (Xu, Cai, Cao, Duan, Pei, Tu, Zhou, Xie, Sun, Zhao, Liu, Wang, & Shen, 2018; Zheng, Zhang, Qin, Cai, Cao, & Cai, 2015) |
|  | Functional dyspepsia, especially postprandial discomfort syndrome. | Xiangsha Liujunzi decoction | (Wang, Zhong, Kang, Dai, Lv, Bian, Chen, Zhang, Bian, Wang, Zhu, & Tang, 2016b) |
|  | Ulcerative functional dyspepsia. | Baizhu Shanzha decoction | (Wang, 2007) |
|  | Diarrhea in children. | Qiwei Baizhu powder | (Zhang, & Zhang, 2007) |
|  | Children's anorexia, peptic ulcer, gastrointestinal dysfunction, chronic enteritis, and simple dyspepsia after operation of malignant tumor of digestive system. | Shenling Baizhu powder | (Ge, & Wu, 2006; Sun, 2008; Xu, 2006; Peng, 2009; Hao, & Hao, 2004) |
| Cardiovascular disease | Vertigo caused by vertebrobasilar artery insufficiency, hypertension, hyperlipidemia, vertigo, meniere disease, headache, cerebral infarction, concussion, transient ischemic attack, essential hypertension. | Banxia Baizhu Tianma decoction | (Guo, Su, Wang, Luo, & Lai, 2017; Tan, Loh, Ng, Ch’ng, Asmawi, Ahmad, & Yam, 2018; Cai, Guo, Zhao, Chen, Zhao, & Chen, 2018; Yu, 2009; Li, Fu, Zhou, Gao, Li, & Tao, 2020; Zhang, & Hao, 2009; Li, 2009; Ning, & Zhong, 2009; Liu, & Zhong, 2006; Liu, Hu, Zhang, & Shen, 2007) |
| Immune system diseases | Cold, chronic bronchitis, allergic rhinitis and asthma. | Yupingfeng powder | (Du, Choi, Dong, Lau, & Tsim, 2014; Du, Zheng, Bi, Dong, Lin, & Tsim, 2015; Nikles, Monschein, Zou, Liu, He, Fan, Lu, Yu, Isaac, & Bauer, 2017) |
|  | Acute otitis media. | Shenling Baizhu powder combined with antibiotics | (Son, Kim, Song, & Kim, 2017) |
| Hepatic disease | Non-alcoholic fatty liver disease. | Qushi Huayu decoction | (Meng, Liu, Tang, Wang, Zheng, Tian, Yao, Liu, Peng, Zhao, Hu, & Feng, 2016) |
|  | Chronic toxic hepatitis, liver fibrosis, cirrhosis and alcoholic liver disease. | “*Liver* clearing” formula CGX | (Kim, Han, Lee, Lee, Son, Choi, Lee, Wang, & Son, 2013) |
|  | Cirrhosis ascites. | Qiwei Baizhu powder | (Li, 2005) |
|  | Cirrhosis. | Shenling Baizhu powder | (Wang, 2008) |
| Other diseases | Diabetes mellitus. | Qiwei Baizhu powder | (Zhang, Chen, Zhao, Ding, Hao, & Zou, 2006a) |
|  | Dema, obesity, chronic eczema in children, uremia combined with malnutrition, middle and late-stage ankylosing spondylitis. | Shenling Baizhu powder | (Wang, 2009; Lu, 2006; Dai, & Wang, 2007; Yu, & Qiu, 2007; Zuo, Liu, & Wang, 2007) |
|  | Chronic pelvic inflammation and blood stasis syndrome. | Li Chong decoction combined with moxibustion | (Chen, Liu, Deng, Yuan, Li, & Ren, 2015) |
|  | Insomnia. | Buzhong Yiqi decoction | (Wang, 2015) |

Table 13. Quality control of Baizhu

| Detection index | Method of sample extraction | Detection method | Detection wavelength | Characteristic | Refer |
| --- | --- | --- | --- | --- | --- |
| Atractylodes lactone Ⅰ; Atractylodes lactone Ⅲ | Ultrasonic extraction of methanol 30min | HPLC(Kromasil-ODS) | 220nm | This method is simple, sensitive and reliable, and can be used as a quantitative method for quality control of Baizhu. | (Li, Wen, Zhang,  Ge, & Wu, 2001) |
| atractylon; Atractylodes lactone Ⅰ; Atractylodes lactone Ⅲ | Percolation method | HPLC (Sh impack, CLC-ODS) | 220nm | The results showed that atractylon was transformed into Atractylodes lactone Ⅰ and Atractylodes lactone Ⅲ. The method is accurate, reliable, reproducible and without interference of components to be measured. | (Yu, Song, & Jia, 2005) |
| Components of volatile oil from processed and unprocessed Baizhu | Water distillation and alcohol extraction | TLC |  | The composition of Baizhu after processing is well reflected. Methods in accordance with the Chinese pharmacopoeia. | (Chen, Zhang, & Zhong, 2005) |
| Atractylodes lactone Ⅱ | Ultrasonic extraction of methanol 15min | HPLC(Kromasil-C18) | 276nm | This method is simple, sensitive and reliable, and can be used as one of the quantitative methods for quality control of Baizhu. | (Li, Wen, Cui, & Zhang, 2005) |
| Atractylodes lactone Ⅲ of fried Baizhu and coked Baizhu | Ultrasonic extraction of methanol 30min, water distillation, alcohol extraction | HPLC | 220nm | Taking the pharmacodynamic chemical components as the index components, it provides a reliable basis for industrial production. | (Fu, Yu, & Chen, 2006) |
| Atractylodes lactone Ⅲ of bran Fried Baizhu | Ultrasonic extraction of methanol 30min, water extracting, alcohol extraction | HPLC | 220 nm | The amount of water extract of Baizhu was significantly greater than 70% alcohol extract. Taking the pharmacodynamic chemical component as the index, high comprehensive evaluation and more objective. | (Fu, & Yang, 2007) |
| Atractylodes lactone Ⅲ | Ultrasonic extraction of methanol 30min | HPLC | 220 nm | The orthogonal table of the best factors was designed after the single factor investigation in advance. The thin pieces of Baizhu had a larger number of decoctions and the results were reliable. | (Zhu, Yin, & Han, 2007) |
| Atractylodes lactone Ⅱ | Ultrasonic extraction of methanol 20min | HPLC | 276nm | The content of Atractylodes lactone Ⅱ in raw Baizhu and processed Baizhu was 0. 086% and 0. 104% respectively. These results suggest that we should further study the structure, properties and pharmacological effects of other transformation products in order to better control the quality of Baizhu. The method is simple, accurate and reproducible. | (Hao, Sang, & Jia, 2008) |
| Atractylodes lactone Ⅰ | Ultrasonic extraction of methanol 30min | HPLC | 220nm | Simple, sensitive, reliable results. The average recovery of Atractylodes lactone I was 98.96% and RSD was 1.44%. The content limit of Atractylodes lactone I was set at 0.0731%, which was much higher than that reported in many literatures, which may be related to the harvest season and processing methods. | (Su, Wang, Wu, & Ruan, 2008) |
| Fingerprint of Baizhu | Ultrasonic extraction of methanol 30min | Reversed-phase high performance liquid chromatography (RP-HPLC) | 240nm | The established fingerprint detection method has good precision, reproducibility and stability. Several methods are compared in the selection of extraction method and mobile phase. | (Ye, Cheng, Chou, & Wang, 2009) |
| Atractylodes lactone Ⅰ, Ⅱ, Ⅲ of stir-fried Baizhu | Ultrasonic extraction of methanol 30min | HPLC | 220nm (Ⅰ, Ⅲ) ,276nm （Ⅱ） | The higher the temperature the longer the roasting time the lower the content. To establish a scientific and reasonable method for the determination of Baizhu. | (Duan, Li, Zhao, & Ma, 2009) |
| Atractylon, Atractylodes lactone I, Atractylodes lactone Ⅲ | Ultrasound extraction 30min | Microtubule liquid method | 220nm | The results of methodological investigation showed that all the indicators met the requirements of content determination. It can be used in the quality evaluation of multiple planting and cultivation to obtain high quality provenances. The results are stable has good operability and repeatability. | (Shou, Dai, Zhang, Li, & Yu, 2008) |
| Atractylodes lactone Ⅰ, Atractylodes lactone Ⅲ | Carbon dioxide supercritical fluid extraction | GC-MS |  | The method has good specificity high sensitivity the minimum detection limits of Atractylodes lactone Ⅰ and Atractylodes lactone Ⅲ were 16 and 25pg, respectively. | (Zhu, Chen, Li, & Zhang, 2009) |
| Baizhu | Ultrasound extraction (add n-hexane) | Ultraviolet detector | 254nm | All the established methods had satisfactory separation effect, good reproducibility, strong specificity, and no negative interference. | (Qu, Deng, Hao, & Zhao, 2008) |
| Fingerprint study | Ultrasound extraction 20min | HPLC (Symmetry C18) | 254nm | It is the first time for HPLC chromatography to be used in the identification and quality evaluation of Baizhu. | (Yu, Yang, Liu, Bai, Zhang, & Wang, 2006) |
| Fingerprint study | Ultrasound extraction 30min | HPLC (Dikma Kr omasil C18) | 242nm | The method was accurate, reliable and reproducibility, which provided a scientific basis for better control of the internal quality of Baizhu. | (Li, Ma, Xie, & Gong, 2007c) |
| Baizhu | Ultrasound extraction 30min | HPLC (Luna C18) | 230nm | The chemical pattern recognition method of Baizhu was established by using HPLC as a means of obtaining chemical data, but the obtained information can only reflect the quality of Baizhu from one side. | (Tian, Wu, Bi, Sun, Zhao, & Lv, 2003) |
| Atractylodes lactone Ⅲ, Atractylodes lactone Ⅱ, Atractylodes lactone Ⅰ | CO2 supercritical extraction; | HPLC; Serum pharmacochemistry | 190-400 | High pressure, high speed, high efficiency, high sensitivity. The pharmacodynamic substance basis and compound compatibility mechanisms were screened quickly. | (Cao, Zhu, Lin, Xiao, Yan, & Yu, 2007) |
| Baizhu decoction | Water decoction | Surface enhanced Raman spectroscopy; Uv-visible/near infrared spectrophotometer | 785nm | Provides an accurate, direct and rapid detection method. | (Chen, Feng, Lin, Chen, Li, & Lin, Huang, 2009b) |
| Volatile oil of Baizhu | Pellet method and diffuse reflectance method | Horizontal attenuated total reflectance (HATR)- Fourier Transform Infrared Spectrometer (FTIR) |  | It can be used to compare the different parts of Chinese medicinal materials directly and quickly to increase the accuracy of determination. | (Hong, Cheng, Cheng, & Li, 2007) |
| Atractylon | N-hexane soxhlet extraction and Steam distillation | Ultraviolet spectrophotometry | 252nm | The content of atractylon in Baizhu was determined by ultraviolet spectrophotometry, which provided a more reliable basis for ensuring the quality of the crude drug of Baizhu. | (Chen, Shao, & Lu, 1996) |
| Atractylon | Soxhlet extraction of n-hexane | TLC |  | The content of atractylon was about 32.44%. A very important experimental technique for rapid separation and qualitative analysis of small quantities of substances | (Zhang, Cao, Cong, Zhang, & Cai, 2011) |
| Fingerprint of Baizhu | Ultrasonic extraction 20min | High performance capillary electrophoresis (HPCE) | 200nm | The method is simple, rapid and does not require a large amount of chromatographic pure solvent. Because the sample solution is not pretreated, the components that can be identified are preserved to the maximum extent, and the characteristics of the atlas are enhanced. | (Zuo, & Ji, 2008) |
| Volatile oil of Baizhu | Steam distillation | Liquid Chromatography-Electrospray Ionization-Mass Spectrometry (LC/ESI/MS); Chemometric resolution Method (CRM) |  | Combined with the stoichiometric analytical method for the analysis of overlapping chromatographic peaks, compared with GC-MS alone, it can reflect the chemical composition of volatile oil in Baizhu more truly and comprehensively. | (She, Kuai, Xiong, Liang, & Tang, 2010) |
| Volatile oil of Baizhu | Carbon dioxide supercritical fluid extraction | GC-MS |  | Has a strong ability to separate, unique ability to identify unknown compounds, high sensitivity, it is one of the most powerful tools for the separation and detection of complex compounds. | (Qiu, Cui, Liu, & Zhang, 2002) |
| Lead, cadmium, copper, arsenic and mercury in Baizhu. |  | Atomic Absorption Spectrometry (AAS) |  | It has the advantages of low detection limit, high accuracy, good selectivity and fast analysis speed. This method is mainly applicable to the analysis of microscale and trace components in samples | (Zhang, Cao, Cong, Zhang, & Cai,  2011) |
| Atractylodes lactone Ⅰ, Ⅱ, Ⅲ | Ultrasonic extraction of methanol 30min | HPLC (Lichrospher C18), TLC | 220,270nm | In the experiment, the detection wavelength and extraction method were screened, and the optimal method was selected for the detection and identification of Baizhu. | (Qi, Yang, Li, Lv, & Wang, 2012) |
| Atractylodes lactone Ⅰ and Atractylodes lactone Ⅲ | Ultrasonic extraction of methanol 45 min | HPLC (ODS2 C18) | 222 nm | The method is simple, convenient and rapid, and can be used as a scientific basis for quality control and evaluation of Baizhu. It also provided a strong basis for the establishment of GDP and further processing of Baizhu. | (Li, Long, Cheng, Li, Huang, & Shuai, 2013) |
| Volatile oil of Baizhu | Supercritical CO_2_ extraction | HPLC | 220 nm | The analytical method is simple, stable and reliable. The quality standard of volatile oil from Baizhu was studied by content determination and fingerprint, which could reflect the quality of volatile oil more comprehensively and reliably | (Sun, Zhu, He, Feng, Zheng, & Mu, 2015) |
| 5-hydroxymethylfurfural, Atractylodes lactone III, Atractylodes lactone I, Atractylodes lactone II, Atractylodes lactone VI and biatractylenolide | Ultrasonic extraction of methanol 60 min | HPLC-PDA (Inertsil ODS-SP); UFLC-Q-TOF/MS | 235 nm | Traditional Chinese medicine (TCM) prepared slices and products were controlled by TCM fingerprint, and the authenticity and uniformity of TCM prepared slices were evaluated from the source. The method is simple, stable, reliable and reproducible. | (Sun, Wen, Cui, Lu, Li, & Shan, 2016) |
| Volatile oils from different processed products of Baizhu | Water steam distillation | GC- Flame ionization detector (FID) (HP-5 quartz capillary column) |  | The instrument has good precision. The method is accurate, reliable, reproducible and stable. The method was successfully applied to fingerprint analysis of 20 batches of different Baizhu. | (Yang, Qi, Ai, & Lu, 2016) |
| Stir-frying Baizhu with wheat bran, soil fried Baizhu | Ultrasonic extraction of methanol 30 min | UPLC(Acquity UPLC-BEH C18） | 240 nm | The method has strong specificity, stability and reliability, and good repeatability. Greatly reducing the analysis time. It can be used to comprehensively and systematically evaluate the quality of Baizhu manufactured products and ensure their stability. | (Sheng, Yan, Mu, Bai, & Yin, 2017) |
| Atractylodes lactone Ⅰ, Atractylodes lactone Ⅱ, Atractylodes lactone Ⅲ | Ultrasonic extraction of methanol 20 min | UPLC (Waters ACQUITY UPLC CSH C18) | 240 nm | The established method is simple, reliable, precise, stable and reproducible. The differences of Baizhu from different producing areas were analyzed by similarity and principal components. | (Yao, Wang, Peng, Wu, Wu, & Fang, 2018) |
| Atractylodes lactone Ⅰ, Atractylodes lactone Ⅱ, Atractylodes lactone Ⅲ and atractylon | Ultrasonic extraction of methanol | High-performance liquid chromatography-diode array detection，HPLC-DAD (Zorbax Eclipse XDB-C18) | 220，276nm | Atractylodes lactone Ⅰ, Atractylodes lactone Ⅱ, Atractylodes lactone Ⅲ and atractylon in Baizhu were determined simultaneously. The method was simple, efficient, accurate and stable. | (Wang, Zhou, Yang, Du, Ge, & Li, 2020b) |
| Triterpenes, Polyphenol, Polysaccharide of Baizhu | Ultrasonic extraction of methanol 30min | Technique for Order Preference by Similarity to an Ideal Solution (TOPSIS) |  | The results of this study revealed the effects of different drying methods on the chemical components of Baizhu, and provided a new idea and basis for the establishment of comprehensive quality evaluation methods and quality formation mechanism of Baizhu. | (Yang, Chen, Qian, Li, Chen, Sha, & Luo, 2021) |
| Atractylodes lactone Ⅰ, Ⅱ, Ⅲ | Ultrasonic extraction of methanol 50 min | TLC and UPLC | 235 nm | The established TLC identification method for Baizhu has strong specificity, good repeatability, simple operation and high sensitivity, and the combination of the three lactones can be better used for the quality evaluation of Baizhu. | (Zhao, Xu, Shen, Tian, & Qin, 2017c) |
| Atractylodes lactone I and Atractylodes lactone III | Extracted with methanol in an ultrasonic bath for 30min | Fourier  Transform Infrared Spectroscopy (FTIR) with Attenuated Total Reflectance (ATR); HPLC | 220 nm | Offers a wide range of possibilities to develop predictive models, both quantitative and qualitative, where the compositions of herbal medicine samples can be obtained by means of chemometric analysis. | (Cao, Cai, Zhang, & Cai, 2014) |
| Volatile components of Baizhu | Headspaceliquid phase microextraction (HS-LPME), Steam distillation | HS-LP-LPE/GC-MS |  | This method has the characteristics of simple operation, fast operation, low cost, small amount of organic solvent and sample, and effectively overcome HS-LPME droplets themselves volatile drop and other problems, at the same time, improve the sensitivity of relative to SD oil soluble substances. | (Liu, He, & Tang, 2013b) |
| Selina-4 (14), 7(11)-dien-8-one, Atractylodes lactone II, Atractylodes lactone III and Atractylodes lactone VII | Ultrasound-assisted extraction | GC-FID (flame ionisation detection). |  | The instrument has good precision. The method is accurate, reliable, reproducible and stable | (Shi, Guan, Tang,  Tao, & Guo, 2012) |
| Atractylodes lactone I and Atractylodes lactone III | Supercritical fluid extraction | GC-MS-SIM Determination |  | And gas chromatography-mass spectrometry single ion monitoring (GC-MS-SIM) is a significant analy-tical tool for mass spectrometry (MS) detection performed in single ion monitoring (SIM) mode which can increase the sensitivity | (Chen, Li, Yang, & Zhu, 2009a) |
